# HDAC1 and HDAC2 Restrain the Intestinal Inflammatory Response by Regulating Intestinal Epithelial Cell Differentiation

**DOI:** 10.1371/journal.pone.0073785

**Published:** 2013-09-05

**Authors:** Naomie Turgeon, Mylène Blais, Julie-Moore Gagné, Véronique Tardif, François Boudreau, Nathalie Perreault, Claude Asselin

**Affiliations:** Département d’anatomie et Biologie Cellulaire, Faculté de Médecine et des Sciences de la Santé, Pavillon de recherche appliquée sur le cancer, Université de Sherbrooke, Sherbrooke, Québec, Canada; Massachusetts General Hospital, United States of America

## Abstract

Acetylation and deacetylation of histones and other proteins depends on histone acetyltransferases and histone deacetylases (HDACs) activities, leading to either positive or negative gene expression. HDAC inhibitors have uncovered a role for HDACs in proliferation, apoptosis and inflammation. However, little is known of the roles of specific HDACs in intestinal epithelial cells (IEC). We investigated the consequences of ablating both HDAC1 and HDAC2 in murine IECs. Floxed *Hdac1* and *Hdac2* homozygous mice were crossed with villin-Cre mice. Mice deficient in both IEC HDAC1 and HDAC2 weighed less and survived more than a year. Colon and small intestinal sections were stained with hematoxylin and eosin, or with Alcian blue and Periodic Acid Schiff for goblet cell identification. Tissue sections from mice injected with BrdU for 2 h, 14 h and 48 h were stained with anti-BrdU. To determine intestinal permeability, 4-kDa FITC-labeled dextran was given by gavage for 3 h. Microarray analysis was performed on total colon RNAs. Inflammatory and IEC-specific gene expression was assessed by Western blot or semi-quantitative RT-PCR and qPCR with respectively total colon protein and total colon RNAs. HDAC1 and HDAC2-deficient mice displayed: 1) increased migration and proliferation, with elevated cyclin D1 expression and phosphorylated S6 ribosomal protein, a downstream mTOR target; 2) tissue architecture defects with cell differentiation alterations, correlating with reduction of secretory Paneth and goblet cells in jejunum and goblet cells in colon, increased expression of enterocytic markers such as sucrase-isomaltase in the colon, increased expression of cleaved Notch1 and augmented intestinal permeability; 3) loss of tissue homeostasis, as evidenced by modifications of claudin 3 expression, caspase-3 cleavage and Stat3 phosphorylation; 4) chronic inflammation, as determined by inflammatory molecular expression signatures and altered inflammatory gene expression. Thus, epithelial HDAC1 and HDAC2 restrain the intestinal inflammatory response, by regulating intestinal epithelial cell proliferation and differentiation.

## Introduction

Continuous intestinal epithelial cell renewal is sustained by crypt stem cells generating multiple IEC lineages [1]. Differentiation and maintenance of intestinal stem cells is regulated by different pathways, including the Notch pathway which controls secretory cell and enterocyte determination [2]. While absorptive enterocytes, mucin-producing goblet cells and enteroendocrine cells reside in small intestinal villi, antimicrobial peptide-secreting Paneth cells remain in the crypts. The colonic epithelium contains colonocytes as well as goblet and enteroendocrine cells, without Paneth cells. All gut epithelium lineages contribute to mucosal barrier function. This barrier is both physical, with the presence of tight junctions [3], and chemical, through production of mucins and the mucus layer by goblet cells [4], and of antimicrobial proteins by Paneth cells as well as other IECs, including enterocytes and goblet cells [5]. In addition to this barrier function, epithelial cells translate signals coming from intestinal luminal contents, including the microbiota, to different immune cells, in order to maintain intestinal homeostasis [6]. For example, while the mucous layer limits bacterial colonization at IEC surfaces, Paneth cells, enterocytes and colonocytes relay microbiota-derived signals in order to induce antimicrobial peptide production [7]. Thus, IECs, bacteria and immune cells communicate to insure intestinal homeostasis. However, disruption of various mechanisms preserving this equilibrium may lead to inappropriate inflammatory responses observed in inflammatory bowel diseases [8,9].

Whereas many pathways involved in the regulation of murine intestinal differentiation, proliferation and homeostasis have been discovered, the extent of epigenetic dependent transcriptional mechanisms such as acetylation and the role of various acetylation regulators, including histone deacetylases (HDAC), remain to be fully determined. Lysine-targeted acetylation and deacetylation of histones and non-histone proteins are regulated respectively by histone acetyltransferases (HAT) and HDAC [10]. Histone acetylation decreases histone interactions with DNA, resulting in relaxed chromatin, and creates docking sites for bromodomain containing proteins, which ultimately affect chromatin structure [11]. Protein acetylation levels are regulated by HDACs, which remove acetyl groups from histones to stimulate chromatin condensation, and from non-histone proteins, resulting in either gene repression or gene activation. Indeed, transcriptomic experiments suggest that HDACs display repressive as well as activating transcriptional activities, depending on the promoter and chromatin context [11]. HDACs are divided in four classes. Of these, ubiquitously expressed and highly homologous nuclear class I HDAC1 and HDAC2 form homo- or heterodimers, and are recruited to chromatin as part of large Sin3, CoREST and NuRD multiprotein complexes, among others [12,13]. These complexes contain additional chromatin-modifying activities, such as the LSD1 H3K4 demethylase in CoREST complexes, and the MI-2 chromatin remodelling enzyme in NuRD complexes.

HDAC1 and HDAC2 display both overlapping and non-redundant functions [14]. Indeed, while HDAC1 deficiency leads to pre-natal death and proliferative defects in mice, HDAC2 knockout results in perinatal lethality and cardiac arrhythmias [15]. HDAC1, and to a lesser extent HDAC2, is a negative regulator of cell proliferation [14]. HDAC inhibitors and down-regulation of specific HDACs, including HDAC1 and HDAC2, inhibit colon cancer cell proliferation [16] and modulate both inflammation and immunity [17]. Acetylated targets include, in addition to histones, transcription factors which may be acetylated by HATs and deacetylated by HDACs. For example, both HDAC3 and HDAC1 deacetylate the p65 NF-κB subunit, leading to decreased acetylation and transcriptional activity during inflammation [18,19].

To determine specific roles for HDAC1 and HDAC2 in the intestinal epithelium, we produced IEC-specific conditional mutant mice for both genes. We show that HDAC1/2 depletion in IEC alters intestinal organ growth, with defects in intestinal architecture and intestinal cell fate determination. We show that IEC-specific deletion of both HDAC1 and HDAC2 alters Notch and mTOR signalling pathways, among others, leading to chronic inflammation and disturbed homeostasis. Our findings suggest that epithelial HDAC1 and HDAC2 restrain the intestinal inflammatory response, and regulate intestinal epithelial cell polarity, proliferation and differentiation.

## Materials and Methods

### Mice

HDAC1 and HDAC2 conditionally mutated mice were provided by Dr EN Olson (University of Texas Southwestern Medical Center, Dallas, TX) [20]. Floxed HDAC1 and HDAC2 mice were crossed with villin-Cre transgenic mice to ensure specific intestinal epithelial cell gene deletion [21]. Genomic DNA was recovered with the Spin Doctor genomic DNA kit (Gerard Biotech, Oxford, OH). Mouse genotypes were determined with already published PCR protocols [20]. Mice fed with a normal diet were kept in a pathogen free facility, tested negative for *Helicobacter* , *Pasteurella* and murine norovirus. Animal experimentation protocols were approved by the Institutional Animal Research Review Committee of the Université de Sherbrooke (protocol 074-12B).

### Histological analysis and immunofluorescence

Tissues (colon or jejunum) fixed in 4% paraformaldehyde were embedded in paraffin [22]. 5 µm sections were stained with hematoxylin and eosin for histological analysis, and with Alcian blue or Periodic Acid Schiff to stain goblet cell mucins, as done before [23] or with Best’s Carmine to stain Paneth cells. For immunofluorescence experiments, sections were rehydrated with graded ethanol series containing 100, 95, 80 and 70% xylene, and then boiled for 6 min in 10 mM citric acid. Sections were blocked in a PBS solution containing 0.1% BSA and 0.2% Triton for 30 min, before adding the following antibodies: goat anti-sucrase isomaltase and goat anti-lysozyme (1:250, Santa Cruz Biotechnology Inc., Santa Cruz, CA, USA). Primary antibodies were recognized with fluorescein-coupled secondary antibodies (Vector Laboratories, Burlington, ON, Canada) or Alexa Fluor 488 goat anti-rabbit IgG (H + L) (Life Technologies Inc, Burlington, ON, Canada) incubated for 2 h at room temperature.

### In vivo proliferation and migration assay

Mice were injected intraperitoneally with 10 ml/kg of bromodeoxyuridine (BrdU, Life Technologies, Burlington, ON, Canada) for 2 h, 14 h or 48 h, to assay respectively for proliferation, colonic migration and jejunal migration. Immunofluorescence staining was done as described by Auclair et al. [24]. A 1:50 dilution of the mouse antibody against BrdU (AB BMC 9318, Roche Diagnostics, Mississauga, ON, Canada) was incubated 45 min at 37^o^C with the intestinal sections. For proliferation, the number of labelled cells per crypt was measured. For migration, the average distance between migrating cells ant the crypt was determined.

### In vivo permeability assay

To determine intestinal permeability in mice, 60 mg/100 g body weight of 4-kDa Fluorescein Isothiocyanate (FITC)-labeled dextran (Sigma-Aldrich, Oakville, ON, Canada), were given by gavage. After 3 h, mice were killed and blood recovered. FITC serum concentrations were determined with a RF-5301 PC spectrofluorophotometer (490/525 nm) (Shimadzu Scientific Instruments, Columbia, MD, USA).

### Western blot analysis

Littermate control and HDAC1/2 conditionally mutated murine colons were homogenized in Laemmli buffer with a Tissuelyser (Qiagen, Montreal, QC, Canada). Protein concentrations were measured by the BCA method (Pierce BCA Protein Assay Kit, Thermo Scientific, Rockford, USA). 30 µg of total protein extracts were loaded on a 10% or a 15% SDS-polyacrylamide gel and electroblotted on a PVDF membrane (Roche Molecular Biochemicals, Laval, QC, Canada). Membranes were incubated for 1 h at room temperature with the following primary antibodies: rabbit anti-HDAC1 and rabbit anti-HDAC2 (Abcam Inc., Toronto, ON, Canada); mouse anti-actin (Millipore, Billerica, MA, USA); rabbit anti-phosphoS6 ribosomal protein and rabbit anti-S6 ribosomal protein, rabbit anti-cleaved caspase 3, rabbit anti-phosphoStat3 and rabbit anti-Stat3, rabbit anti-cleaved Notch1, mouse anti-Cyclin D (New England Biolabs, Mississauga, ON, Canada); rabbit anti-claudin 3 (Life Technologies, Burlington, ON, Canada). Band intensity quantification was performed with the Quantity One software (Bio-Rad Laboratories, Mississauga, ON, Canada).

### RNA expression analysis

Total RNAs from colons of control and HDAC1/2 IEC-specific knockout mice were isolated with Totally RNA kit (Life Technologies, Burlington, ON, Canada) and Rneasy Mini kit (Qiagen, Mississauga, ON, Canada). cDNAs were synthesized from 1 µg of RNA, with oligo(dT_15_) and Superscript II reverse transcriptase (Life Technologies, Burlington, ON, Canada). For semi-quantitative analysis, cDNA products were amplified with the Taq PCR Master Mix (Qiagen, Mississauga, ON, Canada) with primers designed to generate 300 to 400 bp fragment length (Table S1, http://frodo.wi.mit.edu/). cDNA amplification started with a 94^o^C cycle for 5 min, followed by 26 cycles of 1 min at 94^o^C, 45 sec beginning at 62^o^C and decreasing by 0.3^o^C every cycle, 1 min at 72^o^C, and a final cycle of 1 min at 94^o^C and 10 min at 72^o^C, as done before [25]. Relative quantification was estimated by glyceraldehyde-3-phosphate-dehydrogenase (Gapdh) amplification. Amplified PCR fragments were separated on a 1.4% agarose gel and stained with ethidium bromide. For qPCR analysis, 2 ng or 10 ng of cDNAs were used as templates for amplification with the RT^2^ SYBR Green ROX qPCR Master Mix (Qiagen, Mississauga, ON, Canada) and specific gene primers (Table S1). cDNA amplification started with a 95^o^C cycle for 10 min, followed by 40 cycles of 10 sec at 95^o^C, 10 sec at 60^o^C, and 20 sec at 72^o^C. Relative quantification was estimated by porphobilinogen deaminase (Pbgd) amplification.

### Microarray analysis

Total RNAs from the colon of three control and three HDAC1/2 IEC-specific knockout mice were isolated with the Rneasy kit (Qiagen, Mississauga, ON, Canada). cDNA preparation and microarray assay were performed at the Microarray platform of the McGill University and Genome, Quebec Innovation Centre. An Affimetrix GeneChip mouse genome 430 2.0 array, displaying over 34,000 murine gene sequences, was used for hybridization. Data analysis, normalization average difference and expression measurements were subsequently completed with Flexarray software version 1.6.1. Background correction and normalization were assessed with a multi-array average (RMA) algorithm. Significant statistical differences were calculated with Welch’s t test, with the cut-off for statistical significance set to a *p* value below 0.05. Classification of genes according to their Gene Ontology biological processes was performed with the Database for Annotation, Visualization and Integrated Discovery (DAVID) v 6.7 (http://david.abcc.ncifcrf.gov/) [26] and the ToppGene suite for functional gene enrichment analysis and candidate gene priorization (http://toppgene.cchmc.org/) [27]. Both analysis tools gave similar results regarding biological processes, and the highest gene count and lowest *p* value were selected. Microarray data have been deposited in the Gene Expression Omnibus database (GSE47745).

### Statistical analysis

Statistical analyses for all experiments were calculated with the Student two-tailed t-test or one-way ANOVA (GraphPad Prism 5 software, Irvine, CA, USA). Differences were considered significant at * p≤.05, ** p≤.01, *** p≤.005 or **** p≤.001. Error bars indicate the SEM.

## Results

### Conditional intestinal epithelial HDAC1/2 loss alters small intestine and colon architecture

**Figure 1:**
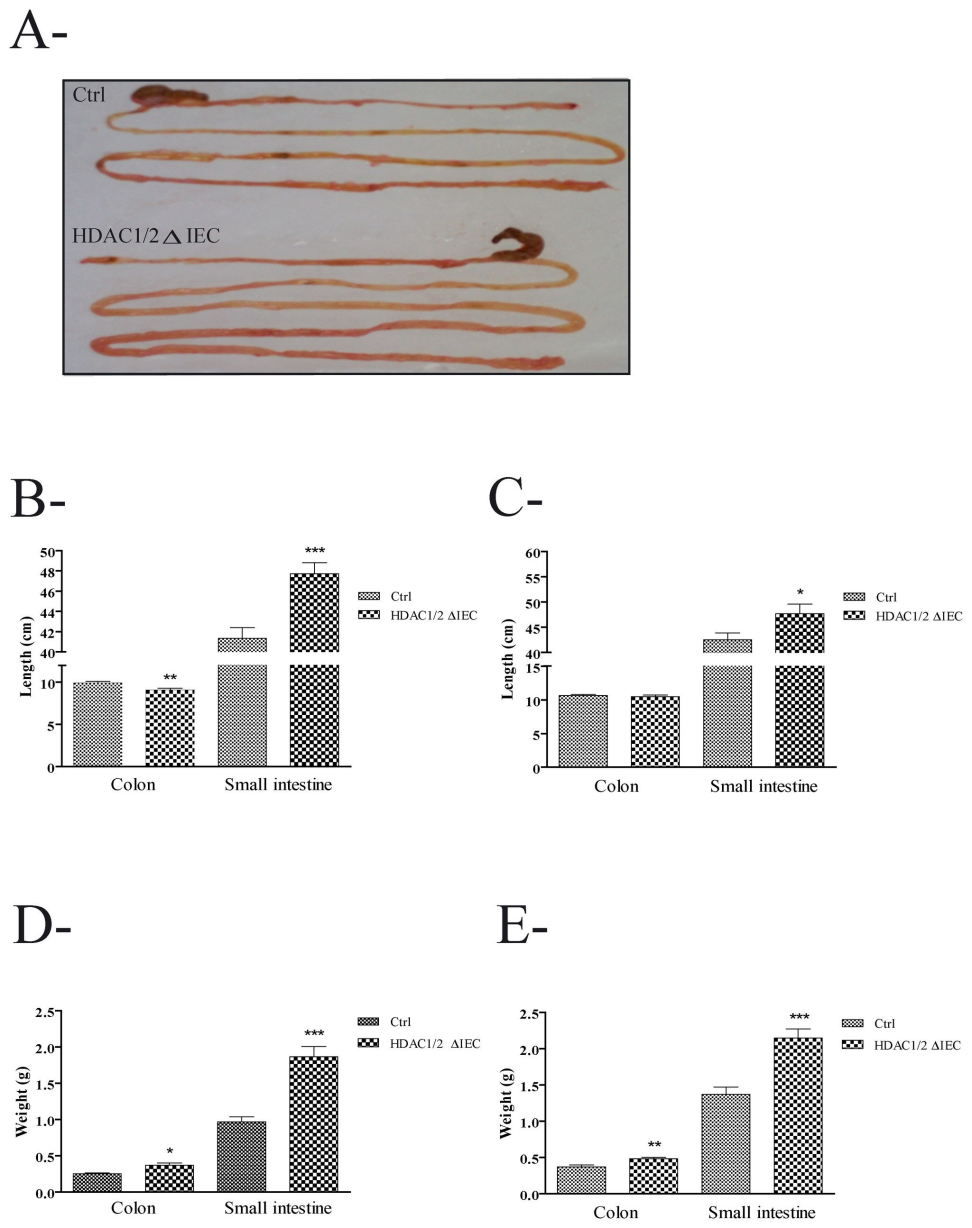
Conditional intestinal epithelial HDAC1/2 loss alters small intestine and colon size. **A**. Representative example of four-month-old control (Ctrl) and intestinal epithelial HDAC1/2 deficient (HDAC1/2ΔIEC) intestines. **B**, **C**. Small intestine and colon length of four-month-old (n=12-18) (**B**) or one-year-old (n=11-12) (**C**) control (Ctrl) and conditional intestinal epithelial HDAC1/2 ((HDAC1/2ΔIEC) mice was measured. Results represent the mean ± SEM (*p≤0.05; **p≤0.01; *** p≤0.005). **D**, **E**. Small intestine and colon weight of four-month-old (n=7-10) (**D**) or one-year-old (n=9-12) (**E**) control and intestinal epithelial HDAC1/2 deficient mice was measured. Results represent the mean ± SEM (*p≤0.05; **p≤0.01; ***p≤0.005).

While HDAC1 deletion in mice leads to embryonic lethality, HDAC2 deletion causes perinatal death, suggesting non-redundant functions [28]. However, independent conditional tissue-specific deletions of HDAC1 or HDAC2 in various tissues, such as heart and brain among others, did not display apparent phenotypes in contrast to HDAC1/2 dual deletion, suggesting partly redundant functions during post-natal development [15]. In order to determine the roles of both HDAC1 and HDAC2 and to determine the complete phenotype, we created double HDAC1/2 IEC specific knockout mice by crossing HDAC1/2 floxed mice [20] with villin-Cre transgenic mice [21]. The villin promoter sustains transgene expression from E15.5 in small intestinal and colonic epithelial cells, including stem cells [22,29]. While HDAC1/2 IEC-specific null mice appeared normal and survived for more than a year, mutant mice displayed looser than normal stools. Both 4- to -5-month-old male and female HDAC1/2 IEC deficient mice weighed less than wild-type mice, with a 10 to 13% decrease in weight (Figure S1). HDAC1/2 depletion was confirmed by Western blot analysis of Matrisperse-enriched IEC (Figure S2). We performed immunofluorescence studies of colon and jejunum from four-month-old and one-year-old control and mutant mice. While HDAC1 expression was undetectable in the murine epithelium, HDAC2 expression was patchy. Indeed, while most epithelial crypt and villus cells were negative for HDAC2 staining, we still observed some crypts and villi expressing HDAC2. This patchy expression pattern was observed to the same extent in four-month-old and one-year-old mutant mice (data not shown). Macroscopic analysis showed that HDAC1/2 depletion resulted in an increase in intestine length (Figure 1A). We thus measured small intestine or colon length and weight after four months and one year. Small intestine length and weight were significantly increased in mutant mice after four months (Figure 1B, 1D) and one year (Figure 1C, 1E) by respectively 12% and 57%. Interestingly, in contrast to one-year-old mutant mice, colon length from four-month-old mice specifically depleted in IEC HDAC1/2 was significantly decreased (Figure 1B, 1C), while colon weight was increased in both four-month-old and one-year-old mutant mice by 40% (Figure 1D, 1E). Thus, HDAC1/2 depletion in IEC alters intestinal organ growth.

**Figure 2:**
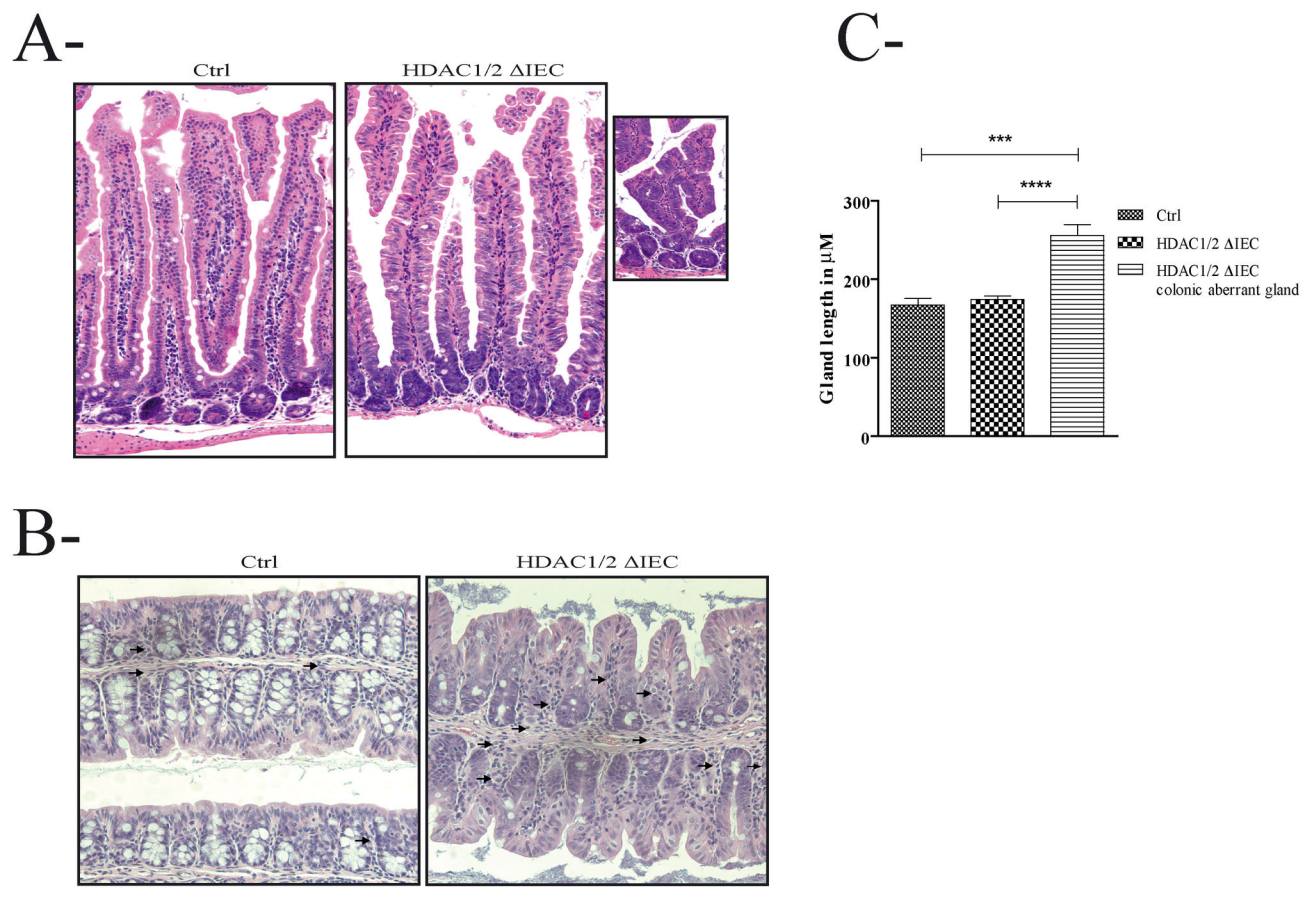
Conditional intestinal epithelial HDAC1/2 loss alters intestinal architecture. Tissue sections from four-month-old control (Ctrl) and conditional intestinal epithelial HDAC1/2 (HDAC1/2ΔIEC) jejunum (**A**) and colon (**B**) were stained with hematoxylin and eosin. A branched villus is shown in the insert. Immune cells are indicated by arrows. Magnification: 20 X or 40 X (insert). **C**. Four-month-old colonic crypt length was measured (n=4-9, 20 to 40 crypts each). Results represent the mean ± SEM (one-way ANOVA, **** p≤0.001).

Hematoxylin and eosin staining showed well stained, well aligned and basally located nuclei in the jejunal and colonic epithelium of control mice (Figure 2A, 2B). In contrast, both jejunal and colonic mutant epithelia displayed larger disorganized cells, with apparently looser cell to cell interactions. The mutant epithelium looked thicker, with some evidence of colonic infiltration of immune cells, as opposed to control epithelium (Figure 2B, arrows). Epithelial nuclei were bigger, with less defined staining, and were haphazardly located, suggesting loss of polarity. Thus, HDAC1/2 deficient jejunal and colonic mucosa was dysplastic and hyperplastic, with the presence of expanded crypts, branched villi in the jejunum (Figure 2A), villus-like structures and cell infiltrates in the colon (Figure 2B). We observed an increase in jejunal villus and crypt length in mutant mice (data not shown), and in colonic gland length in distinct regions (Figure 2C). Thus, intestinal epithelial HDAC1/2 depletion leads to defects in intestinal architecture.

### Conditional intestinal epithelial HDAC1/2 loss leads to increased proliferation and migration

**Figure 3:**
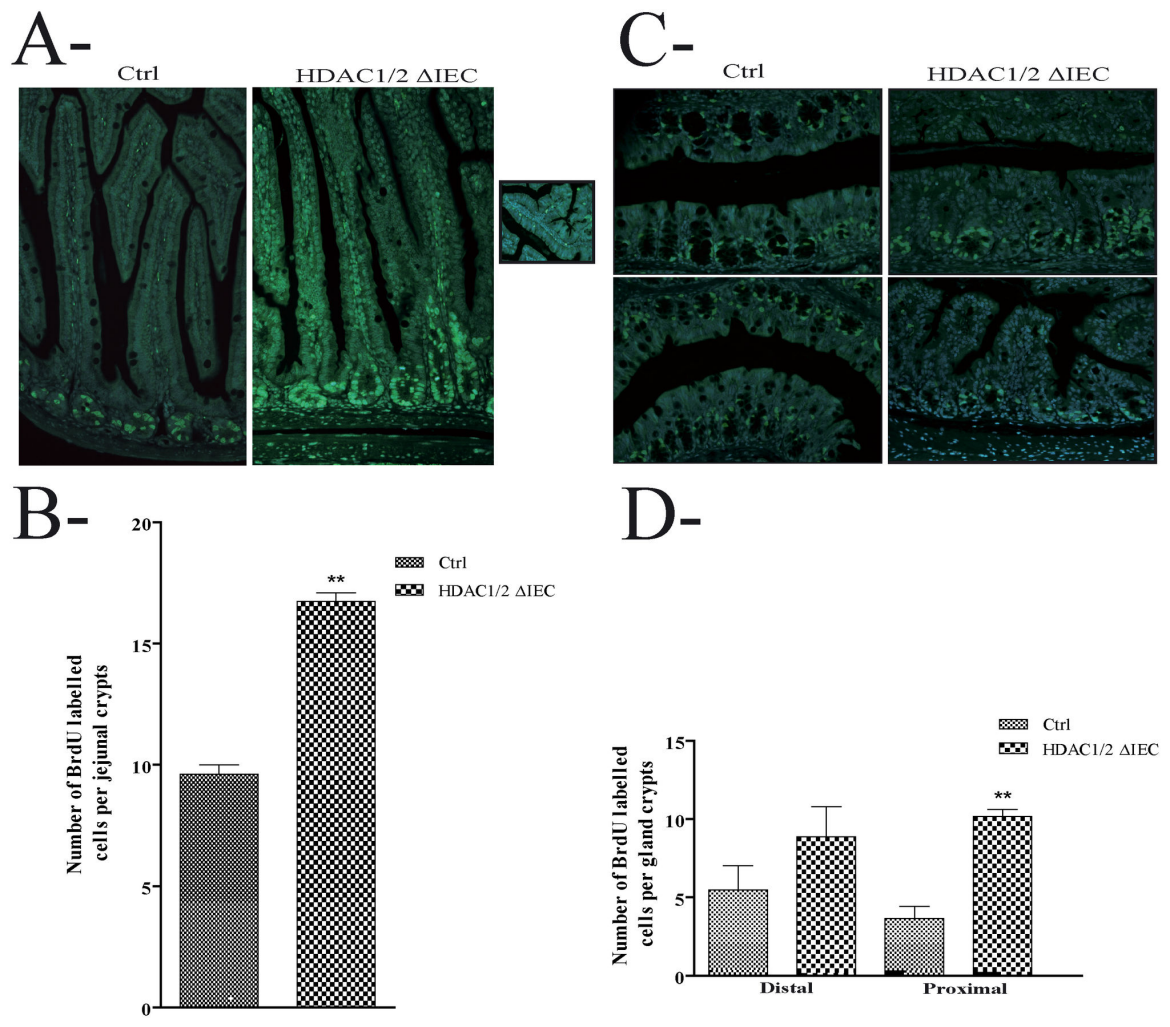
Conditional intestinal epithelial HDAC1/2 loss leads to increased proliferation. 2 h after BrdU intraperitoneal injection, four-month-old jejunal (**A**) or colonic (**B**) tissue sections from control (Ctrl) or conditional intestinal epithelial HDAC1/2 (HDAC1/2ΔIEC) mice were revealed with an antibody against BrdU. The insert in **A** shows the absence of BrdU-labelled cells in branched villi. The average number of BrdU-labelled cells per jejunal (**C**) or proximal and distal colonic (**D**) crypts was measured (n=3; 20 to 30 crypts each). Results represent the mean ± SEM (**p≤0.01). Magnification: 20 X.

**Figure 4:**
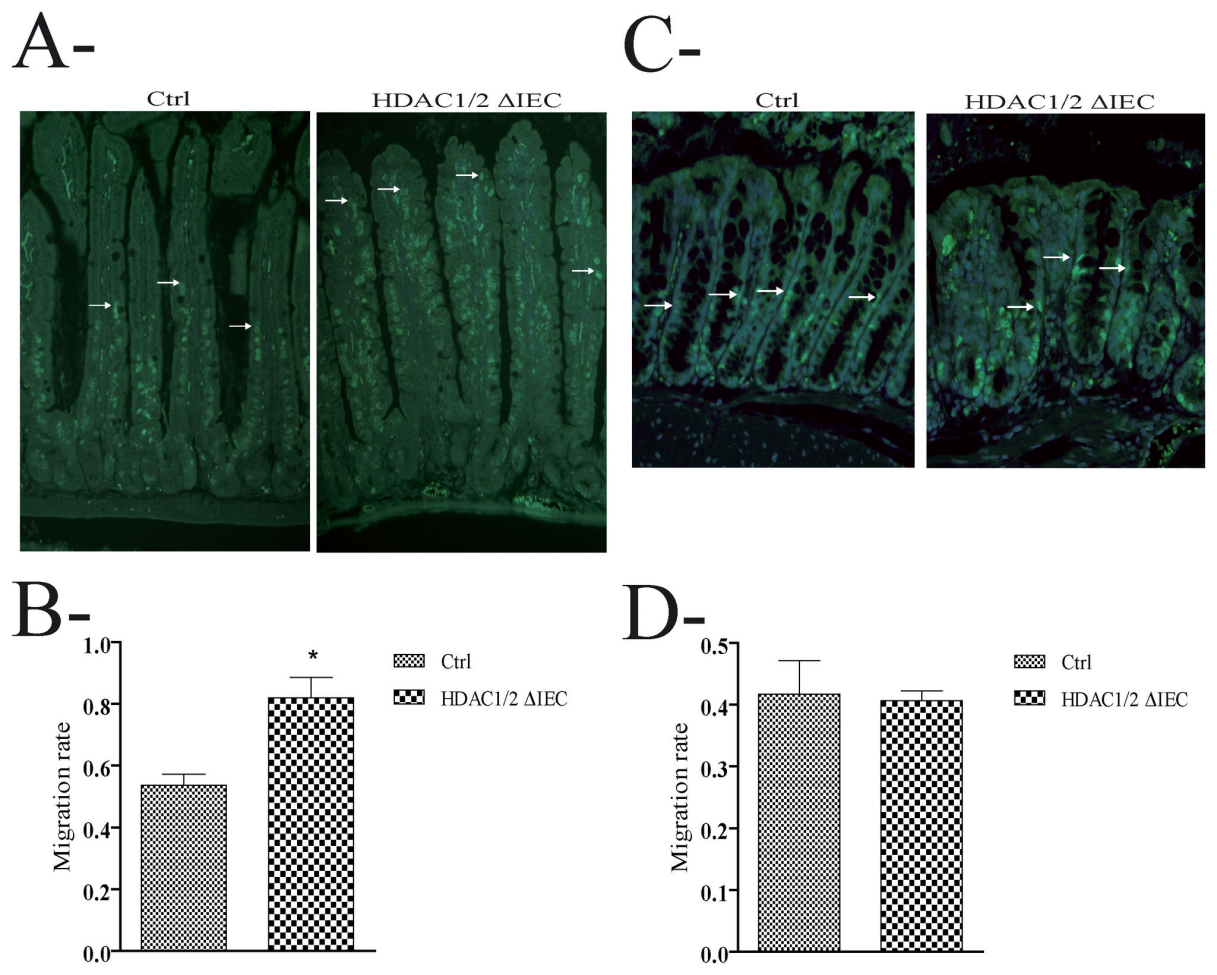
Conditional intestinal epithelial HDAC1/2 loss leads to increased migration. Four-month-old control (Ctrl) or conditional intestinal epithelial HDAC1/2 (HDAC1/2ΔIEC) mice were killed 48 h after BrdU peritoneal injection to determine jejunal migration (**A**) and 14 h after BrdU injection for colonic migration (**B**). Jejunal (**A**) or colonic (**B**) tissue sections were revealed with an antibody against BrdU. The average distance of BrdU-labelled cells from jejunal (**C**) or colonic (**D**) crypts was measured (n=3; 20 to 40 villi or colonic glands each). BrdU labelled cells are indicated by arrows. Results represent the mean ± SEM (*p≤0.05). Magnification: 20 X.

Based on our above data suggesting defects in IEC proliferation and migration in the absence of IEC HDAC1/2, we assessed proliferation by determining the level of BrdU incorporation in proliferative cells. As opposed to controls, BrdU-labelled cell numbers were increased 1.7-fold in mutant jejunum (Figure 3A, 3B) and respectively 1.6- and 2.8-fold in mutant distal and proximal colonic (Figure 3C, 3D) glands. In addition, in contrast to colon (Figure 4C, 4D), IEC migration was increased 1.4-fold in jejunum (Figure 4A, B).

**Figure 5:**
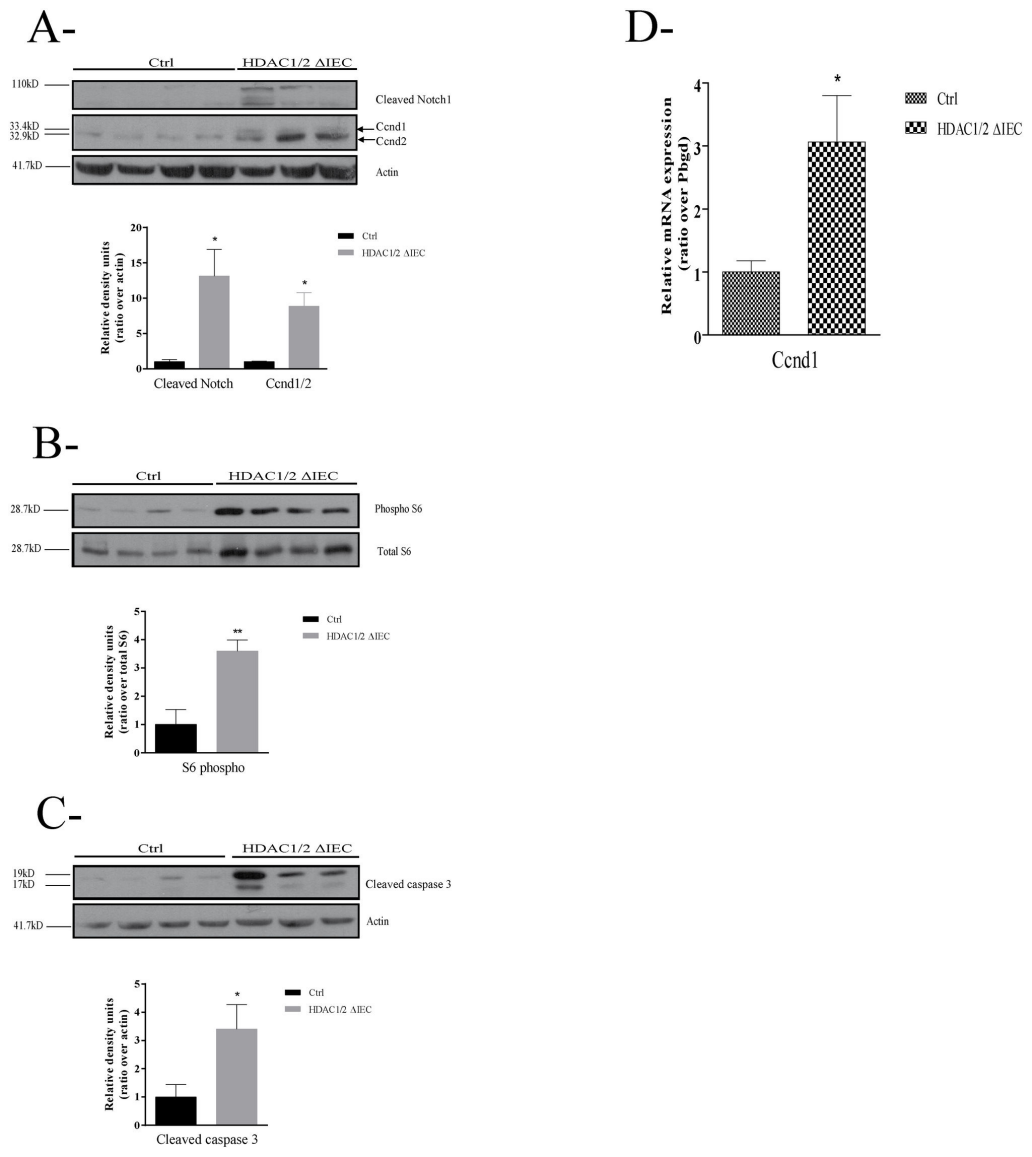
Conditional intestinal epithelial HDAC1/2 loss leads to altered activation of cell homeostasis regulators. Total protein extracts from three to four one-year-old control (Ctrl) or conditional intestinal epithelial HDAC1/2 (HDAC1/2ΔIEC) colons were separated on a 10% SDS-PAGE gel, transferred to a PVDF membrane and analysed by Western blot for expression of (**A**) Cyclin D (Ccnd1, MW: 33.4 kD; Ccnd2, MW: 32.9 kD), cleaved Notch1 (MW: 110 kD) and actin (MW: 41.7 kD) as a loading control; (**B**) phosphorylated and total ribosomal protein S6 (MW: 28.7 kD); (**C**) cleaved caspase 3 (MW: 17 kD), with actin as a loading control. The histograms indicate the ratio of band intensities normalized to actin (**A**, **C**) or total ribosomal protein S6 (**B**). Quantification of band intensity was performed with the Quantity One software. Results represent the mean ± SEM (*p≤0.05; **p≤0.01). **D**. Cyclin D1 (Ccnd1) increased expression was confirmed by qPCR analysis of total RNAs isolated from control or conditional intestinal epithelial HDAC1/2 colons. Results represent the mean ± SEM (*p≤0.05).

We then determined the expression of selected proliferation markers by Western blot analysis. Since proliferation defects were observed in both jejunum and colon, we focused our analysis on total colonic extracts. Intestinal epithelial HDAC1/2 deficient mice displayed increased colonic levels of the cell cycle G1/S transition protein Cyclin D both at protein and RNA levels (Figure 5A, 5D), of the cleaved form of Notch, a regulator of IEC fate determination [2] (Figure 5A) and of the phosphorylated form of ribosomal protein S6, a downstream target of the cell growth regulator mTOR [30] (Figure 5B). In addition to these proliferative signals, cell death inducing signals were increased, as assessed by the accumulation of cleaved caspase 3, an executioner of cell apoptosis (Figure 5C).

### Conditional intestinal epithelial HDAC1/2 loss disrupts cell lineage commitment

**Figure 6:**
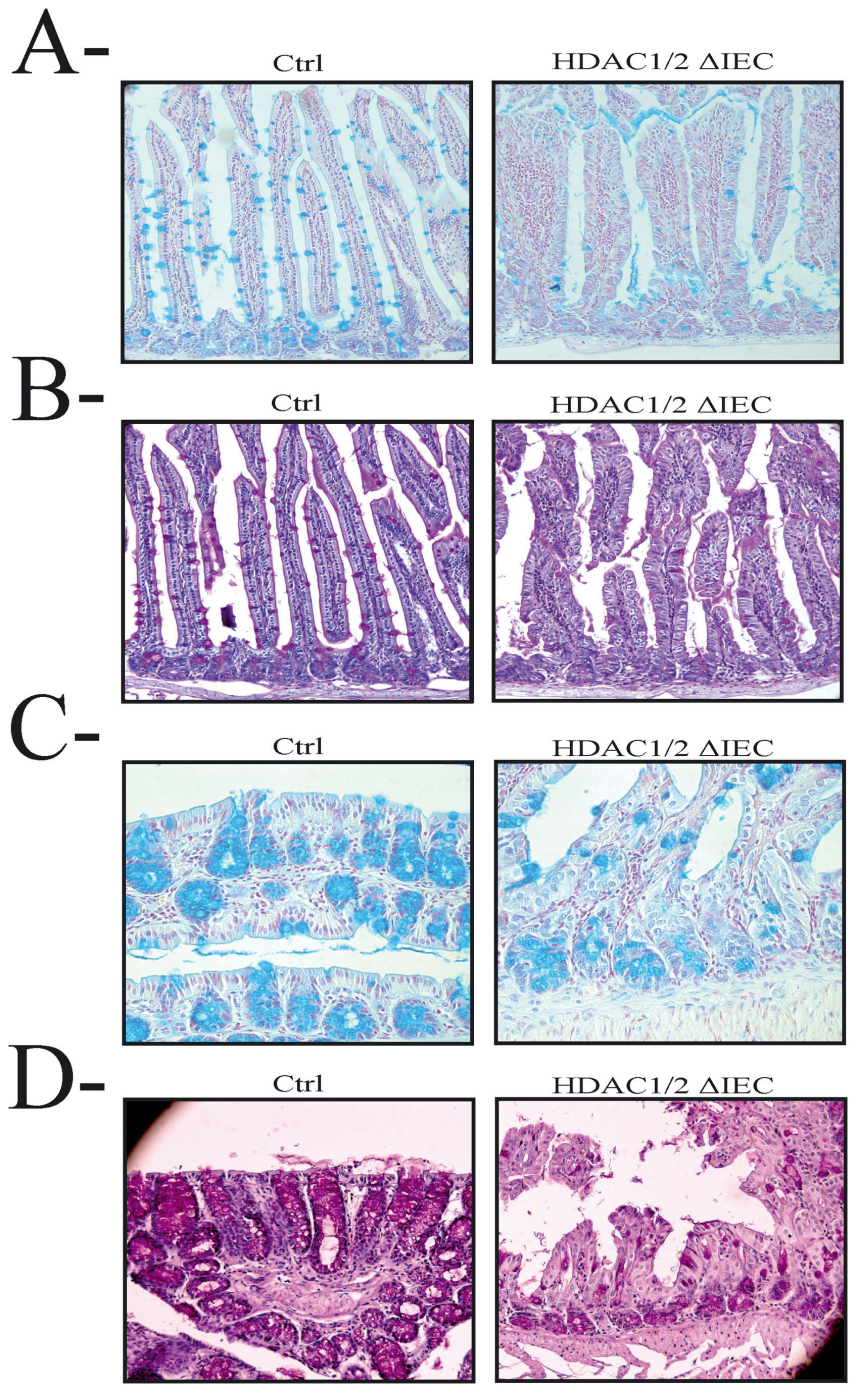
Conditional intestinal epithelial HDAC1/2 loss deregulates goblet cell differentiation. Jejunal (**A**, **B**) and colonic (**C**, **D**) tissue sections from one-year-old control (Ctrl, left panels) or conditional intestinal epithelial HDAC1/2 mice (HDAC1/2ΔIEC, right panels) mice, were stained with Alcian blue (**A**, **C**) or Periodic Acid Schiff (**B**, **D**). Magnification: 20 X.

**Figure 7:**
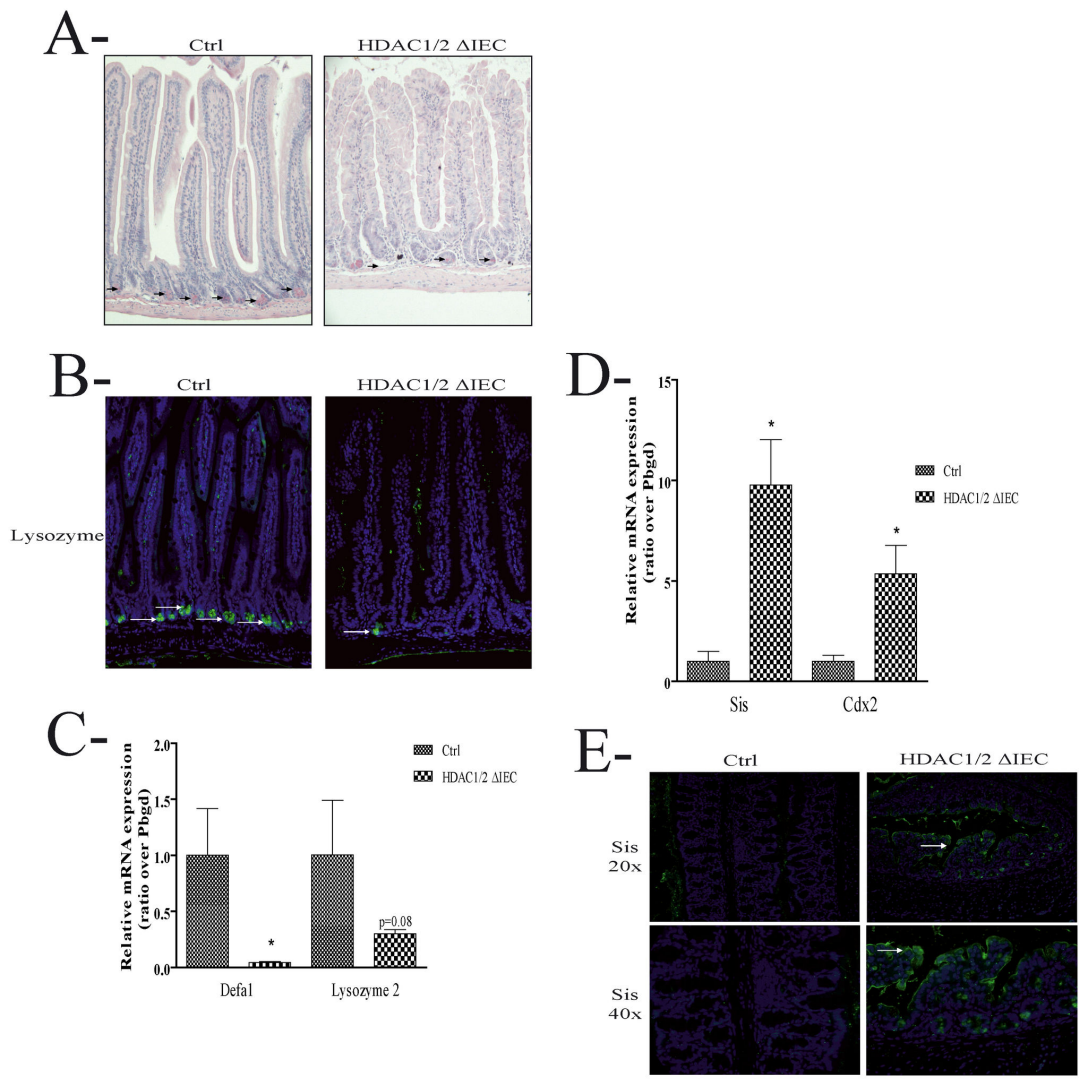
Conditional intestinal epithelial HDAC1/2 loss disrupts cell lineage commitment. **A**. Jejunal tissue sections from four-month-old control (Ctrl, left panel) or conditional intestinal epithelial HDAC1/2 (HDAC1/2ΔIEC, right panel) mice were stained with Best’s Carmine. Arrows indicate stained Paneth cells. Magnification: 20 X. **B**. Jejunal tissue sections from four-month-old control (Ctrl, left panel) or conditional intestinal epithelial HDAC1/2 (HDAC1/2ΔIEC, right panel) mice were stained with an antibody against lysozyme, a Paneth cell marker. Arrows indicate stained Paneth cells. Magnification: 20 X. C. Total RNAs were isolated from control and HDAC1/2 IEC-specific jejunum (n=4-6). Expression levels of lysozyme and Defa1 (cryptdin), two Paneth cell markers, were determined by qPCR, with Pbgd as a control. Results represent the mean ± SEM (* p≤0.05). **D**. Total RNAs were isolated from control and HDAC1/2 IEC-specific colons (n=4-5). Expression levels of Cdx2 and Sucrase-isomaltase (Sis), a small intestine enterocyte marker, were determined by qPCR, with Pbgd as a control. Results represent the mean ± SEM (* p≤0.05). **E**. Colon tissue sections from four-month-old control (Ctrl, left panel) or conditional intestinal epithelial HDAC1/2 (HDAC1/2ΔIEC, right panel) mice were stained with an antibody against Sucrase isomaltase (Sis), a small intestine enterocyte marker. Magnification: top panels: 20 X; bottom panels: 40 X.

Based on the important architectural defects observed, we hypothesized that HDAC1/2 depletion affected IEC differentiation. We thus verified the presence of secretory cells of the goblet and Paneth lineages, and of enteroendocrine cells. Decreased goblet cell numbers were observed in HDAC1/2 IEC-specific mutant mice, both in jejunum (Figure 6A, 6B) and in colon (Figure 6C, 6D), after goblet cell staining with Alcian blue (Figure 6A, 6C) and Periodic Acid Schiff (Figure 6B, 6D). Likewise, Paneth cell numbers were decreased in jejunum, as evidenced by a decrease in Best’s Carmine staining (Figure 7A) and lysozyme immunofluorescence cell staining (Figure 7B). In addition, qPCR analysis confirmed reduced jejunal expression of lysozyme and another Paneth cell marker, namely Cryptdin (Defa) (Figure 7C). While we did not observe significant differences in enteroendocrine cell numbers, both in colon and jejunum, decreased expression of the enteroendocrine marker Chga was noted, as assessed by qPCR analysis (data not shown). Finally, expression of Atoh1, an inducer of secretory cell fate, and a gene negatively regulated by the Notch pathway, is decreased, as assessed by qPCR analysis (data not shown). Thus, intestinal epithelial HDAC1/2 depletion alters secretory cell determination.

The Notch pathway, when activated, controls intestinal epithelial cell determination [2]. Indeed, when cleaved and activated, the released intracellular domain of the Notch receptor acts as a master regulator of intestinal cell fate determination by favouring enterocyte differentiation at the expense of secretory cell differentiation. We thus verified whether enterocyte determination could be induced in mutant mice, by assessing the expression of the enterocyte transcription factor Cdx2 and of a specific target, namely Sucrase-isomaltase (Sis). Sis is not expressed at significant levels in colon, as opposed to small intestine [31]. Interestingly, qPCR analysis showed an increase in Cdx2 and Sis expression in the colon (Figure 7D). Immunofluorescence studies confirmed increased Sis apical brush border expression in the colon, with however some delocalized cytoplasmic expression (Figure 7E). Thus, our data confirm a change in cell determination from a secretory to an absorptive IEC phenotype, which correlates with increased expression of cleaved Notch, a master regulator of intestinal cell fate determination, favouring enterocyte differentiation when cleaved and activated [2]. Elevated Notch signalling resulting from HDAC1/2 IEC deficiency may alter intestinal cell fate determination.

### Conditional intestinal epithelial HDAC1/2 loss disrupts epithelial barrier function

**Figure 8:**
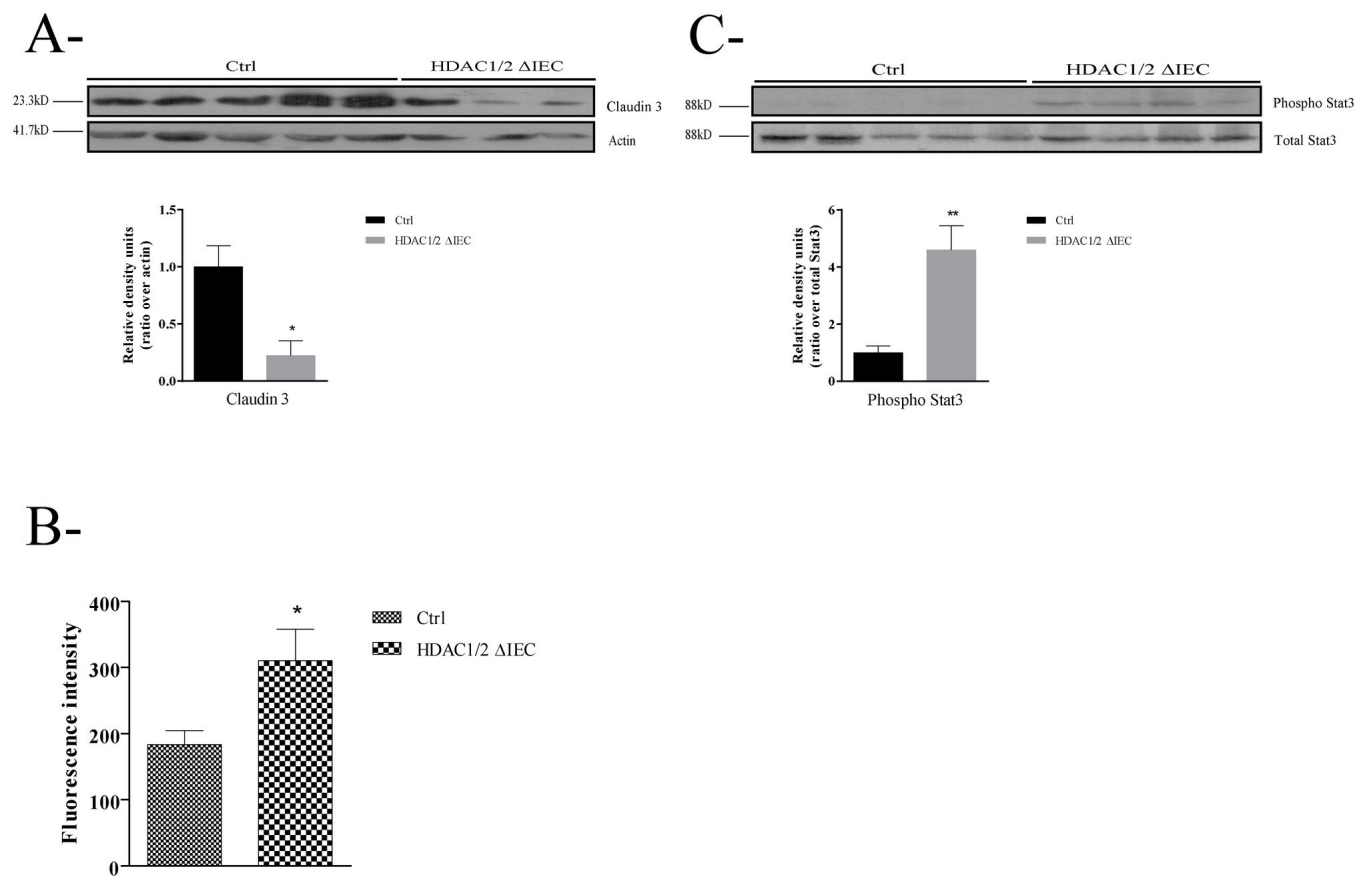
Conditional intestinal epithelial HDAC1/2 loss disrupts epithelial barrier function. **A**. Total protein extracts from three to five control (Ctrl) or conditional intestinal epithelial HDAC1/2 (HDAC1/2ΔIEC) colons were separated on a 10% SDS-PAGE gel, transferred to a PVDF membrane and analysed by Western blot for expression of Claudin 3 (MW: 23.3 kD) and actin as a loading control. The histograms indicate the ratio of band intensities normalized to actin. Quantification of band intensity was performed with the Quantity One software. Results represent the mean ± SEM (*p≤0.05). **B**. To measure intestinal permeability, blood was recovered 3 h after gavage of 4-kDa FITC-labeled dextran (n=6). FITC serum concentrations were determined with a RF-5301PC spectrofluorometer (Shimadzu Scientific Instruments, Columbia, MD, USA). Results represent the mean ± SEM (*p≤0.05). **C**. Total protein extracts from four to five control (Ctrl) or conditional intestinal epithelial HDAC1/2 (HDAC1/2ΔIEC) colons were separated on a 10% SDS-PAGE gel, transferred to a PVDF membrane and analysed by Western blot for expression of Phospho-Stat3 and total Stat3 (MW: 88 kD). The histogram indicates the ratio of Phospho-Stat3 band intensities normalized to Stat3. Quantification of band intensity was performed with the Quantity One software. Results represent the mean ± SEM (*p≤0.05).

In order to determine whether intestinal epithelial HDAC1/2 deficiency altered intestinal barrier properties, we verified the expression of one component of epithelial tight junctions, Claudin 3. Claudin 3 expression was decreased in mutant colons, as determined by Western blot analysis (Figure 8A). Epithelial tight junction component modifications could lead to altered barrier function in mutant mice. Indeed, we detected a 1.7-fold increase of 4-kDa FITC-labeled dextran-dependent fluorescence intensity in the blood of mutant mice after gavage (Figure 8B). We hypothesized that this reduced barrier function could lead to increased mucosal inflammatory response. We thus verified the state of activation of a regulator of the inflammatory response, namely Stat3 [32]. Western blot analysis showed a strong increase in phosphorylated Stat3 levels in mutant colon, as opposed to control (Figure 8C). Thus, intestinal epithelial HDAC1/2 loss may cause defects in barrier function, resulting in altered intestinal inflammatory responses.

### Conditional intestinal epithelial HDAC1/2 loss leads to modifications of inflammatory and differentiation-specific gene expression patterns

Our data suggest that HDAC1/2 IEC specific loss leads to determination defects, causing altered barrier function, as well as perturbed differentiation of secretory cells, such as goblet cells in both jejunum and colon and jejunal Paneth cells. Of note, both cell types play an important role in protecting the intestine from the intestinal microbiota. Indeed, goblet cells produce a mucus layer and secretory anti-bacterial products [4], and Paneth cells synthesize antibacterial enzymes [5]. Our results also suggest an increased inflammatory environment in the colon of HDAC1/2 IEC deficient mice. Indeed, increased immune cell infiltrates were observed. In addition, mutant mice displayed weight loss, looser than normal stools and colon shortening despite increased lengthening of the small intestine. Of note, decreased weight, looser stools and colon shortening are clinical symptoms of murine colitis [33]. To further this observation, we measured global gene expression patterns with microarray analysis by comparing total RNAs isolated from four-month-old control or HDAC1/2 IEC-specific deficient murine colons. Genes significantly expressed (p < 0.05) were selected. Interestingly, almost as many genes were increased than decreased. Indeed, over 434 known genes were increased more than two-fold (fold change (log_2_) > 1), with 101 genes increased more than four-fold (fold change (log_2_) > 2), while 352 genes were decreased more than two-fold (fold change (log_2_) < -1), with 47 genes decreased more than four-fold (fold change (log_2_) < -2) (Table S2). Functional Gene Ontology annotations were performed with the ToppGene and DAVID programs in order to classify genes according to biological processes. Categories strongly enriched (p≤0.05) and with the highest gene count were selected. The most significant classes of biological processes are represented in volcano plot of gene expression (Figure S3), and the list of genes in those classes is shown (Tables S3 to S5). Microarray analysis confirmed a chronic inflammatory response in mutant mice. Indeed, the most significantly two-fold induced groups included Immune response (p-value: 6.672E-44, gene count: 118) and Defense response (p-value: 1.948E-34, gene count: 109) for the ToppGene database, as well as Immune response (p-value: 3.E-31, gene count: 68) and Defense response (p-value: 3.8E-20, gene count: 53) for the DAVID database. Most of the categories induced were related to inflammation and the immune response. In contrast, the most significantly five-fold induced groups contained the categories Digestion (p-value: 1.338E-10, gene count: 13) for the ToppGene database, and Proteolysis (p-value: 5.80E-05, gene count: 49) for the DAVID database. Two-fold decreased biological process categories included Epithelial cell differentiation (p-value: 2.5E-05, gene count: 6) for the ToppGene, Oxidation reduction (p-value: 2.5E-05, gene count: 30) and Ion transport (p-value: 1.5E-02, gene count: 23) for the DAVID database.

**Figure 9:**
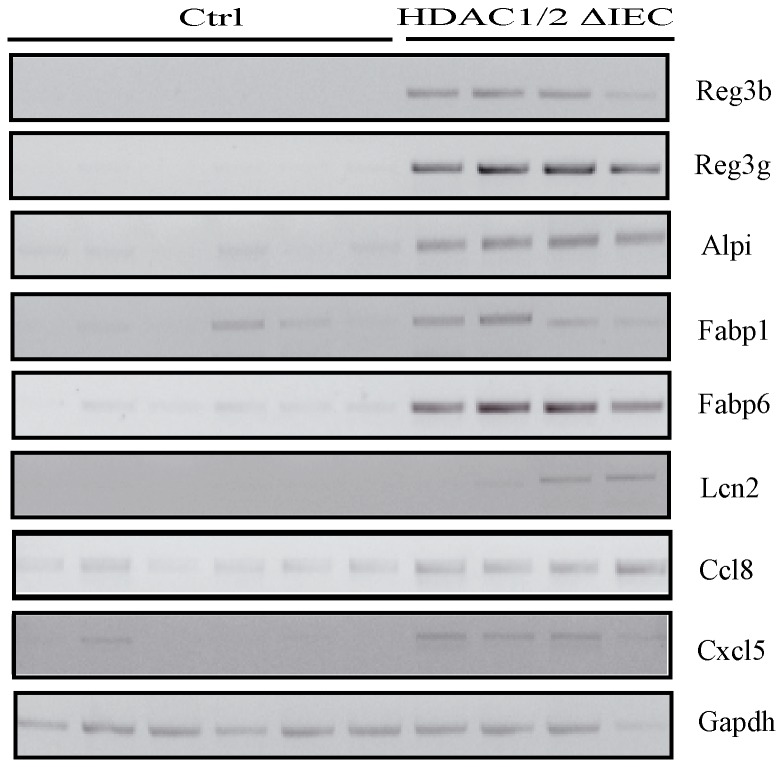
Conditional intestinal epithelial HDAC1/2 loss leads to modifications of inflammatory and differentiation-specific gene expression patterns. Total RNAs were isolated from four-month-old control and HDAC1/2 IEC invalidated colons (n=6, 4). Expression levels of selected highly induced genes, namely Reg3b, Reg3g, Alpi, Fabp1, Fabp6, Lcn2, Ccl8 and Cxcl5 were verified by semi-quantitative RT-PCR, with Gapdh as a loading control. The amplified products were separated on 2% agarose gels.

Upregulated genes included cytokines, chemokines and metalloproteases, while down-regulated genes included, among others, claudin encoding genes, such as claudin 3 (Table S2). The pattern of expression of some groups of inflammatory genes confirmed increased immune cell infiltration in HDAC1/2 IEC-deficient murine colon, such as myeloid cells. For example, microarray data show that receptors for the Fc portion of immunoglobulins (FcR) were increased significantly 2.31-fold (Fcgr2b), 2.21-fold (Fcgr3) and 3.15-fold (Fcgr4) (Table S2). These genes are considered to be expressed in monocyte-derived dendritic cells (Fcgr1), in monocytes, macrophages and neutrophils, and all myeloid populations (Fcgr2b, Fcgr3) [34]. Genes increased more than five-fold included regenerating protein family members expressed in the intestine, such as Reg1, Reg3b and Reg3g [35,36]. Reg proteins are considered as negative regulators of the inflammatory response as well as of apoptosis. From the microarray data, we thus selected genes induced more than five-fold related to inflammation (Reg3a, Reg3b, Lcn2, Ccl8, Cxcl5) and intestinal differentiation (Alpi, Fabp1, Fabp6). Of note, Alpi and Fabp6 are more expressed in the proximal parts of the intestine, such as the ileum. Semi-quantitative RT-PCR analysis confirmed the increased pattern of expression of inflammatory and intestinal genes in most IEC-specific deficient HDAC1/2 colon cDNA samples (Figure 9, left panel). In addition, the expression of CD4, a lymphocyte marker, and Tgfβ, a growth factor regulating intestinal barrier function and proinflammatory stimuli [37], was increased, as assessed by qPCR analysis (Figure S4). Thus, HDAC1/2 depletion in the murine intestine leads to deregulated proximal-to-distal gut gene expression patterns as well as increased expression of inflammatory genes, and results in chronic colon inflammation.

## Discussion

Class I HDACs, including HDAC1 and HDAC2, are ubiquitous nuclear transcriptional regulators affecting global as well as specific gene expression programs. While conditional HDAC1 or HDAC2 deletion in many tissues does not affect tissue cell viability, tissue-specific depletion of both HDAC1 and HDAC2 in mice disrupts cell differentiation and growth. For example, in the heart, dual HDAC1 and HDAC2 invalidation leads to cardiac deficiencies [20]. HDAC1 and HDAC2 specific ablation in the epidermis results in impaired epidermal regulator p63-dependent differentiation and proliferation [38], while specific knockouts in early B cell progenitors arrest B cell development at the pre-B cell stage [39]. Our data reveal an important role of both HDAC1 and HDAC2 in the regulation of intestinal epithelial cell differentiation and growth in vivo.

Homozygous IEC-specific HDAC1/2 deleted mice weigh less and survive for more than a year. Our results show a disruption of intestinal architecture, with dysplastic and hyperplastic mucosa as well as expanded crypts and branched villi. Three major phenotypic effects are observed. First, HDAC1/2 loss leads to differentiation defects with decreases in secretory Paneth and goblet cells, and increases in the number of enterocytes expressing small intestinal markers, such as sucrase-isomaltase, in the colon. This loss of secretory cell types, accompanied by induction of enterocyte markers, suggests the activation of the Notch pathway, which, by controlling stem cell fate, regulates negatively secretory cell determination and positively enterocyte determination [2,40]. Indeed, we have observed an increase in activated cleaved Notch1. Notch may be a direct target of acetylation [41]. Indeed, it was shown that the Notch1 intracellular domain (NICD) is stabilized by acetylation, leading to increased signalling [42], with reversible acetylation achieved by the Sirt1 deacetylase. Our results suggest that HDAC1 and HDAC2 could be involved directly or indirectly in modulating Notch activity, thus regulating IEC determination.

Second, HDAC1/2 impairment leads to increased intestinal growth. Small intestine weight and length, as well as colon weight, is increased, and this correlates with increased IEC proliferation and migration, as assessed by in vivo BrdU labelling experiments. Increased IEC proliferation may stem in part from Notch activation. Indeed, expression of an activated Notch1 receptor in IECs leads to increased BrdU positive cells [43,44], while Notch1 and Notch2 receptor double knockout in IECs leads to a reduced number of proliferating cells [45]. In addition, Notch activity is required for intestinal epithelial regeneration following DSS-induced colitis [46]. Another pathway which could be involved in IEC proliferation is the mTOR pathway, as suggested by increased phosphorylation of ribosomal protein S6, a downstream target of the S6kinase which is activated by mTOR. The mTOR kinase senses cellular nutrient and energy levels, and stimulates cell growth accordingly [47,48]. Acetylation may regulate mTOR signalling. Indeed, acetylation of the catalytic subunit of the AMP-activated kinase, Prkaa1, a negative regulator of mTORC1 [49], inhibits AMPK activity [50]. In contrast, AMPK deacetylation by HDAC1 increases Lkb1 kinase-dependent phosphorylation and activation of AMPK. Thus, HDAC1/2 ablation in IECs could disrupt mTOR signalling by inhibiting AMPK activity.

Third, IEC-restricted HDAC1/2 disruption leads to chronic intestinal inflammation in the colon, with colitis symptoms such as decreased weight, looser stools and colon shortening, as well as immune cell infiltrates and altered expression of immune related genes in colon, as assessed by microarray and RNA expression analysis, and elevated phosphorylation of the transcription factor Stat3, a regulator of inflammation [32]. This chronic inflammatory response may be caused by differentiation defects causing abnormal expression of cell junctional proteins. Indeed, mutant mice show increased intestinal permeability, suggesting altered barrier function. Furthermore, our data show that mutant mice display altered numbers of goblet and Paneth cells, as well as impaired expression of differentiated cell-specific genes. Goblet cells produce anti-microbial peptides and mucins which limit bacterial adhesion to the epithelium [4]. The importance of mucus for IEC homeostasis is demonstrated by the spontaneous colitis generated in Mucin 2 deficient mice [51]. Likewise, Paneth cells produce antimicrobial proteins, such as α-defensins, which limit microbial challenges and pattern resident microbial populations [5,52]. Thus, differentiation deficiencies in IEC-specific HDAC1/2 knockout mice may lead to altered responses to the microbial environment. Of note, this inflammatory environment may contribute to IEC proliferation increases observed in mutant mice, as proposed in other intestinal inflammatory models [53].

While normal intestinal homeostasis is disrupted and normal protective functions are impaired, our gene expression analysis reveals the establishment of a novel equilibrium controlling in part the inflammatory response in IEC-specific HDAC1/2 deficient mice. For example, the REG family of C-type lectins is highly expressed. One member of this family, Reg3g, expressed by IECs under inflammatory conditions, is a secreted bactericidal lectin against Gram-positive bacteria [54], which segregates the microbiota from the epithelium [55]. Another example is the increased expression of Alpi, considered a protective factor dephosphorylating bacterial lypopolysaccharides, thus reducing endotoxic responses [56] and limiting bacterial growth [57].

HDAC1 and HDAC2, as well as acetyltransferases contribute to the formation of the acetylome [58]. The acetylome is regulated by endogenous as well as exogenous signals. It has been shown that levels of the substrate donor acetyl-CoA vary according to metabolic cues such as nutrient availability, leading to different levels of acetyltransferase activities and protein acetylation [59]. In addition, HDAC activities are regulated by endogenous cell inhibitors. For example, fasting increases production of the β-hydroxybutyrate metabolite, which inhibits class I HDACs, including HDAC1 and HDAC2, leading to increased histone acetylation [60,61]. Furthermore, the acetylome is subject to regulation by the intestinal microbial environment. Acetate, produced by microbial fermentation, may directly contribute to endogenous acetyl-CoA levels [62,63]. Another microbial fermentation product, butyrate, is an HDAC inhibitor, leading to increased histone acetylation levels [64]. Finally, recent data have shown that reintroduction of gut bacteria in gnotobiotic mice increases the number of lysine acetylated proteins in colon as well as liver [65]. Thus, acetyl-CoA levels and exogenous as well as endogenous metabolites affect protein acetylation, in part by regulating HDAC activities [66]. Thus, HDAC1 and HDAC2 may contribute to the transmission of endogenous as well as exogenous signals to the IEC acetylome.

We have uncovered, for the first time, an intriguingly specific HDAC1- and HDAC2-dependent phenotype, with intestinal growth, differentiation and cell fate determination alterations in IEC-specific conditional mutant mice. We have shown that IEC-specific deletion of both HDAC1 and HDAC2 may alter Notch and mTOR signalling pathways, among others, leading to chronic inflammation and disturbed homeostasis. Our findings suggest that HDAC1 and HDAC2 restrain the intestinal inflammatory response, and regulate intestinal epithelial cell polarity, proliferation and differentiation. HDAC1 and HDAC2 may well play important roles in relaying endogenous as well as exogenous cues to IECs and the intestinal mucosa.

## Supporting Information

Figure S1 Conditional intestinal epithelial HDAC1/2 loss leads to weight reduction.
Four-month-old control and conditional intestinal epithelial HDAC1/2 male and female mice were weighed. Female Ctrl, n=13; Female HDAC1/2, n=17; Male Ctrl, n=10; Male HDAC1/2, n=17. Results represent the mean ± SEM (* p≤0.05; **p≤0.01).
(TIF)

Figure S2 HDAC1 and HDAC2 proteins are depleted in intestinal epithelial HDAC1/2 deficient cells.
Control (n=3) IECs and HDAC1/2 (n=4) deficient IECs were isolated by the Matrisperse method. 30 µg of nuclear protein extracts were separated by SDS-PAGE and transferred to PVDF membranes for Western blot analysis of HDAC1 (MW: 55.1 kD), HDAC2 (MW: 55.3 kD) and actin (MW: 41.7 kD), as a loading control.
(TIF)

Figure S3 Volcano plot of gene expression in HDAC1/2 depleted murine colons as measured from microarray analysis.
Genes with P-value < 0.05 and fold change (log_2_) > 1 or fold change (log_2_) < -1 were classified according to biological processes from GO database. Biological processes with best P-value and higher gene counts were selected and were displayed in the Volcano plot.
(TIF)

Figure S4 Conditional intestinal epithelial HDAC1/2 loss leads to modifications of inflammatory and differentiation-specific gene expression patterns.
Total RNAs were isolated from control and HDAC1/2 IEC-specific colons. Expression levels of CD4, a lymphocyte marker (A) (n=9-10), and Tgfβ (B) (n=6-5), were determined by qPCR, with Pbgd as a control. Results represent the mean ± SEM (* p≤0.05).
(TIF)

Table S1 A. Oligonucleotides used for semi-quantitative RT-PCR.
B. Oligonucleotides used for qPCR.
(DOC)

Table S2 List of 2-fold significantly induced or repressed genes in HDAC1/2-depleted murine colon, as determined by microarray analysis.
(DOCX)

Table S3 List of immune and/or defense response genes with significant 2-fold increased or decreased expression levels in HDAC1/2-depleted murine colons as determined by microarray analysis, and classified according to GO database.
(DOCX)

Table S4 List of digestion and/or proteolysis genes with significant 2-fold increased or decreased expression levels in HDAC1/2-depleted murine colons as determined by microarray analysis, and classified according to GO database.
(DOCX)

Table S5 List of epithelial or epidermal development, and differentiation genes with significant 2-fold increased or decreased expression levels in HDAC1/2-depleted murine colons as determined by microarray analysis, and classified according to GO database.
(DOCX)
