# Supplementary figures and images for: HDAC1 and HDAC2 Restrain the Intestinal Inflammatory Response by Regulating Intestinal Epithelial Cell Differentiation
Source: PLoS One. 2013 Sep 5;8(9):e73785. doi: 10.1371/journal.pone.0073785 (PMC3764035; doi:10.1371/journal.pone.0073785)

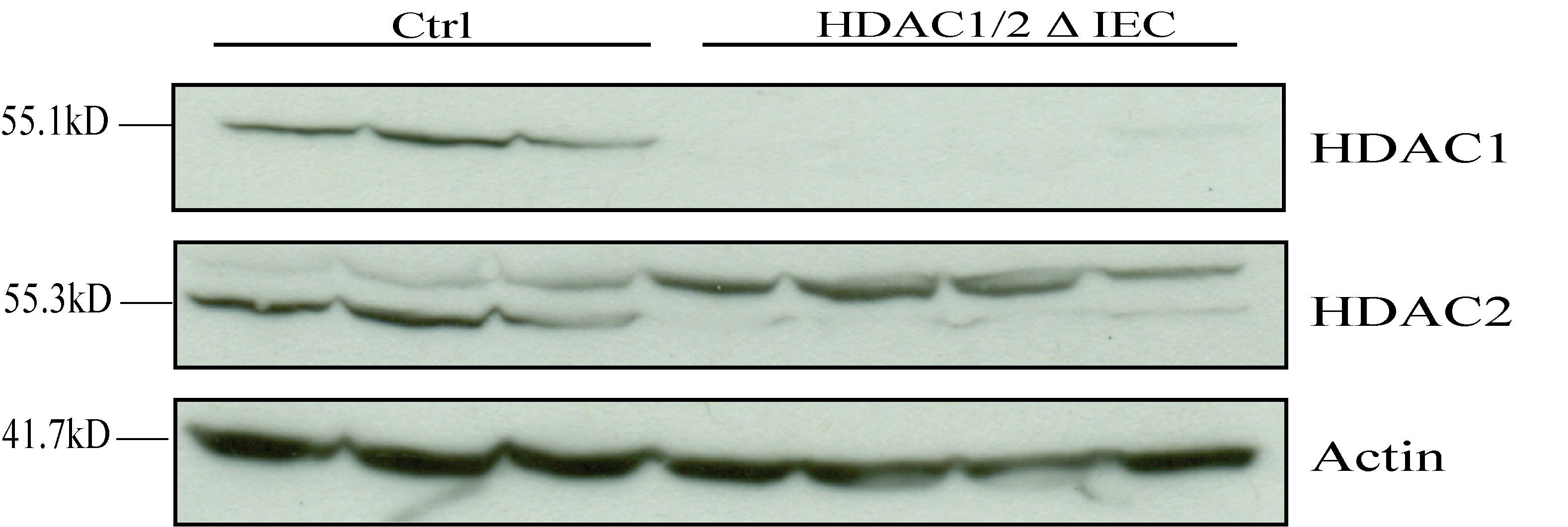

Supplement: Figure S2 — HDAC1 and HDAC2 proteins are depleted in intestinal epithelial HDAC1/2 deficient cells. Control (n=3) IECs and HDAC1/2 (n=4) deficient IECs were isolated by the Matrisperse method. 30 µg of nuclear protein extracts were separated by SDS-PAGE and transferred to PVDF membranes for Western blot analysis of HDAC1 (MW: 55.1 kD), HDAC2 (MW: 55.3 kD) and actin (MW: 41.7 kD), as a loading control. (TIF) [file pone.0073785.s002.tif]

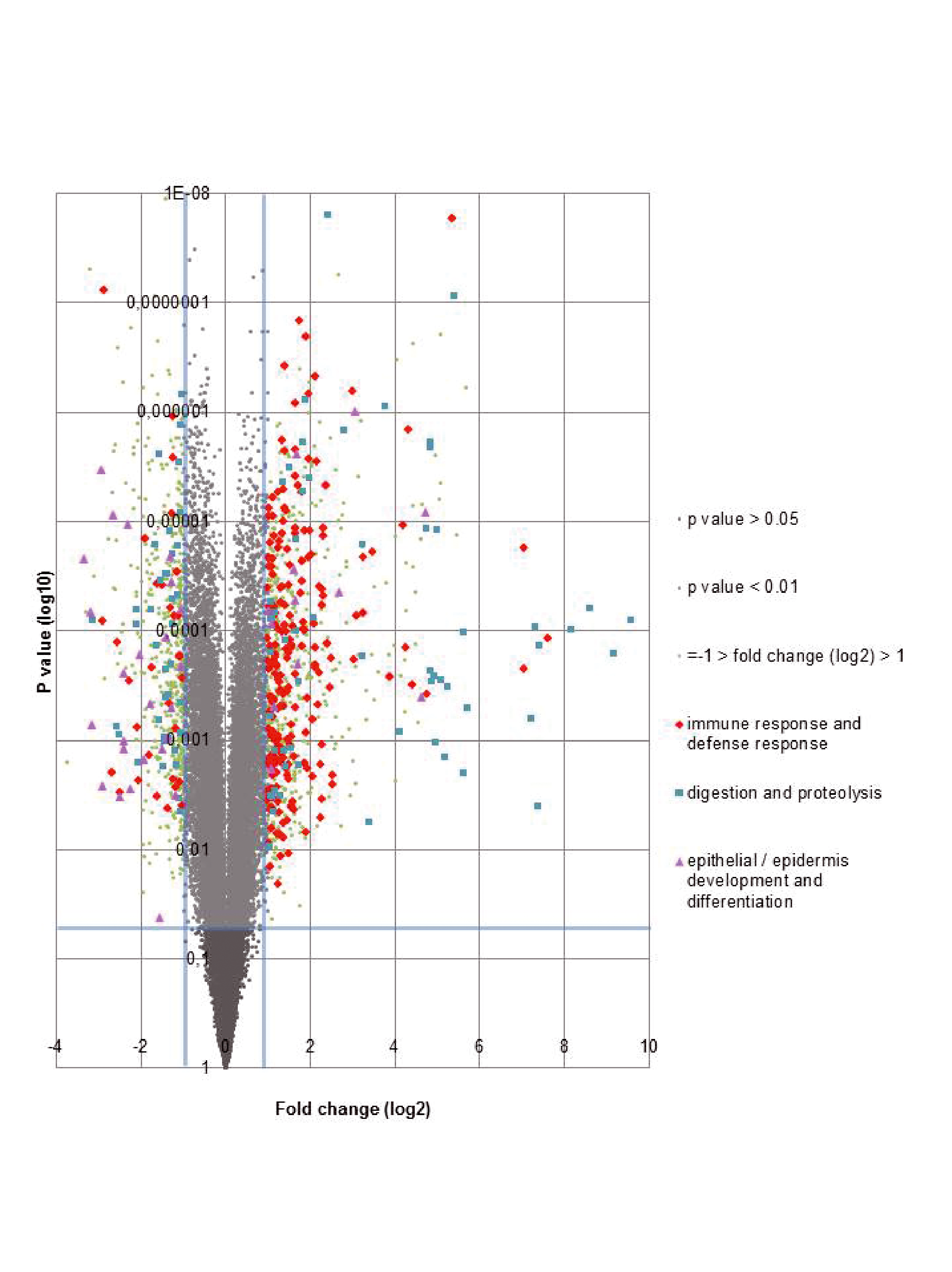

Supplement: Figure S3 — Volcano plot of gene expression in HDAC1/2 depleted murine colons as measured from microarray analysis. Genes with P-value < 0.05 and fold change (log2) > 1 or fold change (log2) < -1 were classified according to biological processes from GO database. Biological processes with best P-value and higher gene counts were selected and were displayed in the Volcano plot. (TIF) [file pone.0073785.s003.tif]

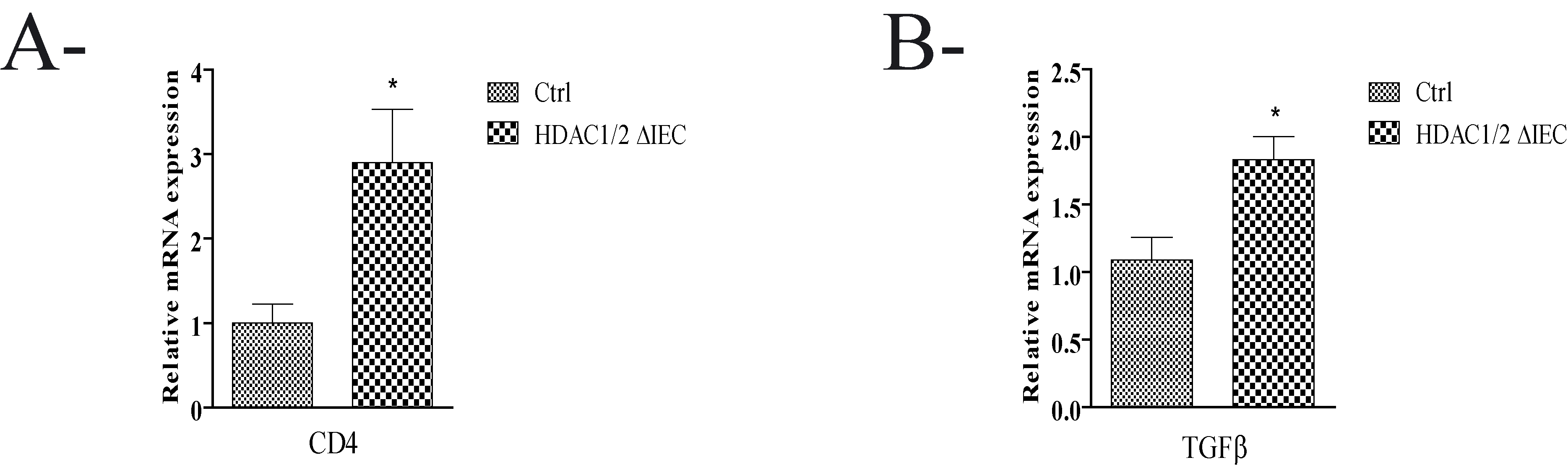

Supplement: Figure S4 — Conditional intestinal epithelial HDAC1/2 loss leads to modifications of inflammatory and differentiation-specific gene expression patterns. Total RNAs were isolated from control and HDAC1/2 IEC-specific colons. Expression levels of CD4, a lymphocyte marker (A) (n=9-10), and Tgfβ (B) (n=6-5), were determined by qPCR, with Pbgd as a control. Results represent the mean ± SEM (* p≤0.05). (TIF) [file pone.0073785.s004.tif]
