# Supplementary material for: HDAC1 and HDAC2 Restrain the Intestinal Inflammatory Response by Regulating Intestinal Epithelial Cell Differentiation
Source: PLoS One. 2013 Sep 5;8(9):e73785. doi: 10.1371/journal.pone.0073785 (PMC3764035; doi:10.1371/journal.pone.0073785)
Supplement: Table S1 — A. Oligonucleotides used for semi-quantitative RT-PCR. B. Oligonucleotides used for qPCR. (DOC) [file pone.0073785.s005.doc]

TABLE S1A. Oligonucleotide Used In This Study for RT-PCR

|  |  |  |  |
| --- | --- | --- | --- |
| Gene | UP | Down | Accession |
|  |  |  |  |
| Reg3b | 5'-CAGACCTGGTTTGATGCAGA-3' | 5'-AATTCGGGATGTTTGCTGTC-3' | NM_011036.1 |
| Reg3g | 5'-CCTGATGCTCCTTTCTCAGG-3' | 5'-CACTCCCATCCACCTCTGTT-3' | NM_011260.1 |
| Alpi | 5'-CACAGCTTACCTGGCACTGA-3' | 5'-GGTCTCTGACGACAGGGGTA-3' | NM_001081082.2 |
| Fabp1 | 5'-GTCAGCTGTGGAAAGGAAGC -3' | 5'-TGTAGACAATGTCGCCCAAT-3' | NM_017399.4 |
| Fabp6 | 5'-TCATCACAGAGGTCCAGCAG-3' | 5'-AACTTGTCACCCACGACCTC-3' | NM_008375.2 |
| Lcn2 | 5'-ATGTCACCTCCATCCTGGTC-3' | 5'-CACACTCACCACCCATTCAG-3' | NM_008491.1 |
| Ccl8 | 5'-TAAGGCTCCAGTCACCTGCT-3' | 5'-CAGGCACCATCTGCTTGTAA-3' | NM_021443.3 |
| Cxcl5 | 5'-TGCCCCTTCCTCAGTCATAG-3' | 5'-GGGACAATGGTTTCCCTTTT-3' | NM_009141.2 |
| Gapdh | 5'-CCAAAGTTGTCATGGATGAC-3' | 5'-GTGAAGGTCGGTGTGAACGG-3' | NM_017008 |
|  |  |  |  |

TABLE S1B. Oligonucleotide Used In This Study for qPCR

|  |  |  |  |
| --- | --- | --- | --- |
| Gene | UP | Down | Accession |
|  |  |  |  |
| Ccnd1 | 5'-GCGTACCCTGACACCAATCTC-3' | 5'-CTCCTCTTCGCACTTCTGCTC-3' | NM_007631.2 |
| Cryptdin | 5'-CAGCCAGGAGAAGAGGACCAG-3' | 5'-TAGCATACCAGATCTCTCAACGAT-3' | NM_010031.2 |
| Lysozyme 2 | 5'-ATGGAATGGCTGGCTACTATGGAG-3' | 5'-CTCACCACCCTCTTTGCACATTG-3' | NM_017372.3 |
| Sis | 5'-TCAAGAAATCACAACATTCAATTTACTAG-3' | 5'-CTAAAACTTTCTTTGACATTTGAGCAA-3' | NM_001081137.1 |
| Cdx2 | 5'-TCACCATCAGGAGGAAAAGTG-3' | 5'-GCAAGGAGGTCACAGGACTC-3' | NM_007673.3 |
