# Supplementary material for: HDAC1 and HDAC2 Restrain the Intestinal Inflammatory Response by Regulating Intestinal Epithelial Cell Differentiation
Source: PLoS One. 2013 Sep 5;8(9):e73785. doi: 10.1371/journal.pone.0073785 (PMC3764035; doi:10.1371/journal.pone.0073785)
Supplement: Table S2 — List of 2-fold significantly induced or repressed genes in HDAC1/2-depleted murine colon, as determined by microarray analysis. (DOCX) [file pone.0073785.s006.docx]

**Table S2**

List of 2-fold significantly induced or repressed genes in HDAC1/2-depleted murine colon, as determined by microarray analysis.

| **Probeset** | **RefSeq Transcript ID** | **Gene Symbol** | **Gene Title** | **Fold change (log2)** | **P-value** |
| --- | --- | --- | --- | --- | --- |
| 1425668_a_at | NM_009178 | St3gal4 | ST3 beta-galactoside alpha-2,3-sialyltransferase 4 | -3,75 | 1,57E-03 |
| 1438394_x_at | NM_008475 | Krt4 | keratin 4 | -3,33 | 2,18E-05 |
| 1455913_x_at | NM_013697 | Ttr | transthyretin | -3,30 | 6,69E-05 |
| 1459737_s_at | NM_013697 | Ttr | transthyretin | -3,23 | 4,87E-08 |
| 1422588_at | NM_010669 | Krt6b | keratin 6B | -3,19 | 6,74E-05 |
| 1452166_a_at | NM_010660 | Krt10 | keratin 10 | -3,15 | 7,14E-04 |
| 1454608_x_at | NM_013697 | Ttr | transthyretin | -3,13 | 3,12E-05 |
| 1425102_a_at | NM_001130513 | Ace2 | angiotensin I converting enzyme (peptidyl-dipeptidase A) 2 | -3,11 | 8,27E-05 |
| 1440150_at | NM_009374 | Tgm3 | transglutaminase 3, E polypeptide | -2,93 | 3,39E-06 |
| 1440832_at | NM_177544 | Ang4 | angiogenin, ribonuclease A family, member 4 | -2,90 | 8,17E-05 |
| 1434227_at | NM_001033131 | Krtdap | keratinocyte differentiation associated protein | -2,90 | 2,60E-03 |
| 1420431_at | NM_009100 | Rptn | repetin | -2,87 | 2,72E-04 |
| 1438364_x_at | NM_177544 | Ang4 | angiogenin, ribonuclease A family, member 4 | -2,86 | 7,67E-08 |
| 1418909_at | NM_013848 | Ermap | erythroblast membrane-associated protein | -2,77 | 1,45E-05 |
| 1418165_at | NM_010584 | Itln1 | intelectin 1 (galactofuranose binding) | -2,69 | 5,47E-06 |
| 1418480_at | NM_023785 | Ppbp | pro-platelet basic protein | -2,69 | 1,97E-03 |
| 1432902_at | --- | 9130414P19Rik | RIKEN cDNA 9130414P19 gene | -2,66 | 4,04E-05 |
| 1421355_at | NM_009374 | Tgm3 | transglutaminase 3, E polypeptide | -2,66 | 8,75E-06 |
| 1451580_a_at | NM_013697 | Ttr | transthyretin | -2,65 | 2,25E-06 |
| 1449375_at | NM_001190330 | Ces2a | carboxylesterase 2A | -2,62 | 1,97E-06 |
| 1420491_at | NM_026114 | Eif2s1 | eukaryotic translation initiation factor 2, subunit 1 alpha | -2,62 | 4,13E-05 |
| 1418113_at | NM_010005 | Cyp2d10 | cytochrome P450, family 2, subfamily d, polypeptide 10 | -2,58 | 1,47E-05 |
| 1418847_at | NM_009705 | Arg2 | arginase type II | -2,56 | 1,27E-04 |
| 1419343_at | NM_053079 | Slc15a1 | solute carrier family 15 (oligopeptide transporter), member 1 | -2,56 | 7,67E-04 |
| 1427221_at | NM_139142 | Slc6a20a | solute carrier family 6 (neurotransmitter transporter), member 20A | -2,56 | 2,02E-04 |
| 1449409_at | NM_026935 | Sult1c2 | sulfotransferase family, cytosolic, 1C, member 2 | -2,55 | 2,58E-07 |
| 1418734_at | NM_010392 | H2-Q2 | histocompatibility 2, Q region locus 2 | -2,49 | 3,04E-03 |
| 1422784_at | NM_008476 | Krt6a | keratin 6A | -2,48 | 3,26E-03 |
| 1448961_at | NM_001195084 | Plscr2 | phospholipid scramblase 2 | -2,48 | 1,58E-04 |
| 1455848_at | NM_001033233 | Tmprss11a | transmembrane protease, serine 11a | -2,48 | 8,99E-04 |
| 1448792_a_at | NM_007817 | Cyp2f2 | cytochrome P450, family 2, subfamily f, polypeptide 2 | -2,46 | 1,02E-04 |
| 1425415_a_at | NM_009199 | Slc1a1 | solute carrier family 1 (neuronal/epithelial high affinity glutamate transporter, system Xag), member 1 | -2,44 | 8,65E-05 |
| 1418709_at | NM_009944 | Cox7a1 | cytochrome c oxidase, subunit VIIa 1 | -2,42 | 5,26E-07 |
| 1422837_at | NM_022886 | Scel | sciellin | -2,41 | 2,41E-04 |
| 1449204_at | NM_010291 | Gjb5 | gap junction protein, beta 5 | -2,39 | 1,02E-03 |
| 1427268_at | XM_485270 | Flg | filaggrin | -2,38 | 1,19E-03 |
| 1417618_at | NM_010582 | Itih2 | inter-alpha trypsin inhibitor, heavy chain 2 | -2,36 | 4,48E-06 |
| 1434280_at | --- | --- | --- | -2,35 | 2,19E-04 |
| 1438558_x_at | NM_008239 | Foxq1 | forkhead box Q1 | -2,31 | 1,07E-05 |
| 1451924_a_at | NM_010104 | Edn1 | endothelin 1 | -2,29 | 2,90E-04 |
| 1422401_at | NM_011478 | Sprr3 | small proline-rich protein 3 | -2,24 | 2,79E-03 |
| 1427345_a_at | NM_133670 | Sult1a1 | sulfotransferase family 1A, phenol-preferring, member 1 | -2,24 | 1,69E-07 |
| 1451588_at | NM_026947 | 1810022C23Rik | RIKEN cDNA 1810022C23 gene | -2,23 | 2,51E-05 |
| 1425559_a_at | NM_016870 | Acsm3 | acyl-CoA synthetase medium-chain family member 3 | -2,21 | 2,06E-04 |
| 1439780_at | NM_025433 | Rpl7l1 | ribosomal protein L7-like 1 | -2,18 | 5,18E-06 |
| 1448299_at | NM_009199 | Slc1a1 | solute carrier family 1 (neuronal/epithelial high affinity glutamate transporter, system Xag), member 1 | -2,18 | 7,66E-06 |
| 1452492_a_at | NM_001145960 | Slc37a2 | solute carrier family 37 (glycerol-3-phosphate transporter), member 2 | -2,17 | 2,10E-06 |
| 1429313_at | NM_013845 | Ror1 | receptor tyrosine kinase-like orphan receptor 1 | -2,16 | 2,42E-06 |
| 1415824_at | NM_009128 | Scd2 | stearoyl-Coenzyme A desaturase 2 | -2,15 | 2,41E-04 |
| 1456475_s_at | NM_011158 | Prkar2b | protein kinase, cAMP dependent regulatory, type II beta | -2,13 | 9,81E-04 |
| 1438664_at | NM_011158 | Prkar2b | protein kinase, cAMP dependent regulatory, type II beta | -2,12 | 4,86E-05 |
| 1417991_at | NM_007860 | Dio1 | deiodinase, iodothyronine, type I | -2,10 | 4,36E-05 |
| 1460180_at | NM_010422 | Hexb | hexosaminidase B | -2,10 | 1,26E-06 |
| 1425107_a_at | NM_001113386 | Lifr | leukemia inhibitory factor receptor | -2,09 | 2,70E-05 |
| 1438841_s_at | NM_009705 | Arg2 | arginase type II | -2,09 | 7,57E-04 |
| 1457025_at | NM_001080710 | Sdr16c6 | short chain dehydrogenase/reductase family 16C, member 6 | -2,09 | 1,80E-06 |
| 1438980_x_at | NM_178079 | Pm20d1 | peptidase M20 domain containing 1 | -2,09 | 8,76E-05 |
| 1427477_at | NM_001013373 | Tmprss13 | transmembrane protease, serine 13 | -2,09 | 6,59E-05 |
| 1424968_at | NM_197999 | Ces2g | carboxylesterase 2G | -2,09 | 1,06E-04 |
| 1418095_at | NM_025357 | Smpx | small muscle protein, X-linked | -2,09 | 1,25E-06 |
| 1452138_a_at | NM_001130513 | Ace2 | angiotensin I converting enzyme (peptidyl-dipeptidase A) 2 | -2,06 | 1,61E-03 |
| 1449838_at | NM_009639 | Crisp3 | cysteine-rich secretory protein 3 | -2,05 | 2,36E-03 |
| 1419595_a_at | NM_010281 | Ggh | gamma-glutamyl hydrolase | -2,05 | 6,12E-06 |
| 1429891_at | NM_029341 | Capsl | calcyphosine-like | -2,02 | 1,13E-04 |
| 1418735_at | NM_008475 | Krt4 | keratin 4 | -2,01 | 1,66E-04 |
| 1435853_at | NM_201360 | Cyp2d12 | cytochrome P450, family 2, subfamily d, polypeptide 12 | -1,99 | 3,57E-05 |
| 1449586_at | NM_019645 | Pkp1 | plakophilin 1 | -1,98 | 1,01E-03 |
| 1436590_at | NM_177741 | Ppp1r3b | protein phosphatase 1, regulatory (inhibitor) subunit 3B | -1,97 | 2,38E-04 |
| 1426566_s_at | NM_001034029 | Il17re | interleukin 17 receptor E | -1,97 | 9,06E-06 |
| 1449568_at | NM_031180 | Klb | klotho beta | -1,96 | 4,34E-03 |
| 1435761_at | NM_001082543 | BC100530 /// Stfa1 | cDNA sequence BC100530 /// stefin A1 | -1,95 | 2,48E-02 |
| 1428805_at | NM_029875 | Slc35e3 | solute carrier family 35, member E3 | -1,95 | 5,89E-07 |
| 1449670_x_at | NM_031999 | Gpr137b | G protein-coupled receptor 137B | -1,95 | 8,86E-03 |
| 1436223_at | NM_177290 | Itgb8 | integrin beta 8 | -1,94 | 8,07E-05 |
| 1429285_at | NM_027997 | Serpina9 | serine (or cysteine) peptidase inhibitor, clade A (alpha-1 antiproteinase, antitrypsin), member 9 | -1,92 | 1,15E-02 |
| 1424899_at | NM_144533 | Nmnat3 | nicotinamide nucleotide adenylyltransferase 3 | -1,92 | 6,18E-04 |
| 1441102_at | NM_011169 | Prlr | prolactin receptor | -1,92 | 1,49E-03 |
| 1448028_at | NM_001163847 | Tbc1d24 | TBC1 domain family, member 24 | -1,92 | 2,44E-04 |
| 1433615_at | NM_178789 | Tmem117 | transmembrane protein 117 | -1,91 | 5,23E-04 |
| 1444199_at | --- | --- | --- | -1,91 | 1,62E-05 |
| 1434657_at | NM_001081081 | Gls | glutaminase | -1,91 | 5,41E-05 |
| 1439255_s_at | NM_031999 | Gpr137b /// Gpr137b-ps | G protein-coupled receptor 137B /// G protein-coupled receptor 137B, pseudogene | -1,90 | 6,20E-06 |
| 1449161_at | NM_007902 | Edn2 | endothelin 2 | -1,90 | 1,42E-05 |
| 1455531_at | NM_001114662 | Mfsd4 | major facilitator superfamily domain containing 4 | -1,89 | 2,04E-06 |
| 1416710_at | NM_026239 | Tmem35 | transmembrane protein 35 | -1,88 | 3,57E-03 |
| 1458585_at | --- | --- | --- | -1,88 | 2,93E-05 |
| 1426917_s_at | NM_029022 | Scrn3 | secernin 3 | -1,87 | 6,47E-05 |
| 1421614_at | NM_011741 | Zan | zonadhesin | -1,86 | 2,28E-05 |
| 1437135_at | NM_001033669 | Fbxw10 | F-box and WD-40 domain protein 10 | -1,85 | 2,56E-03 |
| 1448813_at | NM_023383 | Aadac | arylacetamide deacetylase (esterase) | -1,84 | 1,74E-06 |
| 1442113_at | NM_001033304 | 5330417C22Rik | RIKEN cDNA 5330417C22 gene | -1,84 | 2,92E-06 |
| 1457619_at | NM_198171 | Ces2b | carboxyesterase 2B | -1,83 | 1,90E-03 |
| 1437874_s_at | NM_010422 | Hexb | hexosaminidase B | -1,82 | 4,59E-05 |
| 1425221_at | NM_207531 | Agr3 | anterior gradient homolog 3 (Xenopus laevis) | -1,81 | 8,74E-03 |
| 1439256_x_at | NR_003568 | Gpr137b-ps | G protein-coupled receptor 137B, pseudogene | -1,81 | 4,65E-05 |
| 1427263_at | NR_001463 | Xist | inactive X specific transcripts | -1,81 | 3,33E-06 |
| 1440870_at | NM_001177995 | Prdm16 | PR domain containing 16 | -1,81 | 1,38E-03 |
| 1448568_a_at | NM_001159593 | Slc20a1 | solute carrier family 20, member 1 | -1,81 | 3,35E-05 |
| 1418608_at | NM_027416 | Calml3 | calmodulin-like 3 | -1,79 | 1,33E-02 |
| 1450881_s_at | NM_031999 | Gpr137b | G protein-coupled receptor 137B | -1,79 | 2,43E-05 |
| 1450240_a_at | NM_031393 | Sytl1 | synaptotagmin-like 1 | -1,78 | 1,35E-04 |
| 1427512_a_at | NM_010680 | Lama3 | laminin, alpha 3 | -1,78 | 4,58E-04 |
| 1428812_at | NM_028491 | 1700040L02Rik | RIKEN cDNA 1700040L02 gene | -1,76 | 4,22E-05 |
| 1435387_at | NM_001033633 | Slc2a13 | solute carrier family 2 (facilitated glucose transporter), member 13 | -1,75 | 5,08E-04 |
| 1421221_at | NM_133217 | Bco2 | beta-carotene oxygenase 2 | -1,75 | 4,67E-05 |
| 1447173_at | XR_002334 | Lrrc31 | leucine rich repeat containing 31 | -1,74 | 2,94E-05 |
| 1434191_at | NM_178767 | Tmem195 | transmembrane protein 195 | -1,74 | 1,18E-03 |
| 1447885_x_at | NM_001111324 | Nedd9 | neural precursor cell expressed, developmentally down-regulated gene 9 | -1,74 | 4,60E-03 |
| 1436021_at | NM_001114662 | Mfsd4 | major facilitator superfamily domain containing 4 | -1,74 | 4,59E-07 |
| 1417522_at | NM_026346 | Fbxo32 | F-box protein 32 | -1,74 | 6,60E-05 |
| 1429775_a_at | NM_031999 | Gpr137b /// Gpr137b-ps | G protein-coupled receptor 137B /// G protein-coupled receptor 137B, pseudogene | -1,74 | 1,64E-05 |
| 1444602_at | NM_052976 | Ophn1 | oligophrenin 1 | -1,73 | 1,98E-05 |
| 1419523_at | NM_007819 | Cyp3a13 | cytochrome P450, family 3, subfamily a, polypeptide 13 | -1,73 | 2,75E-06 |
| 1428547_at | NM_011851 | Nt5e | 5' nucleotidase, ecto | -1,72 | 2,20E-04 |
| 1460606_at | NM_001163486 | Hsd17b13 | hydroxysteroid (17-beta) dehydrogenase 13 | -1,72 | 1,80E-04 |
| 1423439_at | NM_011044 | Pck1 | phosphoenolpyruvate carboxykinase 1, cytosolic | -1,72 | 4,49E-04 |
| 1419079_at | NM_011326 | Scnn1g | sodium channel, nonvoltage-gated 1 gamma | -1,71 | 2,61E-04 |
| 1440910_at | NM_001077354 | C77370 | expressed sequence C77370 | -1,70 | 6,59E-04 |
| 1460244_at | NM_133995 | Upb1 | ureidopropionase, beta | -1,69 | 1,28E-05 |
| 1419549_at | NM_007482 | Arg1 | arginase, liver | -1,68 | 1,01E-03 |
| 1440201_at | NM_001112798 | Slc8a1 | solute carrier family 8 (sodium/calcium exchanger), member 1 | -1,68 | 6,21E-04 |
| 1459889_at | NM_017474 | Clca3 | chloride channel calcium activated 3 | -1,68 | 8,52E-08 |
| 1424600_at | NM_001161621 | Abp1 | amiloride binding protein 1 (amine oxidase, copper-containing) | -1,67 | 1,29E-05 |
| 1425069_at | NM_145474 | Cyp2d34 | cytochrome P450, family 2, subfamily d, polypeptide 34 | -1,67 | 2,19E-05 |
| 1447891_at | --- | --- | --- | -1,66 | 1,16E-03 |
| 1417155_at | NM_008709 | Mycn | v-myc myelocytomatosis viral related oncogene, neuroblastoma derived (avian) | -1,66 | 1,09E-02 |
| 1447894_x_at | NM_172620 | Vps52 | Vacuolar protein sorting 52 (yeast) | -1,66 | 1,72E-04 |
| 1417686_at | NM_019516 | Lgals12 | lectin, galactose binding, soluble 12 | -1,65 | 2,65E-04 |
| 1418507_s_at | NM_001168655 | Socs2 | suppressor of cytokine signaling 2 | -1,65 | 1,66E-05 |
| 1423136_at | NM_010197 | Fgf1 | fibroblast growth factor 1 | -1,64 | 1,83E-05 |
| 1441341_at | NM_001160265 | Cyp2w1 | Cytochrome P450, family 2, subfamily w, polypeptide 1 | -1,64 | 1,40E-03 |
| 1419349_a_at | NM_010006 | Cyp2d9 | cytochrome P450, family 2, subfamily d, polypeptide 9 | -1,64 | 7,36E-04 |
| 1450767_at | NM_001111324 | Nedd9 | neural precursor cell expressed, developmentally down-regulated gene 9 | -1,63 | 1,71E-05 |
| 1437085_at | NM_178727 | D630039A03Rik | RIKEN cDNA D630039A03 gene | -1,63 | 6,84E-07 |
| 1439714_at | --- | --- | --- | -1,62 | 1,50E-03 |
| 1420683_at | NM_001168356 | Bnipl | BCL2/adenovirus E1B 19kD interacting protein like | -1,62 | 1,41E-03 |
| 1440745_at | NM_001177995 | Prdm16 | PR domain containing 16 | -1,62 | 3,24E-03 |
| 1434096_at | NM_001136260 | Slc4a4 | solute carrier family 4 (anion exchanger), member 4 | -1,62 | 2,22E-07 |
| 1435308_at | NM_010243 | Fut9 | fucosyltransferase 9 | -1,62 | 2,15E-05 |
| 1435665_at | NM_001167828 | Trim30d | tripartite motif-containing 30D | -1,61 | 1,32E-04 |
| 1425729_at | NM_001130193 | Best2 | bestrophin 2 | -1,61 | 3,74E-05 |
| 1438442_at | NM_025679 | Sike1 | suppressor of IKBKE 1 | -1,60 | 3,76E-05 |
| 1434099_at | NM_008904 | Ppargc1a | peroxisome proliferative activated receptor, gamma, coactivator 1 alpha | -1,60 | 1,40E-04 |
| 1422634_a_at | NM_020518 | Vsig2 | V-set and immunoglobulin domain containing 2 | -1,59 | 1,02E-05 |
| 1422818_at | NM_001111324 | Nedd9 | neural precursor cell expressed, developmentally down-regulated gene 9 | -1,59 | 6,92E-05 |
| 1458658_at | --- | --- | --- | -1,58 | 1,94E-03 |
| 1442290_at | NM_001037752 | Defb45 | defensin beta 45 | -1,57 | 2,35E-04 |
| 1448786_at | NM_025806 | Plbd1 | phospholipase B domain containing 1 | -1,57 | 6,24E-05 |
| 1455623_at | NM_001114662 | Mfsd4 | major facilitator superfamily domain containing 4 | -1,56 | 6,19E-03 |
| 1440411_at | --- | --- | --- | -1,56 | 1,47E-03 |
| 1435265_at | XR_105720 | LOC100504698 | hypothetical LOC100504698 | -1,55 | 1,33E-04 |
| 1421225_a_at | NM_001136260 | Slc4a4 | solute carrier family 4 (anion exchanger), member 4 | -1,55 | 3,25E-04 |
| 1426876_at | NM_178079 | Pm20d1 | peptidase M20 domain containing 1 | -1,55 | 6,64E-04 |
| 1450869_at | NM_010197 | Fgf1 | fibroblast growth factor 1 | -1,55 | 3,84E-05 |
| 1460336_at | NM_008904 | Ppargc1a | peroxisome proliferative activated receptor, gamma, coactivator 1 alpha | -1,54 | 2,49E-06 |
| 1422481_at | NM_008473 | Krt1 | keratin 1 | -1,53 | 4,13E-02 |
| 1416958_at | NM_011584 | Nr1d2 | nuclear receptor subfamily 1, group D, member 2 | -1,53 | 4,57E-04 |
| 1417590_at | NM_024264 | Cyp27a1 | cytochrome P450, family 27, subfamily a, polypeptide 1 | -1,53 | 4,80E-04 |
| 1433586_at | NM_178615 | Rgmb | RGM domain family, member B | -1,52 | 3,61E-04 |
| 1449625_at | --- | --- | --- | -1,52 | 1,09E-03 |
| 1423852_at | NM_145463 | Shisa2 | shisa homolog 2 (Xenopus laevis) | -1,52 | 1,56E-04 |
| 1438824_at | NM_001159593 | Slc20a1 | solute carrier family 20, member 1 | -1,52 | 7,71E-04 |
| 1417441_at | NM_013888 | Dnajc12 | DnaJ (Hsp40) homolog, subfamily C, member 12 | -1,51 | 3,95E-05 |
| 1450882_s_at | NM_031999 | Gpr137b /// Gpr137b-ps | G protein-coupled receptor 137B /// G protein-coupled receptor 137B, pseudogene | -1,51 | 3,04E-06 |
| 1439484_at | NM_001122759 | Pde7a | phosphodiesterase 7A | -1,51 | 1,04E-05 |
| 1456395_at | NM_008904 | Ppargc1a | peroxisome proliferative activated receptor, gamma, coactivator 1 alpha | -1,51 | 3,55E-05 |
| 1433674_a_at | NR_002896 | Snhg1 | small nucleolar RNA host gene (non-protein coding) 1 | -1,50 | 1,06E-04 |
| 1425002_at | NM_145373 | Sectm1a | secreted and transmembrane 1A | -1,49 | 3,82E-05 |
| 1418509_at | NM_007621 | Cbr2 | carbonyl reductase 2 | -1,49 | 9,32E-04 |
| 1450633_at | NM_020036 | Calm4 | calmodulin 4 | -1,49 | 1,20E-03 |
| 1440213_a_at | NR_026688 | 2010001M06Rik | RIKEN cDNA 2010001M06 gene | -1,49 | 3,25E-06 |
| 1430637_at | --- | 2210016H18Rik | RIKEN cDNA 2210016H18 gene | -1,48 | 1,22E-04 |
| 1438532_at | NM_001024720 | Hmcn1 | hemicentin 1 | -1,48 | 1,70E-03 |
| 1436520_at | XM_003085583 | Ahnak2 | AHNAK nucleoprotein 2 | -1,48 | 9,84E-06 |
| 1454604_s_at | NM_173007 | Tspan12 | tetraspanin 12 | -1,47 | 3,72E-04 |
| 1419479_at | NM_026907 | Sectm1b | secreted and transmembrane 1B | -1,47 | 2,36E-04 |
| 1417879_at | NM_025424 | Nenf | neuron derived neurotrophic factor | -1,46 | 3,31E-05 |
| 1455445_at | NM_019820 | Cbln3 | cerebellin 3 precursor protein | -1,46 | 1,68E-03 |
| 1437132_x_at | NM_001111324 | Nedd9 | neural precursor cell expressed, developmentally down-regulated gene 9 | -1,46 | 3,77E-04 |
| 1450933_at | NM_001122759 | Pde7a | phosphodiesterase 7A | -1,46 | 7,75E-05 |
| 1434526_at | NM_001001804 | Ephx4 | epoxide hydrolase 4 | -1,46 | 8,00E-04 |
| 1435691_at | NM_177351 | Agphd1 | aminoglycoside phosphotransferase domain containing 1 | -1,46 | 6,84E-05 |
| 1418138_at | NM_016771 | Sult1d1 | sulfotransferase family 1D, member 1 | -1,45 | 2,17E-04 |
| 1441975_at | NM_019807 | Acpp | acid phosphatase, prostate | -1,45 | 8,23E-06 |
| 1455007_s_at | NM_173866 | Gpt2 | glutamic pyruvate transaminase (alanine aminotransferase) 2 | -1,45 | 7,10E-06 |
| 1457883_at | NM_001034867 | Pm20d2 | peptidase M20 domain containing 2 | -1,44 | 1,77E-03 |
| 1442067_at | --- | --- | --- | -1,44 | 7,58E-05 |
| 1423851_a_at | NM_145463 | Shisa2 | shisa homolog 2 (Xenopus laevis) | -1,43 | 2,15E-03 |
| 1425182_x_at | NM_010114 | Klk1b22 /// Klk1b9 | kallikrein 1-related peptidase b22 /// kallikrein 1-related peptidase b9 | -1,42 | 9,60E-04 |
| 1455010_at | NM_001081005 | 1500012F01Rik | RIKEN cDNA 1500012F01 gene | -1,42 | 1,12E-08 |
| 1434100_x_at | NM_008904 | Ppargc1a | peroxisome proliferative activated receptor, gamma, coactivator 1 alpha | -1,41 | 1,04E-03 |
| 1422293_a_at | NM_001142731 | Kctd1 | potassium channel tetramerisation domain containing 1 | -1,41 | 9,13E-05 |
| 1424359_at | NM_153122 | Oplah | 5-oxoprolinase (ATP-hydrolysing) | -1,41 | 5,43E-04 |
| 1417962_s_at | NM_001048147 | Ghr | growth hormone receptor | -1,41 | 1,95E-04 |
| 1454883_at | NM_001168274 | Gsdmc2 | gasdermin C2 | -1,41 | 5,27E-05 |
| 1419759_at | NM_011076 | Abcb1a | ATP-binding cassette, sub-family B (MDR/TAP), member 1A | -1,41 | 2,40E-05 |
| 1449109_at | NM_001168655 | Socs2 | suppressor of cytokine signaling 2 | -1,40 | 3,10E-05 |
| 1448747_at | NM_026346 | Fbxo32 | F-box protein 32 | -1,40 | 2,27E-04 |
| 1419010_x_at | NM_008456 | Klk1b5 | kallikrein 1-related peptidase b5 | -1,40 | 4,16E-04 |
| 1423935_x_at | NM_016958 | Krt14 | keratin 14 | -1,40 | 1,13E-04 |
| 1416179_a_at | NM_001104616 | Rdx | radixin | -1,40 | 5,92E-04 |
| 1441622_at | NR_015373 | Gm5485 | predicted gene 5485 | -1,40 | 3,34E-05 |
| 1425010_at | NM_144546 | Zfp119a | zinc finger protein 119a | -1,40 | 1,18E-04 |
| 1433675_at | NR_002896 | Snhg1 | small nucleolar RNA host gene (non-protein coding) 1 | -1,39 | 1,23E-04 |
| 1442325_at | NM_001163847 | Tbc1d24 | TBC1 domain family, member 24 | -1,39 | 6,12E-03 |
| 1425893_a_at | NM_010210 | Fhit | fragile histidine triad gene | -1,39 | 3,03E-06 |
| 1433914_at | NM_001013770 | Lipo1 | lipase, member O1 | -1,38 | 2,95E-04 |
| 1455712_at | NM_178218 | Hist3h2a | histone cluster 3, H2a | -1,38 | 1,56E-04 |
| 1438751_at | NM_001033286 | Slc30a10 | solute carrier family 30, member 10 | -1,38 | 3,87E-05 |
| 1426464_at | NM_145434 | Nr1d1 | nuclear receptor subfamily 1, group D, member 1 | -1,38 | 4,19E-03 |
| 1437751_at | NM_008904 | Ppargc1a | peroxisome proliferative activated receptor, gamma, coactivator 1 alpha | -1,37 | 3,08E-05 |
| 1438982_s_at | NM_029798 | Flywch2 | FLYWCH family member 2 | -1,37 | 6,73E-05 |
| 1427195_at | NM_007885 | Slc26a2 | solute carrier family 26 (sulfate transporter), member 2 | -1,37 | 4,12E-07 |
| 1423314_s_at | NM_001122759 | Pde7a | phosphodiesterase 7A | -1,37 | 2,01E-03 |
| 1439617_s_at | NM_011044 | Pck1 | phosphoenolpyruvate carboxykinase 1, cytosolic | -1,36 | 3,15E-04 |
| 1425746_at | NM_030021 | D730039F16Rik | RIKEN cDNA D730039F16 gene | -1,36 | 7,29E-05 |
| 1419072_at | NM_026672 | Gstm7 | glutathione S-transferase, mu 7 | -1,36 | 2,02E-04 |
| 1427537_at | NM_144848 | Eppk1 | epiplakin 1 | -1,36 | 3,68E-04 |
| 1454748_at | NM_172607 | Naprt1 | nicotinate phosphoribosyltransferase domain containing 1 | -1,36 | 5,80E-06 |
| 1416715_at | NM_001160012 | Gjb3 | gap junction protein, beta 3 | -1,36 | 9,32E-04 |
| 1417988_at | NM_009049 | Resp18 | regulated endocrine-specific protein 18 | -1,36 | 9,56E-06 |
| 1429727_at | NM_025807 | Slc16a9 | solute carrier family 16 (monocarboxylic acid transporters), member 9 | -1,35 | 1,97E-04 |
| 1455591_at | NM_028326 | Zfp618 | zinc fingerprotein 618 | -1,35 | 1,28E-04 |
| 1457321_at | XM_001474094 | D130037M23Rik | RIKEN cDNA D130037M23 gene | -1,35 | 1,23E-04 |
| 1452071_at | NM_001136260 | Slc4a4 | solute carrier family 4 (anion exchanger), member 4 | -1,35 | 8,05E-04 |
| 1427262_at | NR_001463 | Xist | inactive X specific transcripts | -1,35 | 6,24E-03 |
| 1423865_at | NM_001159633 | Slc44a1 | solute carrier family 44, member 1 | -1,35 | 1,49E-04 |
| 1450863_a_at | NM_001111051 | Dclk1 | doublecortin-like kinase 1 | -1,35 | 1,30E-04 |
| 1425201_a_at | NM_026601 | Hyi | hydroxypyruvate isomerase homolog (E. coli) | -1,34 | 4,77E-04 |
| 1448265_x_at | NM_007962 | Mpzl2 | myelin protein zero-like 2 | -1,34 | 4,55E-07 |
| 1451547_at | NM_027391 | Iyd | iodotyrosine deiodinase | -1,34 | 2,54E-05 |
| 1444589_at | XM_003085147 | Gm4944 | predicted gene 4944 | -1,34 | 4,90E-05 |
| 1419033_at | NM_133729 | 2610018G03Rik | RIKEN cDNA 2610018G03 gene | -1,34 | 1,03E-03 |
| 1439399_a_at | NR_002896 | Snhg1 | small nucleolar RNA host gene (non-protein coding) 1 | -1,33 | 7,85E-04 |
| 1418780_at | NM_018887 | Cyp39a1 | cytochrome P450, family 39, subfamily a, polypeptide 1 | -1,33 | 3,94E-04 |
| 1438470_at | NM_001168655 | Socs2 | suppressor of cytokine signaling 2 | -1,33 | 9,92E-04 |
| 1449456_a_at | NM_010780 | Cma1 | chymase 1, mast cell | -1,33 | 4,62E-04 |
| 1435860_at | NM_001177621 | Slc5a6 | solute carrier family 5 (sodium-dependent vitamin transporter), member 6 | -1,33 | 1,85E-04 |
| 1453239_a_at | NM_024204 | Ankrd22 | ankyrin repeat domain 22 | -1,33 | 1,21E-02 |
| 1423298_at | NM_001164099 | Add3 | adducin 3 (gamma) | -1,33 | 5,35E-04 |
| 1451839_a_at | NM_001122759 | Pde7a | phosphodiesterase 7A | -1,32 | 8,23E-03 |
| 1435048_at | NR_027236 | AI854703 | expressed sequence AI854703 | -1,32 | 2,08E-05 |
| 1421145_at | NM_007885 | Slc26a2 | solute carrier family 26 (sulfate transporter), member 2 | -1,31 | 1,36E-04 |
| 1434340_at | NM_197979 | Uqcr10 | ubiquinol-cytochrome c reductase, complex III subunit X | -1,31 | 1,10E-05 |
| 1438666_at | NM_178886 | Ldlrad3 | low density lipoprotein receptor class A domain containing 3 | -1,31 | 4,50E-03 |
| 1418037_at | NM_007576 | C4bp | complement component 4 binding protein | -1,31 | 1,24E-05 |
| 1435435_at | NM_080285 | Cttnbp2 | cortactin binding protein 2 | -1,31 | 8,12E-05 |
| 1435198_at | XM_001480818 | Gm5817 | predicted gene 5817 | -1,31 | 2,34E-05 |
| 1419108_at | NM_052976 | Ophn1 | oligophrenin 1 | -1,31 | 3,04E-03 |
| 1430688_s_at | NM_030021 | D730039F16Rik | RIKEN cDNA D730039F16 gene | -1,31 | 2,94E-04 |
| 1444242_at | NM_033314 | Slco2a1 | Solute carrier organic anion transporter family, member 2a1 | -1,31 | 5,91E-04 |
| 1420249_s_at | NM_009139 | Ccl6 | chemokine (C-C motif) ligand 6 | -1,31 | 6,18E-05 |
| 1429265_a_at | NM_021540 | Rnf130 | ring finger protein 130 | -1,30 | 3,12E-04 |
| 1424716_at | NM_026159 | Retsat | retinol saturase (all trans retinol 13,14 reductase) | -1,30 | 2,10E-04 |
| 1451229_at | NM_144919 | Hdac11 | histone deacetylase 11 | -1,30 | 2,17E-05 |
| 1460386_a_at | NM_009199 | Slc1a1 | solute carrier family 1 (neuronal/epithelial high affinity glutamate transporter, system Xag), member 1 | -1,30 | 3,98E-05 |
| 1448246_at | NM_008228 | Hdac1 | histone deacetylase 1 | -1,30 | 2,09E-05 |
| 1434497_at | NR_015459 | 4933431E20Rik | RIKEN cDNA 4933431E20 gene | -1,30 | 2,50E-03 |
| 1438041_at | NM_001122759 | Pde7a | phosphodiesterase 7A | -1,30 | 4,09E-04 |
| 1450770_at | NM_026142 | 3632451O06Rik | RIKEN cDNA 3632451O06 gene | -1,30 | 1,85E-03 |
| 1424618_at | NM_008277 | Hpd | 4-hydroxyphenylpyruvic acid dioxygenase | -1,29 | 1,82E-06 |
| 1418203_at | NM_021451 | Pmaip1 | phorbol-12-myristate-13-acetate-induced protein 1 | -1,29 | 1,22E-05 |
| 1421471_at | NM_010934 | Npy1r | neuropeptide Y receptor Y1 | -1,29 | 3,39E-04 |
| 1450813_a_at | NM_001112702 | Tnni1 | troponin I, skeletal, slow 1 | -1,29 | 5,28E-04 |
| 1448236_at | NM_001104616 | Rdx | radixin | -1,29 | 1,58E-04 |
| 1418219_at | NM_008357 | Il15 | interleukin 15 | -1,28 | 8,40E-06 |
| 1449648_s_at | NM_009085 | Polr1c | polymerase (RNA) I polypeptide C | -1,28 | 2,94E-04 |
| 1455425_at | NM_027384 | Tet1 | tet oncogene 1 | -1,28 | 1,38E-03 |
| 1454635_at | NM_015822 | Fbxl3 | F-box and leucine-rich repeat protein 3 | -1,28 | 2,07E-04 |
| 1452799_at | NM_001113412 | Fggy | FGGY carbohydrate kinase domain containing | -1,28 | 1,35E-05 |
| 1444611_at | --- | --- | --- | -1,28 | 5,21E-04 |
| 1438385_s_at | NM_173866 | Gpt2 | glutamic pyruvate transaminase (alanine aminotransferase) 2 | -1,28 | 3,15E-03 |
| 1447936_at | NR_030738 | 2410006H16Rik | RIKEN cDNA 2410006H16 gene | -1,28 | 1,00E-04 |
| 1435069_at | NR_015455 | BC064078 | cDNA sequence BC064078 | -1,27 | 1,60E-02 |
| 1429173_at | NM_001172154 | Dnase1l1 | deoxyribonuclease 1-like 1 | -1,27 | 4,07E-04 |
| 1436534_at | NM_013835 | Trove2 | TROVE domain family, member 2 | -1,27 | 3,08E-05 |
| 1443882_at | NM_007885 | Slc26a2 | solute carrier family 26 (sulfate transporter), member 2 | -1,27 | 9,49E-06 |
| 1416832_at | NM_001135149 | Slc39a8 | solute carrier family 39 (metal ion transporter), member 8 | -1,27 | 3,83E-05 |
| 1444402_at | NM_001162921 | Zc3h12c | zinc finger CCCH type containing 12C | -1,27 | 3,25E-03 |
| 1429550_at | NM_028093 | Entpd8 | ectonucleoside triphosphate diphosphohydrolase 8 | -1,26 | 8,34E-04 |
| 1437397_at | NM_011169 | Prlr | prolactin receptor | -1,26 | 3,57E-05 |
| 1439183_at | NM_175731 | Acer1 | alkaline ceramidase 1 | -1,26 | 5,06E-04 |
| 1440799_s_at | NM_145519 | Farp2 | FERM, RhoGEF and pleckstrin domain protein 2 | -1,25 | 3,67E-04 |
| 1417150_at | NM_010484 | Slc6a4 | solute carrier family 6 (neurotransmitter transporter, serotonin), member 4 | -1,25 | 2,24E-03 |
| 1453511_at | NM_001159940 | 2310007B03Rik | RIKEN cDNA 2310007B03 gene | -1,25 | 1,05E-04 |
| 1435672_at | --- | 3830612M24 | hypothetical protein 3830612M24 | -1,25 | 4,62E-04 |
| 1449292_at | NM_009826 | Rb1cc1 | RB1-inducible coiled-coil 1 | -1,25 | 9,55E-04 |
| 1453230_at | NM_178384 | Zfp74 | zinc finger protein 74 | -1,25 | 8,63E-05 |
| 1419075_s_at | NM_009117 | Saa1 | serum amyloid A 1 | -1,25 | 1,10E-06 |
| 1416854_at | NM_011402 | Slc34a2 | solute carrier family 34 (sodium phosphate), member 2 | -1,25 | 2,78E-05 |
| 1432336_at | NR_033600 | 5033404E19Rik | RIKEN cDNA 5033404E19 gene | -1,24 | 1,12E-03 |
| 1456640_at | NM_001146299 | Sh3rf2 | SH3 domain containing ring finger 2 | -1,24 | 5,24E-05 |
| 1418174_at | NM_016974 | Dbp | D site albumin promoter binding protein | -1,24 | 1,30E-05 |
| 1418149_at | NM_007693 | Chga | chromogranin A | -1,24 | 1,30E-05 |
| 1423405_at | NM_080639 | Timp4 | tissue inhibitor of metalloproteinase 4 | -1,24 | 1,70E-05 |
| 1427056_at | NM_001024139 | Adamts15 | a disintegrin-like and metallopeptidase (reprolysin type) with thrombospondin type 1 motif, 15 | -1,24 | 2,00E-05 |
| 1439526_at | --- | --- | --- | -1,24 | 3,23E-04 |
| 1417279_at | NM_010585 | Itpr1 | inositol 1,4,5-triphosphate receptor 1 | -1,23 | 2,73E-05 |
| 1444262_at | NM_001162998 | 1110017F19Rik | RIKEN cDNA 1110017F19 gene | -1,23 | 8,44E-06 |
| 1460203_at | NM_010585 | Itpr1 | inositol 1,4,5-triphosphate receptor 1 | -1,23 | 7,04E-05 |
| 1417066_at | NM_001163290 | Adck3 | aarF domain containing kinase 3 | -1,23 | 2,09E-06 |
| 1451704_at | NM_001009952 | Abpd | androgen binding protein delta | -1,23 | 1,81E-02 |
| 1459840_s_at | NM_025455 | Ccdc28b | coiled coil domain containing 28B | -1,23 | 1,09E-04 |
| 1438452_at | NM_028757 | Nebl | nebulette | -1,23 | 1,36E-03 |
| 1417455_at | NM_009368 | Tgfb3 | transforming growth factor, beta 3 | -1,23 | 2,25E-03 |
| 1438211_s_at | NM_016974 | Dbp | D site albumin promoter binding protein | -1,23 | 5,52E-05 |
| 1449459_s_at | NM_080857 | Asb13 | ankyrin repeat and SOCS box-containing 13 | -1,23 | 8,90E-05 |
| 1434906_at | XR_105106 | 0610005C13Rik | RIKEN cDNA 0610005C13 gene | -1,23 | 4,86E-03 |
| 1453393_a_at | NM_011998 | Chst4 | carbohydrate (chondroitin 6/keratan) sulfotransferase 4 | -1,22 | 2,65E-06 |
| 1442263_at | NM_153171 | Rgs13 | regulator of G-protein signaling 13 | -1,22 | 6,75E-04 |
| 1449972_s_at | NM_001005358 | BC018101 /// Zfp97 | cDNA sequence BC018101 /// zinc finger protein 97 | -1,22 | 7,66E-05 |
| 1416236_a_at | NM_007962 | Mpzl2 | myelin protein zero-like 2 | -1,22 | 1,09E-06 |
| 1436533_at | NM_013835 | Trove2 | TROVE domain family, member 2 | -1,22 | 7,62E-05 |
| 1433453_a_at | NM_178890 | Abtb2 | ankyrin repeat and BTB (POZ) domain containing 2 | -1,22 | 1,50E-03 |
| 1423297_at | NM_001164099 | Add3 | adducin 3 (gamma) | -1,21 | 7,11E-04 |
| 1425057_at | NM_026701 | Pbld | phenazine biosynthesis-like protein domain containing | -1,21 | 1,78E-03 |
| 1434725_at | NM_001172107 | Gramd1c | GRAM domain containing 1C | -1,21 | 2,19E-04 |
| 1434477_at | NM_001033432 | Heca | headcase homolog (Drosophila) | -1,21 | 5,45E-05 |
| 1426432_a_at | NM_001136260 | Slc4a4 | solute carrier family 4 (anion exchanger), member 4 | -1,20 | 1,03E-04 |
| 1453289_at | NM_153177 | Eif2c4 | eukaryotic translation initiation factor 2C, 4 | -1,20 | 2,95E-04 |
| 1437798_at | --- | 6720422M22Rik | RIKEN cDNA 6720422M22 gene | -1,20 | 3,19E-03 |
| 1458367_at | NM_001033280 | Gm94 | predicted gene 94 | -1,19 | 5,14E-03 |
| 1427112_at | NM_027192 | Ttl | tubulin tyrosine ligase | -1,19 | 7,05E-04 |
| 1440285_at | NM_181595 | Ppp1r9a | protein phosphatase 1, regulatory (inhibitor) subunit 9A | -1,19 | 2,35E-05 |
| 1422595_s_at | NM_025679 | Sike1 | suppressor of IKBKE 1 | -1,19 | 7,22E-05 |
| 1451501_a_at | NM_001048147 | Ghr | growth hormone receptor | -1,19 | 3,21E-04 |
| 1452583_s_at | NM_176963 | Galm | galactose mutarotase | -1,19 | 8,90E-06 |
| 1421489_a_at | NM_001168590 | 2010106E10Rik | RIKEN cDNA 2010106E10 gene | -1,19 | 1,61E-03 |
| 1456981_at | NM_172476 | Tmc7 | transmembrane channel-like gene family 7 | -1,19 | 2,23E-04 |
| 1429308_at | NM_001177995 | Prdm16 | PR domain containing 16 | -1,19 | 2,73E-03 |
| 1436556_at | NM_175408 | Tmem139 | transmembrane protein 139 | -1,18 | 3,78E-04 |
| 1448556_at | NM_011169 | Prlr | prolactin receptor | -1,18 | 3,17E-03 |
| 1423164_at | NM_031999 | Gpr137b /// Gpr137b-ps | G protein-coupled receptor 137B /// G protein-coupled receptor 137B, pseudogene | -1,18 | 3,28E-06 |
| 1460242_at | NM_010016 | Cd55 | CD55 antigen | -1,18 | 7,94E-04 |
| 1427982_s_at | NM_001005510 | Syne2 | synaptic nuclear envelope 2 | -1,18 | 9,47E-07 |
| 1453092_at | NM_028798 | Crct1 | cysteine-rich C-terminal 1 | -1,17 | 2,24E-03 |
| 1460017_at | NM_001008427 | Gm5595 | predicted gene 5595 | -1,17 | 4,58E-03 |
| 1451074_at | NM_001113413 | Rnf13 | ring finger protein 13 | -1,17 | 1,29E-05 |
| 1448973_at | NM_016771 | Sult1d1 | sulfotransferase family 1D, member 1 | -1,17 | 6,61E-04 |
| 1428972_at | NM_025329 | Tctex1d2 | Tctex1 domain containing 2 | -1,17 | 1,74E-03 |
| 1450222_x_at | NM_010915 | Klk1b4 | kallikrein 1-related pepidase b4 | -1,17 | 1,25E-03 |
| 1425147_at | NM_001163518 | 2410075B13Rik | RIKEN cDNA 2410075B13 gene | -1,17 | 2,17E-03 |
| 1417611_at | NM_019432 | Tmem37 | transmembrane protein 37 | -1,16 | 1,71E-06 |
| 1448986_x_at | NM_010062 | Dnase2a | deoxyribonuclease II alpha | -1,16 | 1,84E-03 |
| 1427942_at | NM_146240 | Rassf9 | Ras association (RalGDS/AF-6) domain family (N-terminal) member 9 | -1,16 | 8,11E-03 |
| 1417689_a_at | NM_001164557 | Pdzk1ip1 | PDZK1 interacting protein 1 | -1,16 | 3,25E-04 |
| 1459897_a_at | NM_001083903 | Sbsn | suprabasin | -1,16 | 1,29E-04 |
| 1435115_at | NM_027402 | Fndc5 | fibronectin type III domain containing 5 | -1,16 | 3,63E-04 |
| 1457269_at | NM_001080776 | Gm1123 | predicted gene 1123 | -1,16 | 1,35E-04 |
| 1455843_at | NM_010242 | Fut4 | fucosyltransferase 4 | -1,15 | 8,51E-06 |
| 1427981_a_at | NM_144942 | Csad | cysteine sulfinic acid decarboxylase | -1,15 | 4,89E-04 |
| 1424175_at | NM_017376 | Tef | thyrotroph embryonic factor | -1,15 | 4,81E-04 |
| 1427063_at | NM_001033304 | 5330417C22Rik | RIKEN cDNA 5330417C22 gene | -1,15 | 2,07E-04 |
| 1435641_at | NM_173870 | Mgat4a | mannoside acetylglucosaminyltransferase 4, isoenzyme A | -1,15 | 4,57E-04 |
| 1418606_at | NM_013554 | Hoxd10 | homeobox D10 | -1,15 | 1,16E-03 |
| 1455549_at | NM_175465 | Sestd1 | SEC14 and spectrin domains 1 | -1,15 | 7,04E-06 |
| 1430211_at | XM_128114 | 4930415O20Rik | RIKEN cDNA 4930415O20 gene | -1,15 | 7,39E-05 |
| 1417481_at | NM_001168392 | Ramp1 | receptor (calcitonin) activity modifying protein 1 | -1,15 | 3,93E-05 |
| 1438026_at | NM_001004190 | Zfp560 | zinc finger protein 560 | -1,15 | 1,55E-03 |
| 1432355_at | XM_001476411 | 2210039B01Rik | RIKEN cDNA 2210039B01 gene | -1,15 | 2,60E-04 |
| 1440928_at | NM_001033385 | D630037F22Rik | RIKEN cDNA D630037F22 gene | -1,14 | 4,24E-04 |
| 1456169_at | NM_001164525 | Tstd1 | thiosulfate sulfurtransferase (rhodanese)-like domain containing 1 | -1,14 | 7,46E-05 |
| 1440922_at | XR_105147 | 9130208D14Rik | RIKEN cDNA 9130208D14 gene | -1,14 | 4,98E-06 |
| 1422552_at | NM_023396 | Rprm | reprimo, TP53 dependent G2 arrest mediator candidate | -1,14 | 1,65E-04 |
| 1428682_at | NM_178404 | Zc3h6 | zinc finger CCCH type containing 6 | -1,14 | 3,62E-04 |
| 1447252_s_at | NM_008585 | Mep1a | meprin 1 alpha | -1,14 | 1,71E-03 |
| 1440339_at | NM_008813 | Enpp1 | ectonucleotide pyrophosphatase/phosphodiesterase 1 | -1,14 | 2,86E-05 |
| 1460127_at | NM_013920 | Hnf4g | hepatocyte nuclear factor 4, gamma | -1,14 | 7,88E-03 |
| 1458040_at | --- | D7Wsu130e | DNA segment, Chr 7, Wayne State University 130, expressed | -1,14 | 2,52E-03 |
| 1436030_at | NM_198037 | Cachd1 | cache domain containing 1 | -1,13 | 1,59E-05 |
| 1439614_at | --- | --- | --- | -1,13 | 2,71E-03 |
| 1419039_at | NM_001163472 | Cyp2d22 | cytochrome P450, family 2, subfamily d, polypeptide 22 | -1,13 | 1,14E-04 |
| 1437361_at | NM_001105561 | Gm11545 | predicted gene 11545 | -1,13 | 5,09E-05 |
| 1451490_at | NM_146106 | Lyplal1 | lysophospholipase-like 1 | -1,13 | 1,75E-04 |
| 1456097_a_at | NM_026348 | Itgb3bp | integrin beta 3 binding protein (beta3-endonexin) | -1,13 | 5,86E-04 |
| 1434062_at | NM_001038621 | Rabgap1l | RAB GTPase activating protein 1-like | -1,13 | 9,48E-10 |
| 1431800_at | NM_027906 | 1300010F03Rik | RIKEN cDNA 1300010F03 gene | -1,12 | 3,40E-03 |
| 1455700_at | NM_028832 | Mterfd3 | MTERF domain containing 3 | -1,12 | 1,37E-03 |
| 1455477_s_at | NM_001164557 | Pdzk1ip1 | PDZK1 interacting protein 1 | -1,12 | 1,00E-07 |
| 1417965_at | NM_133942 | Plekha1 | pleckstrin homology domain containing, family A (phosphoinositide binding specific) member 1 | -1,12 | 7,28E-05 |
| 1429115_at | XR_106443 | 2010003O02Rik /// LOC100504309 | RIKEN cDNA 2010003O02 gene /// hypothetical LOC100504309 | -1,12 | 5,60E-05 |
| 1450734_at | NM_001159986 | Sec16b | SEC16 homolog B (S. cerevisiae) | -1,12 | 3,71E-05 |
| 1420772_a_at | NM_001077364 | Tsc22d3 | TSC22 domain family, member 3 | -1,11 | 7,08E-06 |
| 1436458_at | NM_018744 | Sema6a | sema domain, transmembrane domain (TM), and cytoplasmic domain, (semaphorin) 6A | -1,11 | 2,76E-03 |
| 1417635_at | NM_011449 | Spa17 | sperm autoantigenic protein 17 | -1,11 | 9,30E-04 |
| 1455220_at | NM_177603 | Frat2 | frequently rearranged in advanced T-cell lymphomas 2 | -1,11 | 8,72E-06 |
| 1440215_at | NM_001171801 | Gm11818 | predicted gene 11818 | -1,11 | 5,15E-06 |
| 1429833_at | NM_027366 | Ly6g6e | lymphocyte antigen 6 complex, locus G6E | -1,11 | 3,39E-03 |
| 1419478_at | NM_026907 | Sectm1b | secreted and transmembrane 1B | -1,11 | 1,73E-06 |
| 1438701_at | NM_001112796 | Bicd1 | bicaudal D homolog 1 (Drosophila) | -1,11 | 1,44E-03 |
| 1421852_at | NM_021542 | Kcnk5 | potassium channel, subfamily K, member 5 | -1,11 | 8,75E-05 |
| 1443671_x_at | NM_001013022 | Odf3b | outer dense fiber of sperm tails 3B | -1,11 | 1,12E-03 |
| 1416586_at | NM_001001792 | Zfp239 | zinc finger protein 239 | -1,11 | 9,18E-06 |
| 1429096_at | --- | 2810455D13Rik | RIKEN cDNA 2810455D13 gene | -1,11 | 7,81E-05 |
| 1430640_a_at | NM_011158 | Prkar2b | protein kinase, cAMP dependent regulatory, type II beta | -1,11 | 1,00E-02 |
| 1416180_a_at | NM_001104616 | Rdx | radixin | -1,10 | 1,01E-03 |
| 1418989_at | NM_007799 | Ctse | cathepsin E | -1,10 | 1,69E-05 |
| 1458218_s_at | NM_001122759 | Pde7a | phosphodiesterase 7A | -1,10 | 4,46E-06 |
| 1453016_at | NM_028394 | Hspb11 | heat shock protein family B (small), member 11 | -1,10 | 4,30E-06 |
| 1454803_a_at | NM_144919 | Hdac11 | histone deacetylase 11 | -1,10 | 9,89E-04 |
| 1424715_at | NM_026159 | Retsat | retinol saturase (all trans retinol 13,14 reductase) | -1,10 | 8,45E-04 |
| 1450505_a_at | NM_001034851 | Fam134b | family with sequence similarity 134, member B | -1,10 | 2,31E-05 |
| 1415685_at | NM_133767 | Mtif2 | mitochondrial translational initiation factor 2 | -1,10 | 3,32E-05 |
| 1439630_x_at | NM_001083903 | Sbsn | suprabasin | -1,10 | 8,03E-04 |
| 1455342_at | NM_181348 | Prune2 | prune homolog 2 (Drosophila) | -1,10 | 4,19E-03 |
| 1436190_at | NM_028326 | Zfp618 | zinc fingerprotein 618 | -1,10 | 2,11E-04 |
| 1443100_at | --- | --- | --- | -1,10 | 3,86E-03 |
| 1436549_a_at | NM_001039129 | Hnrnpa1 | heterogeneous nuclear ribonucleoprotein A1 | -1,10 | 2,87E-03 |
| 1454768_at | NM_201531 | Kcnf1 | potassium voltage-gated channel, subfamily F, member 1 | -1,10 | 6,02E-05 |
| 1452619_a_at | NM_178630 | Agbl3 | ATP/GTP binding protein-like 3 | -1,09 | 4,75E-05 |
| 1445815_at | NM_008058 | Fzd8 | frizzled homolog 8 (Drosophila) | -1,09 | 1,23E-05 |
| 1435601_at | NM_001122594 | Phlpp2 | PH domain and leucine rich repeat protein phosphatase 2 | -1,09 | 3,91E-05 |
| 1422644_at | NM_015825 | Sh3bgr | SH3-binding domain glutamic acid-rich protein | -1,09 | 1,97E-04 |
| 1458820_at | NM_001004190 | Zfp560 | zinc finger protein 560 | -1,09 | 1,48E-02 |
| 1456495_s_at | NM_145525 | Osbpl6 | oxysterol binding protein-like 6 | -1,09 | 8,18E-05 |
| 1419401_at | NM_080857 | Asb13 | ankyrin repeat and SOCS box-containing 13 | -1,09 | 3,50E-03 |
| 1436535_at | NM_013835 | Trove2 | TROVE domain family, member 2 | -1,09 | 1,83E-03 |
| 1452771_s_at | NM_001033606 | Acsl3 | acyl-CoA synthetase long-chain family member 3 | -1,09 | 8,15E-06 |
| 1439994_at | NM_001145433 | 1810013D10Rik | RIKEN cDNA 1810013D10 gene | -1,09 | 3,27E-03 |
| 1453135_at | NM_027402 | Fndc5 | fibronectin type III domain containing 5 | -1,08 | 2,30E-04 |
| 1427945_at | NM_170778 | Dpyd | dihydropyrimidine dehydrogenase | -1,08 | 2,42E-04 |
| 1447340_at | --- | --- | --- | -1,08 | 1,34E-03 |
| 1423433_at | NM_013835 | Trove2 | TROVE domain family, member 2 | -1,08 | 2,95E-06 |
| 1424959_at | NM_027211 | Anxa13 | annexin A13 | -1,08 | 5,37E-05 |
| 1420578_at | NM_001160420 | Optc | opticin | -1,08 | 8,80E-04 |
| 1454159_a_at | NM_008342 | Igfbp2 | insulin-like growth factor binding protein 2 | -1,08 | 1,74E-04 |
| 1428915_at | NM_178848 | Sirt5 | sirtuin 5 (silent mating type information regulation 2 homolog) 5 (S. cerevisiae) | -1,08 | 1,06E-04 |
| 1441342_at | NM_001159543 | Dpp4 | dipeptidylpeptidase 4 | -1,08 | 2,44E-03 |
| 1439887_at | NM_001160368 | Rnf152 | ring finger protein 152 | -1,08 | 3,65E-05 |
| 1434542_at | NM_173866 | Gpt2 | glutamic pyruvate transaminase (alanine aminotransferase) 2 | -1,08 | 1,16E-03 |
| 1416203_at | NM_007472 | Aqp1 | aquaporin 1 | -1,08 | 2,97E-06 |
| 1437208_at | NM_001024910 | sept10 | septin 10 | -1,08 | 3,44E-05 |
| 1443365_at | NM_008313 | Htr4 | 5 hydroxytryptamine (serotonin) receptor 4 | -1,08 | 6,60E-03 |
| 1417520_at | NM_010903 | Nfe2l3 | nuclear factor, erythroid derived 2, like 3 | -1,08 | 1,71E-04 |
| 1435866_s_at | NM_178218 | Hist3h2a | histone cluster 3, H2a | -1,07 | 6,76E-05 |
| 1418955_at | NM_009567 | Zfp93 | zinc finger protein 93 | -1,07 | 5,56E-03 |
| 1434446_at | NM_010568 | Insr | insulin receptor | -1,07 | 1,22E-05 |
| 1457044_at | NM_001163136 | Macc1 | metastasis associated in colon cancer 1 | -1,07 | 7,99E-04 |
| 1439531_at | NM_177856 | E130311K13Rik | RIKEN cDNA E130311K13 gene | -1,07 | 5,36E-04 |
| 1454959_s_at | NM_010305 | Gnai1 | guanine nucleotide binding protein (G protein), alpha inhibiting 1 | -1,07 | 4,15E-05 |
| 1416978_at | NM_010189 | Fcgrt | Fc receptor, IgG, alpha chain transporter | -1,07 | 1,70E-04 |
| 1434451_at | XM_003084921 | Gm10786 | predicted gene 10786 | -1,07 | 1,98E-05 |
| 1454736_at | NM_172939 | Ankrd57 | ankyrin repeat domain 57 | -1,06 | 1,74E-03 |
| 1421184_a_at | NM_001002900 | AB099516 /// Gm10035 /// Higd1c /// Mettl7a1 /// Mettl7a2 | cDNA sequence AB099516 /// predicted gene 10035 /// HIG1 domain family, member 1C /// methyltransferase like 7A1 /// methyltransferase like 7A2 | -1,06 | 9,73E-05 |
| 1451545_at | NM_172605 | Tdrd3 | tudor domain containing 3 | -1,06 | 1,15E-05 |
| 1435363_at | NM_001033253 | Plekhg1 | pleckstrin homology domain containing, family G (with RhoGef domain) member 1 | -1,06 | 2,03E-03 |
| 1419591_at | NM_031378 | Gsdmc | gasdermin C | -1,06 | 2,67E-04 |
| 1452888_at | NM_028637 | 1110034G24Rik | RIKEN cDNA 1110034G24 gene | -1,06 | 6,19E-05 |
| 1429546_at | XR_105688 | 4930423K06Rik /// LOC100502740 | RIKEN cDNA 4930423K06 gene /// hypothetical LOC100502740 | -1,06 | 1,65E-02 |
| 1436031_at | NM_198037 | Cachd1 | cache domain containing 1 | -1,06 | 1,36E-03 |
| 1423244_at | NM_001039555 | Cyp2c68 | cytochrome P450, family 2, subfamily c, polypeptide 68 | -1,06 | 1,15E-06 |
| 1415793_at | NM_134021 | Pnpo | pyridoxine 5'-phosphate oxidase | -1,06 | 3,11E-04 |
| 1427217_at | NM_001048204 | Zfp455 | zinc finger protein 455 | -1,06 | 1,23E-03 |
| 1455642_a_at | NM_028841 | Tspan17 | tetraspanin 17 | -1,06 | 5,51E-04 |
| 1455567_at | NM_001109626 | Cdk12 /// LOC100502717 | cyclin-dependent kinase 12 /// hypothetical LOC100502717 | -1,06 | 8,10E-06 |
| 1427075_s_at | NM_153594 | Pcmtd2 | protein-L-isoaspartate (D-aspartate) O-methyltransferase domain containing 2 | -1,06 | 6,04E-03 |
| 1417780_at | NM_026058 | Lass4 | LAG1 homolog, ceramide synthase 4 | -1,06 | 2,86E-05 |
| 1418213_at | NM_033373 | Krt23 | keratin 23 | -1,06 | 1,11E-05 |
| 1452716_at | NM_027464 | 5730469M10Rik | RIKEN cDNA 5730469M10 gene | -1,06 | 3,03E-04 |
| 1448107_x_at | NM_010639 | Klk1 | kallikrein 1 | -1,06 | 1,35E-06 |
| 1424077_at | NM_025638 | Gdpd1 | glycerophosphodiester phosphodiesterase domain containing 1 | -1,06 | 3,80E-05 |
| 1415997_at | NM_001009935 | Txnip | thioredoxin interacting protein | -1,05 | 2,11E-04 |
| 1435050_at | NM_001033258 | D10Bwg1379e | DNA segment, Chr 10, Brigham & Women's Genetics 1379 expressed | -1,05 | 7,45E-06 |
| 1453003_at | NM_011436 | Sorl1 | sortilin-related receptor, LDLR class A repeats-containing | -1,05 | 1,06E-04 |
| 1427074_at | NM_153594 | Pcmtd2 | protein-L-isoaspartate (D-aspartate) O-methyltransferase domain containing 2 | -1,05 | 2,80E-04 |
| 1435671_at | NM_001164370 | Mipol1 | mirror-image polydactyly gene 1 homolog (human) | -1,05 | 1,77E-03 |
| 1423508_at | NM_017479 | Myst4 | MYST histone acetyltransferase monocytic leukemia 4 | -1,05 | 4,53E-06 |
| 1439540_at | NM_145486 | March2 | membrane-associated ring finger (C3HC4) 2 | -1,05 | 8,89E-04 |
| 1425452_s_at | NM_029007 | Fam84a | family with sequence similarity 84, member A | -1,05 | 5,06E-05 |
| 1449530_at | NM_032000 | Trps1 | trichorhinophalangeal syndrome I (human) | -1,05 | 6,52E-03 |
| 1444307_at | --- | --- | --- | -1,05 | 6,05E-04 |
| 1428375_at | NR_015524 | 4932415G12Rik | RIKEN cDNA 4932415G12 gene | -1,05 | 1,69E-04 |
| 1450825_at | NM_175731 | Acer1 | alkaline ceramidase 1 | -1,05 | 6,29E-05 |
| 1420620_a_at | NM_001113413 | Rnf13 | ring finger protein 13 | -1,05 | 3,35E-05 |
| 1434373_at | NM_178764 | Fam168a | family with sequence similarity 168, member A | -1,04 | 5,54E-06 |
| 1416383_a_at | NM_001162946 | Pcx | pyruvate carboxylase | -1,04 | 9,93E-06 |
| 1425281_a_at | NM_001077364 | Tsc22d3 | TSC22 domain family, member 3 | -1,04 | 3,25E-05 |
| 1455876_at | NM_001033270 | Slc4a7 | solute carrier family 4, sodium bicarbonate cotransporter, member 7 | -1,04 | 1,23E-03 |
| 1455362_at | NM_021421 | Angel2 | angel homolog 2 (Drosophila) | -1,04 | 5,00E-04 |
| 1428386_at | NM_001033606 | Acsl3 | acyl-CoA synthetase long-chain family member 3 | -1,04 | 2,05E-04 |
| 1434441_at | NM_025370 | 1110018J18Rik | RIKEN cDNA 1110018J18 gene | -1,04 | 3,26E-04 |
| 1454049_at | NR_029414 | Gsdmcl-ps | gasdermin C-like, pseudogene | -1,04 | 6,18E-03 |
| 1429005_at | NM_001081279 | Mfhas1 | malignant fibrous histiocytoma amplified sequence 1 | -1,04 | 4,37E-05 |
| 1453247_at | NM_028326 | Zfp618 | zinc fingerprotein 618 | -1,04 | 1,25E-04 |
| 1427471_at | NM_015822 | Fbxl3 | F-box and leucine-rich repeat protein 3 | -1,04 | 4,59E-03 |
| 1453293_a_at | NM_027419 | 2810408A11Rik | RIKEN cDNA 2810408A11 gene | -1,04 | 5,31E-03 |
| 1417061_at | NM_016917 | Slc40a1 | solute carrier family 40 (iron-regulated transporter), member 1 | -1,04 | 1,30E-05 |
| 1453054_at | NM_029153 | Scamp1 | secretory carrier membrane protein 1 | -1,04 | 1,15E-03 |
| 1429351_at | NM_029436 | Klhl24 | kelch-like 24 (Drosophila) | -1,04 | 1,58E-04 |
| 1450719_at | NM_008585 | Mep1a | meprin 1 alpha | -1,04 | 8,40E-06 |
| 1416895_at | NM_001162425 | Efna1 | ephrin A1 | -1,03 | 1,97E-06 |
| 1451739_at | NM_009769 | Klf5 | Kruppel-like factor 5 | -1,03 | 1,02E-03 |
| 1416411_at | NM_008183 | Gstm2 | glutathione S-transferase, mu 2 | -1,03 | 1,26E-04 |
| 1439561_at | NM_025563 | 2010012O05Rik | RIKEN cDNA 2010012O05 gene | -1,03 | 2,54E-05 |
| 1460608_at | NM_001042528 | Cacna1b | calcium channel, voltage-dependent, N type, alpha 1B subunit | -1,03 | 1,55E-03 |
| 1416441_at | NM_018755 | Pgcp | plasma glutamate carboxypeptidase | -1,03 | 4,68E-04 |
| 1435400_at | NM_030110 | Efha2 | EF-hand domain family, member A2 | -1,03 | 4,01E-04 |
| 1443934_at | NM_199017 | 9230110C19Rik | RIKEN cDNA 9230110C19 gene | -1,03 | 2,73E-03 |
| 1430172_a_at | NM_001100187 | Cyp4f16 /// Gm9705 | cytochrome P450, family 4, subfamily f, polypeptide 16 /// predicted gene 9705 | -1,03 | 5,78E-05 |
| 1457054_a_at | XM_003085575 | LOC100504016 | hypothetical protein LOC100504016 | -1,03 | 2,55E-03 |
| 1448510_at | NM_001162425 | Efna1 | ephrin A1 | -1,03 | 4,43E-04 |
| 1422809_at | NM_053271 | Rims2 | regulating synaptic membrane exocytosis 2 | -1,03 | 1,52E-02 |
| 1436321_at | NM_145222 | B3gnt7 | UDP-GlcNAc:betaGal beta-1,3-N-acetylglucosaminyltransferase 7 | -1,03 | 1,76E-03 |
| 1453801_at | NM_025416 | Them5 | thioesterase superfamily member 5 | -1,03 | 5,34E-04 |
| 1448852_at | NM_009060 | Rgn | regucalcin | -1,03 | 3,02E-04 |
| 1451653_a_at | NM_028672 | Fam161a | family with sequence similarity 161, member A | -1,03 | 1,45E-03 |
| 1430232_at | --- | 4933407E14Rik | RIKEN cDNA 4933407E14 gene | -1,03 | 1,01E-02 |
| 1419082_at | NM_001174170 | Serpinb2 | serine (or cysteine) peptidase inhibitor, clade B, member 2 | -1,03 | 1,25E-03 |
| 1419175_a_at | NM_013483 | Btn1a1 | butyrophilin, subfamily 1, member A1 | -1,03 | 3,96E-03 |
| 1431143_x_at | NM_001039194 | Aifm2 | apoptosis-inducing factor, mitochondrion-associated 2 | -1,03 | 9,38E-04 |
| 1435085_at | NM_177687 | Crebl2 | cAMP responsive element binding protein-like 2 | -1,02 | 1,01E-03 |
| 1435370_a_at | NM_053200 | Ces1d | carboxylesterase 1D | -1,02 | 3,82E-04 |
| 1415837_at | NM_010639 | Klk1 | kallikrein 1 | -1,02 | 6,96E-07 |
| 1434499_a_at | NM_008492 | Ldhb | lactate dehydrogenase B | -1,02 | 3,14E-04 |
| 1456072_at | NM_181595 | Ppp1r9a | protein phosphatase 1, regulatory (inhibitor) subunit 9A | -1,02 | 1,84E-04 |
| 1447166_at | --- | --- | --- | -1,02 | 1,23E-02 |
| 1417030_at | NM_025864 | Tmem206 | transmembrane protein 206 | -1,02 | 1,31E-03 |
| 1456779_a_at | --- | 4930414L22Rik | RIKEN cDNA 4930414L22 gene | -1,02 | 1,56E-03 |
| 1444790_at | NM_001136181 | Hsbp1l1 | heat shock factor binding protein 1-like 1 | -1,02 | 3,00E-04 |
| 1421127_at | NM_001164823 | Tmem42 | transmembrane protein 42 | -1,02 | 2,06E-03 |
| 1452445_at | NM_177388 | Slc41a2 | solute carrier family 41, member 2 | -1,02 | 9,11E-05 |
| 1448566_at | NM_016917 | Slc40a1 | solute carrier family 40 (iron-regulated transporter), member 1 | -1,02 | 2,38E-04 |
| 1444815_at | --- | C85328 | expressed sequence C85328 | -1,02 | 4,48E-04 |
| 1452700_s_at | NM_001024135 | Kbtbd7 | kelch repeat and BTB (POZ) domain containing 7 | -1,01 | 9,15E-06 |
| 1443790_x_at | --- | 4930414L22Rik | RIKEN cDNA 4930414L22 gene | -1,01 | 1,89E-03 |
| 1459670_at | --- | --- | --- | -1,01 | 8,55E-04 |
| 1426624_a_at | NM_025347 | Ypel3 | yippee-like 3 (Drosophila) | -1,01 | 5,53E-04 |
| 1439347_at | NM_172564 | Tns4 | tensin 4 | -1,01 | 3,51E-06 |
| 1448558_a_at | NM_008869 | Pla2g4a | phospholipase A2, group IVA (cytosolic, calcium-dependent) | -1,01 | 2,71E-04 |
| 1436897_at | NM_001081279 | Mfhas1 | malignant fibrous histiocytoma amplified sequence 1 | -1,01 | 1,12E-04 |
| 1453571_at | NM_001037937 | Depdc6 | DEP domain containing 6 | -1,01 | 6,95E-05 |
| 1435213_at | NM_175340 | Nhlrc1 | NHL repeat containing 1 | -1,01 | 2,75E-04 |
| 1419040_at | NM_001163472 | Cyp2d22 | cytochrome P450, family 2, subfamily d, polypeptide 22 | -1,01 | 6,70E-04 |
| 1457284_at | --- | --- | --- | -1,01 | 1,23E-04 |
| 1427946_s_at | NM_170778 | Dpyd | dihydropyrimidine dehydrogenase | -1,01 | 2,52E-05 |
| 1420859_at | NM_008862 | Pkia | protein kinase inhibitor, alpha | -1,01 | 4,65E-03 |
| 1441945_s_at | NM_001110271 | Abhd14a | abhydrolase domain containing 14A | -1,01 | 2,39E-06 |
| 1443579_s_at | NM_001037937 | Depdc6 /// LOC100505173 | DEP domain containing 6 /// hypothetical LOC100505173 | -1,01 | 9,68E-04 |
| 1438856_x_at | NM_009257 | Serpinb5 | serine (or cysteine) peptidase inhibitor, clade B, member 5 | -1,01 | 1,85E-03 |
| 1418996_a_at | NM_001163628 | Lyrm5 | LYR motif containing 5 | -1,01 | 1,13E-06 |
| 1438187_at | NM_181328 | Slc25a29 | solute carrier family 25 (mitochondrial carrier, palmitoylcarnitine transporter), member 29 | -1,01 | 2,57E-04 |
| 1436060_at | NM_001163328 | Gm15706 /// Ralyl | predicted gene 15706 /// RALY RNA binding protein-like | -1,01 | 3,54E-03 |
| 1424794_at | NM_025786 | Rnf186 | ring finger protein 186 | -1,00 | 4,76E-06 |
| 1441316_at | NM_011720 | Wnt8b | wingless related MMTV integration site 8b | -1,00 | 1,65E-03 |
| 1447308_at | NM_028015 | Lass5 | LAG1 homolog, ceramide synthase 5 | -1,00 | 3,91E-03 |
| 1417529_at | NM_011228 | Rab33a | RAB33A, member of RAS oncogene family | -1,00 | 3,12E-03 |
| 1433971_at | NM_001081557 | Camta1 | calmodulin binding transcription activator 1 | -1,00 | 3,43E-03 |
| 1420503_at | NM_020049 | Slc6a14 | solute carrier family 6 (neurotransmitter transporter), member 14 | -1,00 | 8,06E-04 |
| 1430641_at | NM_001168274 | Gsdmc2 /// Gsdmc4 | gasdermin C2 /// gasdermin C4 | -1,00 | 2,15E-04 |
| 1435939_s_at | NM_178899 | Hepacam2 | HEPACAM family member 2 | -1,00 | 1,91E-04 |
| 1455627_at | NM_007739 | Col8a1 | collagen, type VIII, alpha 1 | -1,00 | 1,31E-05 |
| 1428781_at | NM_001166173 | Dmkn | dermokine | -1,00 | 4,53E-04 |
| 1454045_a_at | NM_133757 | Pgs1 | phosphatidylglycerophosphate synthase 1 | 1,00 | 3,37E-05 |
| 1450862_at | NM_001122958 | Rad54l | RAD54 like (S. cerevisiae) | 1,00 | 3,33E-03 |
| 1427222_a_at | NM_009300 | Svs4 | seminal vesicle secretory protein 4 | 1,00 | 6,27E-04 |
| 1421009_at | NM_021384 | Rsad2 | radical S-adenosyl methionine domain containing 2 | 1,00 | 1,91E-04 |
| 1418531_at | NM_133353 | Oosp1 | oocyte secreted protein 1 | 1,00 | 1,80E-03 |
| 1438866_at | NM_001033351 | Grin3a | glutamate receptor ionotropic, NMDA3A | 1,00 | 5,22E-03 |
| 1426754_x_at | NM_175451 | Ckap4 | cytoskeleton-associated protein 4 | 1,00 | 7,09E-04 |
| 1425802_a_at | NM_001160215 | Fcrla | Fc receptor-like A | 1,00 | 5,13E-04 |
| 1419604_at | NM_001139519 | Zbp1 | Z-DNA binding protein 1 | 1,00 | 4,02E-05 |
| 1416234_at | NM_133807 | Lrrc59 | leucine rich repeat containing 59 | 1,00 | 1,05E-04 |
| 1460233_at | NM_008191 | Guca2b | guanylate cyclase activator 2b (retina) | 1,01 | 3,18E-04 |
| 1441779_at | --- | 9530006C21Rik | RIKEN cDNA 9530006C21 gene | 1,01 | 1,69E-04 |
| 1421260_a_at | NM_009272 | Srm | spermidine synthase | 1,01 | 1,14E-03 |
| 1426324_at | NM_010380 | H2-D1 | histocompatibility 2, D region locus 1 | 1,01 | 1,18E-04 |
| 1425156_at | NM_001083312 | Gbp6 | guanylate binding protein 6 | 1,01 | 2,93E-03 |
| 1444026_at | NM_178906 | AI593442 | expressed sequence AI593442 | 1,01 | 6,32E-03 |
| 1452183_a_at | NR_003633 | Meg3 | maternally expressed 3 | 1,01 | 2,13E-03 |
| 1458299_s_at | NM_008690 | Nfkbie | nuclear factor of kappa light polypeptide gene enhancer in B-cells inhibitor, epsilon | 1,01 | 2,16E-04 |
| 1433779_at | NM_177054 | Casc4 | cancer susceptibility candidate 4 | 1,01 | 3,36E-04 |
| 1426454_at | NM_007486 | Arhgdib | Rho, GDP dissociation inhibitor (GDI) beta | 1,01 | 3,59E-03 |
| 1422604_at | NM_009474 | Uox | urate oxidase | 1,01 | 1,82E-04 |
| 1417619_at | NM_183358 | Gadd45gip1 | growth arrest and DNA-damage-inducible, gamma interacting protein 1 | 1,01 | 1,14E-04 |
| 1437025_at | NM_007642 | Cd28 | CD28 antigen | 1,01 | 1,62E-03 |
| 1427540_at | NM_025635 | Zwint | ZW10 interactor | 1,01 | 4,22E-04 |
| 1426772_x_at | XM_977361 | Gm6273 /// LOC381765 /// LOC665506 /// Tcrb-J | predicted gene 6273 /// similar to T cell antigen receptor /// similar to T-cell receptor beta-2 chain C region /// T-cell receptor beta, joining region | 1,01 | 4,73E-04 |
| 1449071_at | NM_022879 | Myl7 | myosin, light polypeptide 7, regulatory | 1,01 | 3,90E-05 |
| 1436387_at | NM_011982 | C330006P03Rik /// Homer1 | RIKEN cDNA C330006P03 gene /// homer homolog 1 (Drosophila) | 1,02 | 6,94E-03 |
| 1422612_at | NM_013820 | Hk2 | hexokinase 2 | 1,02 | 4,32E-05 |
| 1420394_s_at | NM_008147 | Gp49a /// Lilrb4 | glycoprotein 49 A /// leukocyte immunoglobulin-like receptor, subfamily B, member 4 | 1,02 | 8,81E-03 |
| 1448191_at | NM_011121 | Plk1 | polo-like kinase 1 (Drosophila) | 1,02 | 5,16E-05 |
| 1431693_a_at | NM_019508 | Il17b | interleukin 17B | 1,02 | 2,24E-04 |
| 1435176_a_at | NM_010496 | Id2 | inhibitor of DNA binding 2 | 1,02 | 7,92E-04 |
| 1441915_s_at | NM_001077348 | Plin5 | perilipin 5 | 1,02 | 7,97E-04 |
| 1443536_at | NM_011990 | Slc7a11 | solute carrier family 7 (cationic amino acid transporter, y+ system), member 11 | 1,02 | 8,26E-03 |
| 1424374_at | NM_174990 | Gimap4 | GTPase, IMAP family member 4 | 1,02 | 1,17E-03 |
| 1438095_x_at | NM_153570 | Noc4l | Nucleolar complex associated 4 homolog (S. cerevisiae) | 1,02 | 1,78E-04 |
| 1440068_at | --- | --- | --- | 1,02 | 2,86E-03 |
| 1442849_at | NM_008512 | Lrp1 | low density lipoprotein receptor-related protein 1 | 1,02 | 1,14E-03 |
| 1429831_at | NM_031376 | Pik3ap1 | phosphoinositide-3-kinase adaptor protein 1 | 1,02 | 1,40E-04 |
| 1415857_at | NM_010330 | Emb | embigin | 1,02 | 7,63E-05 |
| 1448660_at | NM_008113 | Arhgdig | Rho GDP dissociation inhibitor (GDI) gamma | 1,03 | 3,82E-04 |
| 1417185_at | NM_010738 | Ly6a | lymphocyte antigen 6 complex, locus A | 1,03 | 1,26E-04 |
| 1418326_at | NM_011404 | Slc7a5 | solute carrier family 7 (cationic amino acid transporter, y+ system), member 5 | 1,03 | 5,10E-04 |
| 1451962_at | --- | Igk-V19-20 | immunoglobulin kappa chain variable 19 (V19)-20 | 1,03 | 3,79E-04 |
| 1431591_s_at | NM_015783 | Gm9706 /// Isg15 | predicted gene 9706 /// ISG15 ubiquitin-like modifier | 1,03 | 6,06E-05 |
| 1454046_x_at | NM_133757 | Pgs1 | phosphatidylglycerophosphate synthase 1 | 1,03 | 9,61E-05 |
| 1451046_at | NM_009569 | Zfpm1 | zinc finger protein, multitype 1 | 1,03 | 2,16E-05 |
| 1454268_a_at | NM_007806 | Cyba | cytochrome b-245, alpha polypeptide | 1,03 | 2,19E-05 |
| 1423714_at | NM_024184 | Asf1b | ASF1 anti-silencing function 1 homolog B (S. cerevisiae) | 1,03 | 7,70E-06 |
| 1426658_x_at | NM_016966 | Phgdh | 3-phosphoglycerate dehydrogenase | 1,03 | 3,54E-04 |
| 1424041_s_at | NM_001097617 | C1s | complement component 1, s subcomponent | 1,03 | 2,60E-05 |
| 1430388_a_at | NM_028072 | Sulf2 | sulfatase 2 | 1,03 | 3,19E-03 |
| 1428750_at | NM_026772 | Cdc42ep2 | CDC42 effector protein (Rho GTPase binding) 2 | 1,03 | 7,14E-04 |
| 1416512_at | NM_011956 | Nubp2 | nucleotide binding protein 2 | 1,04 | 9,99E-05 |
| 1420915_at | NM_009283 | Stat1 | signal transducer and activator of transcription 1 | 1,04 | 7,61E-06 |
| 1440275_at | NM_019732 | Runx3 | runt related transcription factor 3 | 1,04 | 6,61E-05 |
| 1429381_x_at | --- | Igh-2 /// Igh-VJ558 /// LOC544903 | immunoglobulin heavy chain 2 (serum IgA) /// immunoglobulin heavy chain (J558 family) /// similar to immunoglobulin mu-chain | 1,04 | 8,97E-06 |
| 1456498_at | NM_010576 | Itga4 | integrin alpha 4 | 1,04 | 3,35E-03 |
| 1433512_at | NM_008026 | Fli1 | Friend leukemia integration 1 | 1,04 | 9,18E-04 |
| 1455229_x_at | NM_133757 | Pgs1 | phosphatidylglycerophosphate synthase 1 | 1,04 | 6,50E-04 |
| 1454737_at | NM_029352 | Dusp9 | dual specificity phosphatase 9 | 1,04 | 8,01E-04 |
| 1423756_s_at | NM_010517 | Igfbp4 | insulin-like growth factor binding protein 4 | 1,05 | 1,57E-05 |
| 1418987_at | NM_011109 | Pla2g2d | phospholipase A2, group IID | 1,05 | 6,89E-05 |
| 1429787_x_at | NM_025635 | Zwint | ZW10 interactor | 1,05 | 2,83E-05 |
| 1452574_x_at | --- | Igh-VJ558 | Immunoglobulin heavy chain (J558 family) | 1,05 | 5,43E-04 |
| 1416184_s_at | NM_001025427 | Hmga1 /// Hmga1-rs1 | high mobility group AT-hook 1 /// high mobility group AT-hook I, related sequence 1 | 1,05 | 1,73E-05 |
| 1420819_at | NM_001029841 | Sla | src-like adaptor | 1,05 | 6,31E-04 |
| 1418641_at | NM_010696 | Lcp2 | lymphocyte cytosolic protein 2 | 1,05 | 1,47E-03 |
| 1416296_at | NM_013563 | Il2rg | interleukin 2 receptor, gamma chain | 1,06 | 3,98E-04 |
| 1444088_at | --- | --- | --- | 1,06 | 8,27E-04 |
| 1437103_at | NM_183029 | Igf2bp2 | insulin-like growth factor 2 mRNA binding protein 2 | 1,06 | 5,72E-05 |
| 1453238_s_at | NR_002860 | 3930401B19Rik /// A130040M12Rik /// E430024C06Rik | RIKEN cDNA 3930401B19 gene /// RIKEN cDNA A130040M12 gene /// RIKEN cDNA E430024C06 gene | 1,06 | 2,22E-04 |
| 1415909_at | NM_016737 | Stip1 | stress-induced phosphoprotein 1 | 1,06 | 3,83E-05 |
| 1434239_at | NM_199447 | Rrp12 | ribosomal RNA processing 12 homolog (S. cerevisiae) | 1,06 | 1,74E-05 |
| 1419152_at | NM_026515 | 2810417H13Rik | RIKEN cDNA 2810417H13 gene | 1,06 | 1,87E-04 |
| 1415977_at | NM_023627 | Isyna1 | myo-inositol 1-phosphate synthase A1 | 1,06 | 2,02E-04 |
| 1417821_at | NM_033075 | D17H6S56E-5 | DNA segment, Chr 17, human D6S56E 5 | 1,06 | 4,28E-06 |
| 1435630_s_at | NM_009338 | Acat2 | acetyl-Coenzyme A acetyltransferase 2 | 1,06 | 3,48E-05 |
| 1441811_x_at | NM_001098271 | Tmem176a | transmembrane protein 176A | 1,06 | 1,70E-03 |
| 1431521_at | XM_129208 | Ms4a18 | membrane-spanning 4-domains, subfamily A, member 18 | 1,07 | 1,34E-02 |
| 1450639_at | NM_001085518 | Gm14085 /// Slc28a2 | predicted gene 14085 /// solute carrier family 28 (sodium-coupled nucleoside transporter), member 2 | 1,07 | 7,96E-03 |
| 1433601_at | NM_007417 | Adra2a | adrenergic receptor, alpha 2a | 1,07 | 9,37E-03 |
| 1459872_x_at | NM_010386 | H2-DMa | histocompatibility 2, class II, locus DMa | 1,07 | 4,35E-03 |
| 1418465_at | NM_008677 | Ncf4 | neutrophil cytosolic factor 4 | 1,07 | 1,51E-03 |
| 1417391_a_at | NM_010551 | Il16 | interleukin 16 | 1,07 | 1,39E-04 |
| 1422978_at | NM_007807 | Cybb | cytochrome b-245, beta polypeptide | 1,07 | 1,44E-02 |
| 1426755_at | NM_175451 | Ckap4 | cytoskeleton-associated protein 4 | 1,07 | 2,66E-04 |
| 1454904_at | NM_001164190 | Mtm1 | X-linked myotubular myopathy gene 1 | 1,07 | 1,27E-05 |
| 1421571_a_at | NM_001099217 | Ly6c1 /// Ly6c2 | lymphocyte antigen 6 complex, locus C1 /// lymphocyte antigen 6 complex, locus C2 | 1,07 | 7,85E-05 |
| 1449401_at | NM_007574 | C1qc | complement component 1, q subcomponent, C chain | 1,07 | 2,60E-05 |
| 1452231_x_at | NM_001170853 | Mndal | myeloid nuclear differentiation antigen like | 1,07 | 3,24E-03 |
| 1430534_at | NM_030098 | Rnase6 | ribonuclease, RNase A family, 6 | 1,08 | 3,15E-03 |
| 1422849_a_at | NM_019402 | Pabpn1 | poly(A) binding protein, nuclear 1 | 1,08 | 4,30E-02 |
| 1435748_at | NM_010266 | Gda | guanine deaminase | 1,08 | 3,85E-05 |
| 1434380_at | NM_001083312 | Gbp6 | guanylate binding protein 6 | 1,08 | 3,98E-04 |
| 1426225_at | NM_001159487 | Rbp4 | retinol binding protein 4, plasma | 1,08 | 7,90E-05 |
| 1417745_at | NM_030703 | Cpn1 | carboxypeptidase N, polypeptide 1 | 1,08 | 3,28E-03 |
| 1439680_at | NM_009425 | Tnfsf10 | tumor necrosis factor (ligand) superfamily, member 10 | 1,08 | 4,16E-03 |
| 1434366_x_at | NM_009777 | C1qb | complement component 1, q subcomponent, beta polypeptide | 1,09 | 6,44E-03 |
| 1453748_a_at | NM_024245 | Kif23 | kinesin family member 23 | 1,09 | 1,83E-03 |
| 1424518_at | NM_001162883 | Apol9a /// Apol9b | apolipoprotein L 9a /// apolipoprotein L 9b | 1,09 | 6,92E-04 |
| 1455814_x_at | NM_197982 | Ddx39 | DEAD (Asp-Glu-Ala-Asp) box polypeptide 39 | 1,09 | 2,06E-04 |
| 1439483_at | NR_015554 | AI506816 | expressed sequence AI506816 | 1,09 | 1,17E-03 |
| 1429889_at | NM_026976 | Faim3 | Fas apoptotic inhibitory molecule 3 | 1,09 | 1,99E-04 |
| 1422637_at | NM_018750 | Rassf5 | Ras association (RalGDS/AF-6) domain family member 5 | 1,09 | 6,17E-03 |
| 1424629_at | NM_009764 | Brca1 | breast cancer 1 | 1,09 | 5,56E-05 |
| 1433408_a_at | NM_027290 | Mcm10 | minichromosome maintenance deficient 10 (S. cerevisiae) | 1,09 | 3,12E-03 |
| 1450860_at | NM_024434 | Lap3 | leucine aminopeptidase 3 | 1,09 | 1,61E-04 |
| 1448620_at | NM_010188 | Fcgr3 | Fc receptor, IgG, low affinity III | 1,10 | 2,76E-03 |
| 1433600_at | NM_007417 | Adra2a | adrenergic receptor, alpha 2a | 1,10 | 6,12E-04 |
| 1438055_at | NM_001164763 | Rarres1 | retinoic acid receptor responder (tazarotene induced) 1 | 1,10 | 1,79E-02 |
| 1419135_at | NM_008518 | Ltb | lymphotoxin B | 1,10 | 2,04E-04 |
| 1439816_at | XM_003084430 | Gm10567 | predicted gene 10567 | 1,10 | 1,73E-03 |
| 1417063_at | NM_009777 | C1qb | complement component 1, q subcomponent, beta polypeptide | 1,10 | 9,10E-04 |
| 1448562_at | NM_001159401 | Upp1 | uridine phosphorylase 1 | 1,10 | 8,65E-06 |
| 1429233_at | NM_001009818 | sept11 | septin 11 | 1,10 | 4,31E-05 |
| 1460185_at | NM_016879 | Krt85 | keratin 85 | 1,10 | 8,54E-05 |
| 1460677_at | NM_139140 | Spats2 | spermatogenesis associated, serine-rich 2 | 1,10 | 2,32E-04 |
| 1426025_s_at | NM_010686 | Laptm5 | lysosomal-associated protein transmembrane 5 | 1,11 | 1,16E-05 |
| 1451683_x_at | NM_010380 | H2-D1 | histocompatibility 2, D region locus 1 | 1,11 | 8,29E-04 |
| 1417494_a_at | NM_001042611 | Cp | ceruloplasmin | 1,11 | 8,76E-04 |
| 1418945_at | NM_010809 | Mmp3 | matrix metallopeptidase 3 | 1,11 | 8,00E-05 |
| 1454157_a_at | NM_011109 | Pla2g2d | phospholipase A2, group IID | 1,11 | 4,34E-04 |
| 1435951_at | NM_028736 | Grip1 | glutamate receptor interacting protein 1 | 1,11 | 2,65E-03 |
| 1421217_a_at | NM_001159301 | Lgals9 | lectin, galactose binding, soluble 9 | 1,11 | 1,85E-04 |
| 1451970_at | NM_144915 | Daglb | diacylglycerol lipase, beta | 1,11 | 2,05E-04 |
| 1420425_at | NM_007548 | Prdm1 | PR domain containing 1, with ZNF domain | 1,12 | 9,31E-04 |
| 1424609_a_at | XM_001479755 | Gm4354 | predicted gene 4354 | 1,12 | 1,87E-05 |
| 1434955_at | NM_001166372 | March1 | membrane-associated ring finger (C3HC4) 1 | 1,12 | 1,91E-03 |
| 1424138_at | NM_010117 | Rhbdf1 | rhomboid family 1 (Drosophila) | 1,12 | 1,24E-03 |
| 1419872_at | NM_001037859 | Csf1r | colony stimulating factor 1 receptor | 1,12 | 6,09E-06 |
| 1439956_at | NM_027209 | Ms4a6b | membrane-spanning 4-domains, subfamily A, member 6B | 1,12 | 2,97E-03 |
| 1459842_x_at | NM_011956 | Nubp2 | nucleotide binding protein 2 | 1,12 | 1,01E-03 |
| 1452834_at | NM_001083810 | Prr5l | proline rich 5 like | 1,12 | 1,18E-02 |
| 1418681_at | NM_026247 | Alg13 | asparagine-linked glycosylation 13 homolog (S. cerevisiae) | 1,12 | 8,13E-03 |
| 1448754_at | NM_011254 | Rbp1 | retinol binding protein 1, cellular | 1,12 | 6,13E-04 |
| 1449153_at | NM_008605 | Mmp12 | matrix metallopeptidase 12 | 1,12 | 3,39E-03 |
| 1448160_at | NM_008879 | Lcp1 | lymphocyte cytosolic protein 1 | 1,12 | 2,18E-05 |
| 1450034_at | NM_009283 | Stat1 | signal transducer and activator of transcription 1 | 1,12 | 3,08E-05 |
| 1417884_at | NM_001029842 | Slc16a6 | solute carrier family 16 (monocarboxylic acid transporters), member 6 | 1,12 | 1,80E-03 |
| 1443929_at | --- | 9830004L10Rik | RIKEN cDNA 9830004L10 gene | 1,12 | 1,99E-03 |
| 1418126_at | NM_013653 | Ccl5 | chemokine (C-C motif) ligand 5 | 1,13 | 2,08E-03 |
| 1419202_at | NM_009977 | Cst7 | cystatin F (leukocystatin) | 1,13 | 1,33E-03 |
| 1432466_a_at | NM_009696 | Apoe | apolipoprotein E | 1,13 | 4,53E-04 |
| 1418282_x_at | NM_009244 | Serpina1b | serine (or cysteine) preptidase inhibitor, clade A, member 1B | 1,13 | 3,41E-03 |
| 1442923_at | NM_009184 | Ptk6 | PTK6 protein tyrosine kinase 6 | 1,13 | 5,87E-03 |
| 1427455_x_at | --- | Gm10883 /// Gm1420 /// Gm7202 /// Igk-C /// Igk-J1 /// Igk-V28 | predicted gene 10883 /// predicted gene 1420 /// predicted gene 7202 /// immunoglobulin kappa chain, constant region /// immunoglobulin kappa chain, joining region, 1 /// immunoglobulin kappa chain variable 28 (V28) | 1,13 | 5,11E-05 |
| 1424607_a_at | XM_001479755 | Gm4354 | predicted gene 4354 | 1,13 | 4,58E-04 |
| 1417867_at | NM_013459 | Cfd | complement factor D (adipsin) | 1,14 | 8,87E-06 |
| 1416235_at | NM_133807 | Lrrc59 | leucine rich repeat containing 59 | 1,14 | 1,30E-04 |
| 1424291_at | NM_172410 | Nup93 | nucleoporin 93 | 1,14 | 1,16E-03 |
| 1445001_at | --- | --- | --- | 1,14 | 5,60E-03 |
| 1424754_at | NM_001025610 | Ms4a7 | membrane-spanning 4-domains, subfamily A, member 7 | 1,14 | 2,62E-05 |
| 1455660_at | NM_007780 | Csf2rb | colony stimulating factor 2 receptor, beta, low-affinity (granulocyte-macrophage) | 1,14 | 5,16E-04 |
| 1428304_at | NM_028039 | Esco2 | establishment of cohesion 1 homolog 2 (S. cerevisiae) | 1,14 | 6,50E-04 |
| 1424375_s_at | NM_174990 | Gimap4 | GTPase, IMAP family member 4 | 1,14 | 9,80E-04 |
| 1418826_at | NM_027209 | Ms4a6b | membrane-spanning 4-domains, subfamily A, member 6B | 1,14 | 4,14E-03 |
| 1428821_at | NM_026212 | Agpat2 | 1-acylglycerol-3-phosphate O-acyltransferase 2 (lysophosphatidic acid acyltransferase, beta) | 1,14 | 4,91E-04 |
| 1433733_a_at | NM_007771 | Cry1 | cryptochrome 1 (photolyase-like) | 1,14 | 3,23E-05 |
| 1435330_at | NM_175026 | Pyhin1 | pyrin and HIN domain family, member 1 | 1,14 | 3,09E-03 |
| 1422515_at | NM_020264 | Pate4 | prostate and testis expressed 4 | 1,15 | 2,72E-03 |
| 1422682_s_at | NM_001038997 | Gm10334 /// Gm5771 /// Prss1 /// Prss3 | predicted gene 10334 /// predicted gene 5771 /// protease, serine, 1 (trypsin 1) /// protease, serine, 3 | 1,15 | 4,61E-03 |
| 1459913_at | NM_009425 | Tnfsf10 | tumor necrosis factor (ligand) superfamily, member 10 | 1,15 | 5,95E-05 |
| 1451065_a_at | NM_197982 | Ddx39 | DEAD (Asp-Glu-Ala-Asp) box polypeptide 39 | 1,15 | 1,42E-04 |
| 1427348_at | NM_153159 | Zc3h12a | zinc finger CCCH type containing 12A | 1,15 | 8,66E-05 |
| 1452848_at | NM_001033178 | Tmem181a /// Tmem181b-ps | transmembrane protein 181A /// transmembrane protein 181B, pseudogene | 1,15 | 1,45E-04 |
| 1450136_at | NM_007646 | Cd38 | CD38 antigen | 1,15 | 4,29E-04 |
| 1421211_a_at | NM_007575 | Ciita | class II transactivator | 1,15 | 1,70E-03 |
| 1419347_x_at | NM_009301 | Svs5 | seminal vesicle secretory protein 5 | 1,15 | 8,25E-03 |
| 1439141_at | NM_182806 | Gpr18 | G protein-coupled receptor 18 | 1,16 | 3,51E-05 |
| 1450170_x_at | NM_010380 | H2-D1 | histocompatibility 2, D region locus 1 | 1,16 | 1,35E-05 |
| 1437886_at | NM_183390 | Klhl6 | kelch-like 6 (Drosophila) | 1,16 | 7,31E-05 |
| 1438921_at | NM_019864 | Atr | Ataxia telangiectasia and Rad3 related | 1,16 | 1,07E-02 |
| 1423643_at | NM_197982 | Ddx39 | DEAD (Asp-Glu-Ala-Asp) box polypeptide 39 | 1,16 | 1,14E-05 |
| 1452242_at | NM_001164362 | Cep55 | centrosomal protein 55 | 1,16 | 2,87E-03 |
| 1450234_at | NM_001166376 | Ms4a6c | membrane-spanning 4-domains, subfamily A, member 6C | 1,16 | 2,30E-03 |
| 1419609_at | NM_009912 | Ccr1 | chemokine (C-C motif) receptor 1 | 1,16 | 1,91E-04 |
| 1433639_at | NM_172543 | Fam117a | family with sequence similarity 117, memberA | 1,17 | 5,34E-05 |
| 1456211_at | NM_175532 | Nlrp10 | NLR family, pyrin domain containing 10 | 1,17 | 1,45E-03 |
| 1419603_at | NM_008329 | Ifi204 | interferon activated gene 204 | 1,17 | 9,56E-03 |
| 1448377_at | NM_011414 | Slpi | secretory leukocyte peptidase inhibitor | 1,17 | 6,10E-05 |
| 1426201_at | --- | Gm1499 /// Gm1524 /// Igkv4-71 | predicted gene 1499 /// predicted gene 1524 /// immunoglobulin kappa chain variable 4-71 | 1,17 | 2,05E-03 |
| 1415810_at | NM_001111078 | Uhrf1 | ubiquitin-like, containing PHD and RING finger domains, 1 | 1,17 | 6,99E-05 |
| 1417495_x_at | NM_001042611 | Cp | ceruloplasmin | 1,17 | 2,96E-03 |
| 1427857_x_at | --- | --- | --- | 1,18 | 9,84E-03 |
| 1421731_a_at | NM_007999 | Fen1 | flap structure specific endonuclease 1 | 1,18 | 5,03E-05 |
| 1449008_at | NM_011657 | Tulp3 | tubby-like protein 3 | 1,18 | 9,04E-06 |
| 1444668_at | --- | Astx | amplified spermatogenic transcripts X encoded | 1,18 | 4,33E-04 |
| 1423182_at | NM_021349 | Tnfrsf13b | tumor necrosis factor receptor superfamily, member 13b | 1,18 | 1,75E-04 |
| 1427301_at | NM_007649 | Cd48 | CD48 antigen | 1,18 | 1,02E-03 |
| 1427409_at | NM_001033262 | March9 | membrane-associated ring finger (C3HC4) 9 | 1,18 | 1,28E-03 |
| 1449486_at | NM_021456 | Ces1g | carboxylesterase 1G | 1,18 | 3,39E-04 |
| 1439764_s_at | NM_183029 | Igf2bp2 | insulin-like growth factor 2 mRNA binding protein 2 | 1,19 | 7,27E-05 |
| 1452536_s_at | --- | Gm5571 /// Igk-V1 | predicted gene 5571 /// immunoglobulin kappa chain variable 1 (V1) | 1,19 | 1,62E-03 |
| 1423134_at | NM_030259 | Rilpl2 | Rab interacting lysosomal protein-like 2 | 1,19 | 3,19E-05 |
| 1416001_a_at | NM_028071 | Cotl1 | coactosin-like 1 (Dictyostelium) | 1,19 | 8,78E-05 |
| 1437726_x_at | NM_009777 | C1qb | complement component 1, q subcomponent, beta polypeptide | 1,19 | 1,66E-04 |
| 1429909_at | NM_001083810 | Prr5l | proline rich 5 like | 1,19 | 4,88E-03 |
| 1454809_at | NM_001111267 | Ncoa7 | nuclear receptor coactivator 7 | 1,19 | 3,68E-05 |
| 1422903_at | NM_010745 | Ly86 | lymphocyte antigen 86 | 1,19 | 6,87E-05 |
| 1439965_at | NM_173388 | Slc43a2 | solute carrier family 43, member 2 | 1,19 | 5,00E-05 |
| 1422448_at | NM_009363 | Tff2 | trefoil factor 2 (spasmolytic protein 1) | 1,19 | 2,90E-03 |
| 1448728_a_at | NM_001159394 | Nfkbiz | nuclear factor of kappa light polypeptide gene enhancer in B-cells inhibitor, zeta | 1,19 | 1,35E-04 |
| 1437497_a_at | NM_010480 | Hsp90aa1 | heat shock protein 90, alpha (cytosolic), class A member 1 | 1,20 | 2,47E-04 |
| 1426113_x_at | --- | A130082M07Rik | RIKEN cDNA A130082M07 gene | 1,20 | 1,22E-02 |
| 1448441_at | NM_016904 | Cks1b | CDC28 protein kinase 1b | 1,20 | 9,94E-06 |
| 1418350_at | NM_010415 | Hbegf | heparin-binding EGF-like growth factor | 1,20 | 1,07E-04 |
| 1427398_at | NM_080457 | Muc4 | mucin 4 | 1,20 | 2,03E-04 |
| 1422436_at | NM_023333 | 2210010C04Rik | RIKEN cDNA 2210010C04 gene | 1,20 | 4,47E-03 |
| 1435331_at | NM_175026 | Pyhin1 | pyrin and HIN domain family, member 1 | 1,20 | 7,85E-03 |
| 1416514_a_at | NM_007984 | Fscn1 | fascin homolog 1, actin bundling protein (Strongylocentrotus purpuratus) | 1,20 | 1,16E-04 |
| 1416698_a_at | NM_016904 | Cks1b | CDC28 protein kinase 1b | 1,21 | 1,33E-06 |
| 1447602_x_at | NM_028072 | Sulf2 | sulfatase 2 | 1,21 | 8,92E-04 |
| 1436312_at | NM_001025597 | Ikzf1 | IKAROS family zinc finger 1 | 1,21 | 2,71E-03 |
| 1421522_at | NM_008081 | B4galnt2 | beta-1,4-N-acetyl-galactosaminyl transferase 2 | 1,21 | 7,78E-05 |
| 1435749_at | NM_010266 | Gda | guanine deaminase | 1,21 | 2,64E-04 |
| 1418773_at | NM_021890 | Fads3 | fatty acid desaturase 3 | 1,21 | 3,52E-05 |
| 1427329_a_at | --- | Igh-6 | immunoglobulin heavy chain 6 (heavy chain of IgM) | 1,22 | 2,04E-05 |
| 1419254_at | NM_008638 | Mthfd2 | methylenetetrahydrofolate dehydrogenase (NAD+ dependent), methenyltetrahydrofolate cyclohydrolase | 1,22 | 6,57E-04 |
| 1420697_at | NM_023044 | Slc15a3 | solute carrier family 15, member 3 | 1,22 | 3,75E-03 |
| 1419631_at | NM_009515 | Was | Wiskott-Aldrich syndrome homolog (human) | 1,22 | 1,99E-03 |
| 1452754_at | NM_029720 | Creld2 | cysteine-rich with EGF-like domains 2 | 1,22 | 2,02E-04 |
| 1442700_at | NM_001177980 | Pde4b | phosphodiesterase 4B, cAMP specific | 1,22 | 3,92E-04 |
| 1427076_at | NM_010821 | Mpeg1 | macrophage expressed gene 1 | 1,22 | 2,67E-05 |
| 1438040_a_at | NM_011631 | Hsp90b1 | heat shock protein 90, beta (Grp94), member 1 | 1,22 | 2,10E-04 |
| 1425079_at | NM_181540 | Tm6sf2 | transmembrane 6 superfamily member 2 | 1,22 | 5,66E-05 |
| 1425832_a_at | NM_030712 | Cxcr6 | chemokine (C-X-C motif) receptor 6 | 1,23 | 9,38E-04 |
| 1426980_s_at | NM_175332 | E130012A19Rik | RIKEN cDNA E130012A19 gene | 1,23 | 1,41E-03 |
| 1451513_x_at | NM_009243 | Serpina1a /// Serpina1b | serine (or cysteine) peptidase inhibitor, clade A, member 1A /// serine (or cysteine) preptidase inhibitor, clade A, member 1B | 1,23 | 1,57E-03 |
| 1423726_at | NM_012037 | Vat1 | vesicle amine transport protein 1 homolog (T californica) | 1,23 | 8,79E-04 |
| 1448710_at | NM_009911 | Cxcr4 | chemokine (C-X-C motif) receptor 4 | 1,23 | 7,11E-03 |
| 1431008_at | NM_207648 | H2-Q6 /// LOC68395 | histocompatibility 2, Q region locus 6 /// histocompatibility 2, Q region locus 6-like | 1,23 | 2,79E-04 |
| 1424305_at | NM_152839 | Igj | immunoglobulin joining chain | 1,24 | 9,72E-05 |
| 1443794_x_at | NM_153570 | Noc4l | nucleolar complex associated 4 homolog (S. cerevisiae) | 1,24 | 1,36E-04 |
| 1436236_x_at | NM_028071 | Cotl1 | coactosin-like 1 (Dictyostelium) | 1,24 | 5,37E-06 |
| 1436964_at | NR_015456 | D7Ertd715e | DNA segment, Chr 7, ERATO Doi 715, expressed | 1,24 | 1,14E-02 |
| 1419699_at | NM_054037 | Scgb3a1 | secretoglobin, family 3A, member 1 | 1,24 | 3,71E-04 |
| 1418842_at | NM_008225 | Hcls1 | hematopoietic cell specific Lyn substrate 1 | 1,24 | 9,49E-04 |
| 1436329_at | NM_018781 | Egr3 | early growth response 3 | 1,24 | 2,22E-04 |
| 1434152_at | NM_175391 | Apol7c /// Gm8221 | apolipoprotein L 7c /// apolipoprotein L, 3-like | 1,24 | 9,12E-04 |
| 1419627_s_at | NM_001190320 | Clec4n | C-type lectin domain family 4, member n | 1,24 | 1,14E-03 |
| 1429478_at | NM_175265 | 6720463M24Rik | RIKEN cDNA 6720463M24 gene | 1,24 | 4,83E-04 |
| 1434969_at | NM_176954 | Celf5 | CUGBP, Elav-like family member 5 | 1,24 | 1,53E-04 |
| 1428485_at | NM_178396 | Car12 | carbonic anyhydrase 12 | 1,24 | 8,06E-05 |
| 1426199_x_at | XM_003086781 | IghmAC38.205.12 /// LOC100505009 /// LOC634206 | Ig mu chain V region AC38 205.12 /// similar to immunoglobulin mu-chain-like /// ig heavy chain V region 108A-like | 1,24 | 3,64E-03 |
| 1419060_at | NM_013542 | Gzmb | granzyme B | 1,24 | 2,10E-02 |
| 1457781_at | NR_001461 | Kcnq1ot1 | KCNQ1 overlapping transcript 1 | 1,24 | 1,42E-04 |
| 1435275_at | NM_183405 | Cox6b2 | cytochrome c oxidase subunit VIb polypeptide 2 | 1,24 | 3,32E-05 |
| 1434068_s_at | NR_015519 | AI662270 | expressed sequence AI662270 | 1,24 | 5,95E-05 |
| 1456328_at | NM_001033350 | Bank1 | B-cell scaffold protein with ankyrin repeats 1 | 1,25 | 7,30E-04 |
| 1452405_x_at | --- | A130082M07Rik | RIKEN cDNA A130082M07 gene | 1,25 | 6,17E-03 |
| 1424965_at | NM_134152 | Lpxn | leupaxin | 1,25 | 5,13E-04 |
| 1450792_at | NM_011662 | Tyrobp | TYRO protein tyrosine kinase binding protein | 1,25 | 1,75E-05 |
| 1442082_at | NM_009779 | C3ar1 | complement component 3a receptor 1 | 1,25 | 9,81E-04 |
| 1436171_at | NM_001005508 | Arhgap30 | Rho GTPase activating protein 30 | 1,26 | 6,29E-06 |
| 1428909_at | NR_002860 | A130040M12Rik | RIKEN cDNA A130040M12 gene | 1,26 | 7,79E-04 |
| 1428720_s_at | XM_913918 | 2010309G21Rik /// Igl-C2 /// Igl-C3 | RIKEN cDNA 2010309G21 gene /// immunoglobulin lambda chain, constant region 2 /// immunoglobulin lambda chain, constant region 3 | 1,26 | 2,91E-04 |
| 1450291_s_at | NM_029499 | Ms4a4c | membrane-spanning 4-domains, subfamily A, member 4C | 1,26 | 4,62E-03 |
| 1448485_at | NM_008116 | Ggt1 | gamma-glutamyltransferase 1 | 1,26 | 1,17E-03 |
| 1417267_s_at | NM_024169 | Fkbp11 | FK506 binding protein 11 | 1,26 | 2,32E-06 |
| 1448249_at | NM_010271 | Gpd1 | glycerol-3-phosphate dehydrogenase 1 (soluble) | 1,27 | 4,35E-05 |
| 1427539_a_at | NM_025635 | Zwint | ZW10 interactor | 1,27 | 1,08E-03 |
| 1419105_at | NM_001163504 | Nr1h4 | nuclear receptor subfamily 1, group H, member 4 | 1,27 | 1,45E-04 |
| 1448734_at | NM_001042611 | Cp | ceruloplasmin | 1,27 | 1,43E-02 |
| 1423008_at | NM_007490 | Art2a-ps | ADP-ribosyltransferase 2a, pseudogene | 1,27 | 1,81E-02 |
| 1457253_at | NM_001033235 | Trim40 | tripartite motif-containing 40 | 1,28 | 1,58E-03 |
| 1448449_at | NM_001164107 | Ripk3 | receptor-interacting serine-threonine kinase 3 | 1,28 | 3,64E-04 |
| 1455084_x_at | NM_028230 | Shmt2 | serine hydroxymethyltransferase 2 (mitochondrial) | 1,28 | 2,45E-04 |
| 1418340_at | NM_010185 | Fcer1g | Fc receptor, IgE, high affinity I, gamma polypeptide | 1,28 | 1,53E-05 |
| 1424509_at | NM_026862 | Cd177 | CD177 antigen | 1,28 | 1,27E-06 |
| 1425294_at | NM_029084 | Slamf8 | SLAM family member 8 | 1,29 | 3,18E-04 |
| 1420464_s_at | NM_001166672 | Gm10693 /// Gm14548 /// Lilra6 /// Lilrb3 /// Pira1 /// Pira11 /// Pira2 /// Pira4 /// Pira6 /// Pira7 | predicted pseudogene 10693 /// predicted gene 14548 /// leukocyte immunoglobulin-like receptor, subfamily A (with TM domain), member 6 /// leukocyte immunoglobulin-like receptor, subfamily B (with TM and ITIM domains), member 3 /// paired-Ig-like receptor A1 /// paired-Ig-like receptor A11 /// paired-Ig-like receptor A2 /// paired-Ig-like receptor A4 /// paired-Ig-like receptor A6 /// paired-Ig-like receptor A7 | 1,29 | 4,65E-03 |
| 1428223_at | NM_029662 | Mfsd2a | major facilitator superfamily domain containing 2A | 1,29 | 4,47E-03 |
| 1423944_at | NM_017371 | Hpx | hemopexin | 1,29 | 7,15E-03 |
| 1425137_a_at | NM_010391 | H2-Q10 | histocompatibility 2, Q region locus 10 | 1,29 | 2,98E-04 |
| 1446708_at | NM_001162950 | Hif3a | hypoxia inducible factor 3, alpha subunit | 1,29 | 3,45E-03 |
| 1420699_at | NM_020008 | Clec7a | C-type lectin domain family 7, member a | 1,30 | 3,53E-04 |
| 1447541_s_at | NM_008399 | Itgae | integrin alpha E, epithelial-associated | 1,30 | 3,53E-06 |
| 1452458_s_at | NM_001081406 | Ppil5 | peptidylprolyl isomerase (cyclophilin) like 5 | 1,31 | 2,59E-03 |
| 1452349_x_at | NM_001033450 | Ifi205 /// Mnda | interferon activated gene 205 /// myeloid cell nuclear differentiation antigen | 1,31 | 1,59E-03 |
| 1418191_at | NM_011909 | Usp18 | ubiquitin specific peptidase 18 | 1,32 | 1,17E-03 |
| 1455019_x_at | NM_175451 | Ckap4 | cytoskeleton-associated protein 4 | 1,32 | 1,71E-05 |
| 1440837_at | NM_010389 | H2-Ob | histocompatibility 2, O region beta locus | 1,32 | 1,14E-02 |
| 1437366_at | NM_001033262 | March9 | membrane-associated ring finger (C3HC4) 9 | 1,32 | 3,31E-03 |
| 1436598_at | NM_017480 | Icos | inducible T-cell co-stimulator | 1,32 | 4,04E-05 |
| 1449984_at | NM_009140 | Cxcl2 | chemokine (C-X-C motif) ligand 2 | 1,32 | 4,84E-04 |
| 1418157_at | NM_010151 | Nr2f1 | nuclear receptor subfamily 2, group F, member 1 | 1,32 | 9,78E-04 |
| 1456769_at | NM_028207 | Dusp3 | dual specificity phosphatase 3 (vaccinia virus phosphatase VH1-related) | 1,32 | 2,58E-04 |
| 1449858_at | NM_019388 | Cd86 | CD86 antigen | 1,32 | 1,30E-03 |
| 1423954_at | NM_009778 | C3 | complement component 3 | 1,32 | 5,45E-05 |
| 1425396_a_at | NM_001162432 | Lck | lymphocyte protein tyrosine kinase | 1,33 | 1,01E-04 |
| 1456524_at | NM_178591 | Nrg1 | neuregulin 1 | 1,33 | 7,17E-04 |
| 1448617_at | NM_007651 | Cd53 | CD53 antigen | 1,33 | 1,36E-05 |
| 1425029_a_at | NM_001083341 | Mboat2 | membrane bound O-acyltransferase domain containing 2 | 1,33 | 3,61E-04 |
| 1417381_at | NM_007572 | C1qa | complement component 1, q subcomponent, alpha polypeptide | 1,33 | 1,29E-05 |
| 1424958_at | NM_007592 | Car8 | carbonic anhydrase 8 | 1,34 | 1,74E-03 |
| 1422124_a_at | NM_001111316 | Ptprc | protein tyrosine phosphatase, receptor type, C | 1,34 | 3,97E-05 |
| 1427351_s_at | --- | Igh-6 | immunoglobulin heavy chain 6 (heavy chain of IgM) | 1,34 | 3,70E-04 |
| 1438855_x_at | NM_009396 | Tnfaip2 | tumor necrosis factor, alpha-induced protein 2 | 1,34 | 2,04E-05 |
| 1426670_at | NM_021604 | Agrn | agrin | 1,34 | 8,68E-06 |
| 1449014_at | NM_030717 | Lactb | lactamase, beta | 1,34 | 4,91E-05 |
| 1433434_at | NM_178737 | AW551984 | expressed sequence AW551984 | 1,35 | 4,25E-04 |
| 1425801_x_at | NM_028071 | Cotl1 | coactosin-like 1 (Dictyostelium) | 1,35 | 1,24E-03 |
| 1418021_at | NM_009780 | C4b | complement component 4B (Childo blood group) | 1,35 | 1,79E-06 |
| 1450774_at | NM_033478 | Ly6g6d | lymphocyte antigen 6 complex, locus G6D | 1,35 | 1,23E-03 |
| 1452899_at | NR_028261 | Rian | RNA imprinted and accumulated in nucleus | 1,36 | 7,85E-04 |
| 1440481_at | NM_009283 | Stat1 | signal transducer and activator of transcription 1 | 1,36 | 7,87E-03 |
| 1429947_a_at | NM_001139519 | Zbp1 | Z-DNA binding protein 1 | 1,36 | 1,44E-03 |
| 1417426_at | NM_011157 | Srgn | serglycin | 1,36 | 4,40E-06 |
| 1439380_x_at | NR_003633 | Meg3 | maternally expressed 3 | 1,36 | 3,60E-03 |
| 1429692_s_at | NM_008102 | Gch1 | GTP cyclohydrolase 1 | 1,36 | 9,94E-06 |
| 1439494_at | NM_145551 | Slc5a9 | solute carrier family 5 (sodium/glucose cotransporter), member 9 | 1,37 | 3,34E-04 |
| 1450678_at | NM_008404 | Itgb2 | integrin beta 2 | 1,37 | 3,86E-04 |
| 1422760_at | NM_011061 | Padi4 | peptidyl arginine deiminase, type IV | 1,37 | 2,45E-02 |
| 1422670_at | NM_009708 | Rnd2 | Rho family GTPase 2 | 1,37 | 9,99E-03 |
| 1436838_x_at | NM_028071 | Cotl1 | coactosin-like 1 (Dictyostelium) | 1,37 | 5,10E-06 |
| 1417562_at | NM_007918 | Eif4ebp1 | eukaryotic translation initiation factor 4E binding protein 1 | 1,37 | 1,64E-04 |
| 1418204_s_at | NM_019467 | Aif1 | allograft inflammatory factor 1 | 1,38 | 5,72E-03 |
| 1442270_at | --- | --- | --- | 1,38 | 1,05E-03 |
| 1417793_at | NM_019440 | Irgm2 | immunity-related GTPase family M member 2 | 1,38 | 1,33E-03 |
| 1458161_at | NR_001461 | Kcnq1ot1 | KCNQ1 overlapping transcript 1 | 1,38 | 2,77E-03 |
| 1449461_at | NM_022020 | Rbp7 | retinol binding protein 7, cellular | 1,39 | 4,01E-05 |
| 1418809_at | NM_011089 | Pira2 | paired-Ig-like receptor A2 | 1,39 | 4,88E-04 |
| 1424009_at | NM_001161741 | Reg3d | regenerating islet-derived 3 delta | 1,39 | 3,68E-03 |
| 1454607_s_at | NM_177420 | Psat1 | phosphoserine aminotransferase 1 | 1,39 | 3,46E-03 |
| 1453599_at | NM_001042503 | Trim71 | tripartite motif-containing 71 | 1,39 | 1,18E-03 |
| 1452661_at | NM_011638 | Tfrc | transferrin receptor | 1,39 | 2,26E-06 |
| 1449496_at | NM_025929 | 2010109I03Rik | RIKEN cDNA 2010109I03 gene | 1,39 | 9,18E-05 |
| 1436713_s_at | NR_003633 | Meg3 | maternally expressed 3 | 1,39 | 4,52E-04 |
| 1417413_at | NM_008411 | Cuzd1 | CUB and zona pellucida-like domains 1 | 1,39 | 1,26E-03 |
| 1422247_a_at | NM_009484 | Uty | ubiquitously transcribed tetratricopeptide repeat gene, Y chromosome | 1,39 | 6,25E-04 |
| 1421214_at | NM_001111110 | Cmah | cytidine monophospho-N-acetylneuraminic acid hydroxylase | 1,40 | 2,37E-05 |
| 1425084_at | NM_146167 | Gimap7 | GTPase, IMAP family member 7 | 1,40 | 2,83E-03 |
| 1426208_x_at | NM_009538 | Plagl1 | pleiomorphic adenoma gene-like 1 | 1,40 | 8,71E-05 |
| 1450648_s_at | NM_207105 | H2-Ab1 | histocompatibility 2, class II antigen A, beta 1 | 1,40 | 3,76E-07 |
| 1450245_at | NM_011388 | Slc10a2 | solute carrier family 10, member 2 | 1,40 | 2,75E-03 |
| 1429957_at | NM_027105 | Krtap26-1 | keratin associated protein 26-1 | 1,40 | 3,45E-03 |
| 1456598_at | NM_001031622 | Gm16367 /// Gm16427 /// Gm3106 /// Gm7792 | predicted gene 16367 /// predicted gene 16427 /// predicted gene 3106 /// predicted gene 7792 | 1,40 | 4,88E-03 |
| 1415983_at | NM_008879 | Lcp1 | lymphocyte cytosolic protein 1 | 1,40 | 7,47E-06 |
| 1460218_at | NM_013706 | Cd52 | CD52 antigen | 1,41 | 5,01E-04 |
| 1436778_at | NM_007807 | Cybb | cytochrome b-245, beta polypeptide | 1,41 | 8,79E-05 |
| 1430130_at | NM_027913 | Vwce | von Willebrand factor C and EGF domains | 1,42 | 2,74E-04 |
| 1450424_a_at | NM_010531 | Il18bp | interleukin 18 binding protein | 1,42 | 5,92E-05 |
| 1426539_at | NM_145628 | Usp11 | ubiquitin specific peptidase 11 | 1,42 | 1,75E-03 |
| 1417976_at | NM_007398 | Ada | adenosine deaminase | 1,43 | 8,10E-06 |
| 1419599_s_at | NM_026835 | Ms4a6d | membrane-spanning 4-domains, subfamily A, member 6D | 1,43 | 2,89E-04 |
| 1436317_at | XM_003084445 | LOC100504348 | hypothetical protein LOC100504348 | 1,43 | 1,14E-03 |
| 1424254_at | NM_001112715 | Ifitm1 | interferon induced transmembrane protein 1 | 1,44 | 1,02E-04 |
| 1430897_at | NR_033445 | 4931428L18Rik | RIKEN cDNA 4931428L18 gene | 1,44 | 1,61E-03 |
| 1448162_at | NM_011693 | Vcam1 | vascular cell adhesion molecule 1 | 1,44 | 1,47E-04 |
| 1419593_at | NM_015764 | Greb1 | gene regulated by estrogen in breast cancer protein | 1,44 | 6,02E-05 |
| 1434976_x_at | NM_007918 | Eif4ebp1 | eukaryotic translation initiation factor 4E binding protein 1 | 1,44 | 7,01E-04 |
| 1460245_at | NM_010654 | Klrd1 | killer cell lectin-like receptor, subfamily D, member 1 | 1,44 | 1,83E-03 |
| 1416626_at | NM_011107 | Pla2g1b | phospholipase A2, group IB, pancreas | 1,44 | 1,26E-04 |
| 1426161_at | --- | --- | --- | 1,45 | 1,45E-04 |
| 1419907_s_at | NM_001160215 | Fcrla | Fc receptor-like A | 1,45 | 1,22E-04 |
| 1435477_s_at | NM_001077189 | Fcgr2b | Fc receptor, IgG, low affinity IIb | 1,46 | 9,93E-04 |
| 1425854_x_at | XM_977361 | LOC665506 | similar to T-cell receptor beta-2 chain C region | 1,46 | 5,50E-03 |
| 1430979_a_at | NM_011563 | Prdx2 | peroxiredoxin 2 | 1,46 | 1,52E-04 |
| 1451951_at | --- | Gm16939 | predicted gene, 16939 | 1,46 | 1,65E-04 |
| 1449451_at | NM_025867 | Serpinb11 | serine (or cysteine) peptidase inhibitor, clade B (ovalbumin), member 11 | 1,46 | 1,77E-03 |
| 1451064_a_at | NM_177420 | Psat1 | phosphoserine aminotransferase 1 | 1,46 | 1,99E-03 |
| 1460407_at | NM_019866 | Spib | Spi-B transcription factor (Spi-1/PU.1 related) | 1,46 | 2,12E-03 |
| 1443746_x_at | NM_016779 | Dmp1 | dentin matrix protein 1 | 1,47 | 5,90E-05 |
| 1425225_at | NM_144559 | Fcgr4 | Fc receptor, IgG, low affinity IV | 1,47 | 1,79E-05 |
| 1441930_x_at | NM_012037 | Vat1 | Vesicle amine transport protein 1 homolog (T californica) | 1,47 | 2,41E-06 |
| 1455269_a_at | NM_009898 | Coro1a | coronin, actin binding protein 1A | 1,47 | 2,10E-03 |
| 1419744_at | NM_010388 | H2-DMb2 | histocompatibility 2, class II, locus Mb2 | 1,47 | 5,45E-03 |
| 1455241_at | NM_172295 | BC037703 | cDNA sequence BC037703 | 1,47 | 4,29E-05 |
| 1417995_at | NM_008979 | Ptpn22 | protein tyrosine phosphatase, non-receptor type 22 (lymphoid) | 1,47 | 1,80E-04 |
| 1456064_at | NR_033498 | AI504432 | expressed sequence AI504432 | 1,48 | 4,22E-05 |
| 1416295_a_at | NM_013563 | Il2rg | interleukin 2 receptor, gamma chain | 1,48 | 3,62E-04 |
| 1423226_at | NM_007641 | Ms4a1 | membrane-spanning 4-domains, subfamily A, member 1 | 1,48 | 3,80E-05 |
| 1452535_at | --- | Igh-6 | Immunoglobulin heavy chain 6 (heavy chain of IgM) | 1,48 | 2,53E-06 |
| 1454995_at | NM_026993 | Ddah1 | dimethylarginine dimethylaminohydrolase 1 | 1,48 | 8,96E-06 |
| 1435036_at | NM_001081169 | Aspg | asparaginase homolog (S. cerevisiae) | 1,49 | 2,52E-06 |
| 1449988_at | NM_008376 | Gimap1 | GTPase, IMAP family member 1 | 1,49 | 1,46E-03 |
| 1417561_at | NM_001110009 | Apoc1 | apolipoprotein C-I | 1,49 | 3,75E-05 |
| 1455400_at | NM_026993 | Ddah1 | dimethylarginine dimethylaminohydrolase 1 | 1,49 | 3,95E-04 |
| 1423285_at | NM_001198835 | Coch | coagulation factor C homolog (Limulus polyphemus) | 1,50 | 3,17E-04 |
| 1434067_at | NR_015519 | AI662270 | expressed sequence AI662270 | 1,50 | 1,93E-03 |
| 1429298_at | NM_026993 | Ddah1 | dimethylarginine dimethylaminohydrolase 1 | 1,50 | 4,04E-05 |
| 1446834_at | NM_009982 | Ctsc | Cathepsin C | 1,50 | 2,27E-03 |
| 1450912_at | NM_007641 | Ms4a1 | membrane-spanning 4-domains, subfamily A, member 1 | 1,50 | 1,69E-03 |
| 1416246_a_at | NM_009898 | Coro1a | coronin, actin binding protein 1A | 1,51 | 3,28E-04 |
| 1419431_at | NM_007950 | Ereg | epiregulin | 1,51 | 1,43E-04 |
| 1425951_a_at | NM_001190320 | Clec4n | C-type lectin domain family 4, member n | 1,51 | 1,58E-04 |
| 1427102_at | NM_011410 | Slfn4 | schlafen 4 | 1,51 | 8,23E-06 |
| 1444619_x_at | NM_010724 | Psmb8 | proteasome (prosome, macropain) subunit, beta type 8 (large multifunctional peptidase 7) | 1,51 | 1,08E-02 |
| 1417090_at | NM_009037 | Rcn1 | reticulocalbin 1 | 1,51 | 1,50E-05 |
| 1436649_at | NM_011771 | Ikzf3 | IKAROS family zinc finger 3 | 1,51 | 1,10E-03 |
| 1417483_at | NM_001159394 | Nfkbiz | nuclear factor of kappa light polypeptide gene enhancer in B-cells inhibitor, zeta | 1,52 | 7,36E-05 |
| 1426002_a_at | NM_009863 | Cdc7 | cell division cycle 7 (S. cerevisiae) | 1,52 | 4,46E-04 |
| 1440245_at | NM_001081291 | Ccdc88b | coiled-coil domain containing 88B | 1,52 | 6,38E-03 |
| 1448783_at | NM_021291 | Slc7a9 | solute carrier family 7 (cationic amino acid transporter, y+ system), member 9 | 1,53 | 1,31E-03 |
| 1415805_at | NM_025469 | Clps | colipase, pancreatic | 1,53 | 3,26E-06 |
| 1417597_at | NM_007642 | Cd28 | CD28 antigen | 1,53 | 1,21E-04 |
| 1425993_a_at | NM_013559 | Hsph1 | heat shock 105kDa/110kDa protein 1 | 1,54 | 2,55E-05 |
| 1450271_at | NM_009184 | Ptk6 | PTK6 protein tyrosine kinase 6 | 1,54 | 1,66E-03 |
| 1439568_at | NM_015764 | Greb1 | gene regulated by estrogen in breast cancer protein | 1,54 | 8,66E-04 |
| 1430523_s_at | --- | Igl-V1 | immunoglobulin lambda chain, variable 1 | 1,55 | 4,56E-05 |
| 1422812_at | NM_030712 | Cxcr6 | chemokine (C-X-C motif) receptor 6 | 1,55 | 8,25E-04 |
| 1418536_at | NM_001198560 | H2-Q7 | histocompatibility 2, Q region locus 7 | 1,55 | 9,37E-04 |
| 1428351_at | NM_026447 | Ppm1m | protein phosphatase 1M | 1,55 | 3,06E-03 |
| 1452348_s_at | NM_001033450 | Ifi204 /// Ifi205 /// Mnda /// Mndal | interferon activated gene 204 /// interferon activated gene 205 /// myeloid cell nuclear differentiation antigen /// myeloid nuclear differentiation antigen like | 1,55 | 1,43E-04 |
| 1422868_s_at | NM_010266 | Gda | guanine deaminase | 1,56 | 7,93E-05 |
| 1443745_s_at | NM_016779 | Dmp1 | dentin matrix protein 1 | 1,56 | 2,13E-04 |
| 1430802_at | NM_023124 | H2-Q8 | histocompatibility 2, Q region locus 8 | 1,56 | 4,11E-03 |
| 1436576_at | NM_175449 | Fam26f | family with sequence similarity 26, member F | 1,56 | 1,02E-04 |
| 1418392_a_at | NM_018734 | Gbp3 | guanylate binding protein 3 | 1,57 | 7,30E-04 |
| 1424727_at | NM_009917 | Ccr5 | chemokine (C-C motif) receptor 5 | 1,57 | 4,02E-03 |
| 1456471_x_at | NM_016966 | Gm6756 /// Phgdh | 3-phosphoglycerate dehydrogenase pseudogene /// 3-phosphoglycerate dehydrogenase | 1,57 | 4,39E-05 |
| 1432591_at | NM_021362 | Pappa | pregnancy-associated plasma protein A | 1,57 | 1,19E-03 |
| 1437939_s_at | NM_009982 | Ctsc | cathepsin C | 1,57 | 1,35E-04 |
| 1442029_at | NR_001461 | Kcnq1ot1 | KCNQ1 overlapping transcript 1 | 1,57 | 7,33E-03 |
| 1418930_at | NM_021274 | Cxcl10 | chemokine (C-X-C motif) ligand 10 | 1,58 | 3,66E-03 |
| 1436905_x_at | NM_010686 | Laptm5 | lysosomal-associated protein transmembrane 5 | 1,58 | 8,80E-06 |
| 1439727_at | NM_207208 | Clca6 | chloride channel calcium activated 6 | 1,58 | 6,16E-05 |
| 1419473_a_at | NM_031161 | Cck | cholecystokinin | 1,59 | 9,13E-05 |
| 1424931_s_at | --- | Igl-C1 /// Igl-V1 /// LOC433053 | immunoglobulin lambda chain, constant region 1 /// immunoglobulin lambda chain, variable 1 /// similar to Ig lambda-1 chain C region | 1,59 | 7,45E-06 |
| 1460167_at | NM_001127338 | Aldh7a1 | aldehyde dehydrogenase family 7, member A1 | 1,60 | 4,79E-05 |
| 1450886_at | NM_010353 | Gsg2 | germ cell-specific gene 2 | 1,60 | 7,02E-04 |
| 1422188_s_at | --- | Tcrg-V2 /// Tcrg-V3 | T-cell receptor gamma, variable 2 /// T-cell receptor gamma, variable 3 | 1,61 | 1,14E-03 |
| 1419598_at | NM_026835 | Ms4a6d | membrane-spanning 4-domains, subfamily A, member 6D | 1,61 | 2,12E-06 |
| 1418571_at | NM_001161746 | Tnfrsf12a | tumor necrosis factor receptor superfamily, member 12a | 1,61 | 5,78E-05 |
| 1416632_at | NM_008615 | Me1 | malic enzyme 1, NADP(+)-dependent, cytosolic | 1,61 | 8,81E-05 |
| 1416120_at | NM_009104 | Rrm2 | ribonucleotide reductase M2 | 1,62 | 2,03E-03 |
| 1419136_at | NM_134066 | Akr1c18 | aldo-keto reductase family 1, member C18 | 1,62 | 2,77E-05 |
| 1457904_at | NM_007592 | Car8 | carbonic anhydrase 8 | 1,62 | 4,74E-03 |
| 1419178_at | NM_009850 | Cd3g | CD3 antigen, gamma polypeptide | 1,62 | 1,43E-03 |
| 1428420_a_at | NM_028807 | 1200009I06Rik | RIKEN cDNA 1200009I06 gene | 1,62 | 1,58E-03 |
| 1452205_x_at | XM_977361 | Gm6273 /// LOC381765 /// LOC665506 /// Tcrb-J | predicted gene 6273 /// similar to T cell antigen receptor /// similar to T-cell receptor beta-2 chain C region /// T-cell receptor beta, joining region | 1,63 | 1,90E-04 |
| 1432976_at | NM_001079865 | Ces2f | carboxylesterase 2F | 1,63 | 2,33E-03 |
| 1449175_at | NM_008152 | Gpr65 | G-protein coupled receptor 65 | 1,63 | 4,35E-03 |
| 1452815_at | NM_172435 | P2ry10 | purinergic receptor P2Y, G-protein coupled 10 | 1,63 | 2,13E-03 |
| 1442291_at | NM_020028 | Lpar2 | lysophosphatidic acid receptor 2 | 1,63 | 5,58E-03 |
| 1422411_s_at | NM_001012766 | BC151093 /// Ear1 /// Ear12 /// Ear2 /// Ear3 | cDNA sequence BC151093 /// eosinophil-associated, ribonuclease A family, member 1 /// eosinophil-associated, ribonuclease A family, member 12 /// eosinophil-associated, ribonuclease A family, member 2 /// eosinophil-associated, ribonuclease A family, member 3 | 1,63 | 1,90E-03 |
| 1457614_at | NM_174850 | Micall2 | MICAL-like 2 | 1,63 | 2,23E-03 |
| 1435290_x_at | NM_010378 | H2-Aa | histocompatibility 2, class II antigen A, alpha | 1,64 | 2,51E-04 |
| 1437270_a_at | NM_019952 | Clcf1 | cardiotrophin-like cytokine factor 1 | 1,64 | 5,01E-04 |
| 1449133_at | NM_009264 | Sprr1a | small proline-rich protein 1A | 1,64 | 5,21E-05 |
| 1450033_a_at | NM_009283 | Stat1 | signal transducer and activator of transcription 1 | 1,64 | 8,34E-07 |
| 1421178_at | NM_001162368 | Mgat4c | mannosyl (alpha-1,3-)-glycoprotein beta-1,4-N-acetylglucosaminyltransferase, isozyme C (putative) | 1,64 | 9,64E-05 |
| 1434437_x_at | NM_009104 | Rrm2 | ribonucleotide reductase M2 | 1,65 | 4,73E-06 |
| 1452614_at | NM_001142959 | Bcl2l15 | BCLl2-like 15 | 1,65 | 6,75E-05 |
| 1417640_at | NM_008339 | Cd79b | CD79B antigen | 1,65 | 3,87E-06 |
| 1451567_a_at | NM_001045481 | Ifi203 /// LOC100044071 | interferon activated gene 203 /// interferon-activable protein 203-like | 1,65 | 2,90E-03 |
| 1418739_at | NM_013731 | Sgk2 | serum/glucocorticoid regulated kinase 2 | 1,65 | 3,03E-05 |
| 1428735_at | NM_001033122 | Cd69 | CD69 antigen | 1,65 | 1,78E-03 |
| 1420569_at | NM_007689 | Chad | chondroadherin | 1,66 | 9,70E-06 |
| 1423566_a_at | NM_013559 | Hsph1 | heat shock 105kDa/110kDa protein 1 | 1,66 | 1,30E-05 |
| 1454714_x_at | NM_016966 | Phgdh | 3-phosphoglycerate dehydrogenase | 1,67 | 1,37E-03 |
| 1424067_at | NM_010493 | Icam1 | intercellular adhesion molecule 1 | 1,67 | 2,24E-06 |
| 1416002_x_at | NM_028071 | Cotl1 | coactosin-like 1 (Dictyostelium) | 1,67 | 1,15E-05 |
| 1435415_x_at | NM_010807 | Marcksl1 | MARCKS-like 1 | 1,67 | 6,41E-04 |
| 1451602_at | NM_026998 | Snx6 | sorting nexin 6 | 1,67 | 3,13E-04 |
| 1430307_a_at | NM_008615 | Me1 | malic enzyme 1, NADP(+)-dependent, cytosolic | 1,67 | 1,80E-05 |
| 1426200_at | --- | Gm1499 /// Gm1524 | predicted gene 1499 /// predicted gene 1524 | 1,68 | 1,48E-04 |
| 1429184_at | NM_001039160 | Gvin1 | GTPase, very large interferon inducible 1 | 1,68 | 1,10E-04 |
| 1448612_at | NM_018754 | Sfn | stratifin | 1,68 | 2,39E-06 |
| 1417256_at | NM_008607 | Mmp13 | matrix metallopeptidase 13 | 1,68 | 1,01E-04 |
| 1443392_at | NM_001001445 | Trpv1 | transient receptor potential cation channel, subfamily V, member 1 | 1,69 | 1,50E-05 |
| 1446368_at | --- | 9130221J18Rik | RIKEN cDNA 9130221J18 gene | 1,69 | 2,73E-04 |
| 1417620_at | NM_009008 | Rac2 | RAS-related C3 botulinum substrate 2 | 1,71 | 5,46E-06 |
| 1427860_at | --- | --- | --- | 1,71 | 1,84E-04 |
| 1416957_at | NM_011136 | Pou2af1 | POU domain, class 2, associating factor 1 | 1,72 | 4,65E-06 |
| 1434458_at | NM_008046 | Fst | follistatin | 1,72 | 2,00E-04 |
| 1456584_x_at | NM_016966 | Phgdh | 3-phosphoglycerate dehydrogenase | 1,72 | 3,89E-04 |
| 1448226_at | NM_009104 | Rrm2 | ribonucleotide reductase M2 | 1,72 | 3,11E-04 |
| 1437811_x_at | --- | --- | --- | 1,73 | 1,37E-03 |
| 1421551_s_at | NM_008327 | Ifi202b | interferon activated gene 202B | 1,74 | 6,93E-03 |
| 1424975_at | NM_145581 | Siglec5 | sialic acid binding Ig-like lectin 5 | 1,74 | 4,09E-03 |
| 1429954_at | NM_153197 | Clec4a3 | C-type lectin domain family 4, member a3 | 1,74 | 1,74E-03 |
| 1418572_x_at | NM_001161746 | Tnfrsf12a | tumor necrosis factor receptor superfamily, member 12a | 1,74 | 3,99E-07 |
| 1457231_at | --- | --- | --- | 1,75 | 3,38E-03 |
| 1442812_at | NM_001034888 | Gm10482 | predicted gene 10482 | 1,75 | 4,14E-04 |
| 1435640_x_at | NR_002860 | A130040M12Rik | RIKEN cDNA A130040M12 gene | 1,75 | 5,81E-03 |
| 1426174_s_at | XM_001472541 | Igh-3 /// Ighg | immunoglobulin heavy chain 3 (serum IgG2b) /// Immunoglobulin heavy chain (gamma polypeptide) | 1,75 | 2,97E-04 |
| 1438941_x_at | NM_028779 | Ampd2 | adenosine monophosphate deaminase 2 | 1,75 | 3,21E-02 |
| 1454854_at | NM_178933 | Ostb | organic solute transporter beta | 1,75 | 8,32E-06 |
| 1425477_x_at | NM_207105 | H2-Ab1 | histocompatibility 2, class II antigen A, beta 1 | 1,76 | 1,47E-07 |
| 1417177_at | NM_016905 | Galk1 | galactokinase 1 | 1,76 | 5,36E-06 |
| 1449526_a_at | NM_024228 | Gdpd3 | glycerophosphodiester phosphodiesterase domain containing 3 | 1,76 | 1,92E-03 |
| 1449254_at | NM_009263 | Spp1 | secreted phosphoprotein 1 | 1,78 | 4,57E-05 |
| 1421354_at | NM_008926 | Prkg2 | protein kinase, cGMP-dependent, type II | 1,78 | 2,16E-05 |
| 1452677_at | NM_027869 | Pnpt1 | polyribonucleotide nucleotidyltransferase 1 | 1,79 | 3,26E-03 |
| 1422317_a_at | NM_001025602 | Il1rl1 | interleukin 1 receptor-like 1 | 1,81 | 3,57E-05 |
| 1428947_at | NM_027222 | 2010001M09Rik | RIKEN cDNA 2010001M09 gene | 1,81 | 8,80E-06 |
| 1416576_at | NM_007707 | Socs3 | suppressor of cytokine signaling 3 | 1,81 | 5,36E-06 |
| 1438439_at | NM_173398 | Gpr171 | G protein-coupled receptor 171 | 1,82 | 4,12E-04 |
| 1449478_at | NM_010810 | Mmp7 | matrix metallopeptidase 7 | 1,82 | 1,40E-04 |
| 1450562_at | NM_008530 | Ly6f | lymphocyte antigen 6 complex, locus F | 1,82 | 6,30E-03 |
| 1425519_a_at | NM_001042605 | Cd74 | CD74 antigen (invariant polypeptide of major histocompatibility complex, class II antigen-associated) | 1,83 | 2,30E-05 |
| 1432750_at | NM_177747 | Zfp711 | zinc finger protein 711 | 1,83 | 1,04E-04 |
| 1416783_at | NM_009311 | Tac1 | tachykinin 1 | 1,84 | 1,92E-06 |
| 1452463_x_at | --- | Gm10883 | predicted gene 10883 | 1,84 | 3,87E-06 |
| 1418777_at | NM_009138 | Ccl25 | chemokine (C-C motif) ligand 25 | 1,84 | 9,59E-05 |
| 1427577_x_at | --- | Gm7202 /// Igk-C /// Igk-V19-14 /// Igk-V28 /// Igkv6-25 | predicted gene 7202 /// immunoglobulin kappa chain, constant region /// immunoglobulin kappa chain variable 19 (V19)-14 /// immunoglobulin kappa chain variable 28 (V28) /// immunoglobulin kappa chain variable 6-25 | 1,85 | 1,01E-03 |
| 1424631_a_at | XM_001472541 | Ighg | Immunoglobulin heavy chain (gamma polypeptide) | 1,85 | 5,41E-06 |
| 1448859_at | NM_018866 | Cxcl13 | chemokine (C-X-C motif) ligand 13 | 1,85 | 8,41E-05 |
| 1415922_s_at | NM_010807 | Marcksl1 | MARCKS-like 1 | 1,86 | 2,00E-04 |
| 1416646_at | NM_007423 | Afp | alpha fetoprotein | 1,86 | 7,83E-03 |
| 1447927_at | NM_001039646 | Gbp10 /// Mpa2l | guanylate-binding protein 10 /// macrophage activation 2 like | 1,86 | 1,23E-05 |
| 1438165_x_at | NM_012037 | Vat1 | vesicle amine transport protein 1 homolog (T californica) | 1,86 | 1,53E-04 |
| 1423680_at | NM_146094 | Fads1 | fatty acid desaturase 1 | 1,87 | 3,17E-06 |
| 1420358_at | NM_010671 | Krtap13 | keratin associated protein 13 | 1,87 | 2,18E-05 |
| 1443686_at | NM_010388 | H2-DMb2 | histocompatibility 2, class II, locus Mb2 | 1,87 | 9,98E-05 |
| 1438148_at | NM_203320 | Cxcl3 | chemokine (C-X-C motif) ligand 3 | 1,89 | 1,61E-03 |
| 1426178_at | --- | Igk | Immunoglobulin kappa chain complex | 1,89 | 9,25E-05 |
| 1454242_at | NM_027173 | 2310079G19Rik | RIKEN cDNA 2310079G19 gene | 1,89 | 1,09E-04 |
| 1417300_at | NM_133888 | Smpdl3b | sphingomyelin phosphodiesterase, acid-like 3B | 1,90 | 2,74E-04 |
| 1427576_at | --- | Gm7202 /// Igk-C /// Igk-V19-14 /// Igk-V28 /// Igkv6-25 | predicted gene 7202 /// immunoglobulin kappa chain, constant region /// immunoglobulin kappa chain variable 19 (V19)-14 /// immunoglobulin kappa chain variable 28 (V28) /// immunoglobulin kappa chain variable 6-25 | 1,90 | 5,92E-04 |
| 1451965_at | --- | Gm7202 /// Igk-V19-14 /// Igk-V28 /// Igkv6-25 | predicted gene 7202 /// immunoglobulin kappa chain variable 19 (V19)-14 /// immunoglobulin kappa chain variable 28 (V28) /// immunoglobulin kappa chain variable 6-25 | 1,90 | 6,27E-05 |
| 1420499_at | NM_008102 | Gch1 | GTP cyclohydrolase 1 | 1,90 | 2,03E-07 |
| 1456907_at | NM_008599 | Cxcl9 | chemokine (C-X-C motif) ligand 9 | 1,90 | 7,02E-03 |
| 1435560_at | NM_008400 | Itgal | integrin alpha L | 1,92 | 8,47E-04 |
| 1438612_a_at | NM_025469 | Clps | colipase, pancreatic | 1,92 | 7,92E-07 |
| 1435627_x_at | NM_010807 | Marcksl1 | MARCKS-like 1 | 1,92 | 1,08E-03 |
| 1422749_at | NM_023463 | Ly6g6c | lymphocyte antigen 6 complex, locus G6C | 1,93 | 1,01E-06 |
| 1438676_at | NM_194336 | Mpa2l | macrophage activation 2 like | 1,93 | 1,81E-03 |
| 1448025_at | NM_001002898 | LOC100038947 /// Sirpb1a /// Sirpb1b | signal-regulatory protein beta 1-like /// signal-regulatory protein beta 1A /// signal-regulatory protein beta 1B | 1,93 | 4,50E-05 |
| 1419004_s_at | NM_007534 | Bcl2a1a /// Bcl2a1b /// Bcl2a1d | B-cell leukemia/lymphoma 2 related protein A1a /// B-cell leukemia/lymphoma 2 related protein A1b /// B-cell leukemia/lymphoma 2 related protein A1d | 1,94 | 2,71E-06 |
| 1422400_a_at | NM_001177524 | Gml /// Hemt1 | GPI anchored molecule like protein /// hematopoietic cell transcript 1 | 1,94 | 2,99E-03 |
| 1436434_at | NM_177733 | E2f2 | E2F transcription factor 2 | 1,94 | 2,58E-04 |
| 1417851_at | NM_018866 | Cxcl13 | chemokine (C-X-C motif) ligand 13 | 1,95 | 4,20E-04 |
| 1430128_a_at | NM_139292 | Reep6 | receptor accessory protein 6 | 1,96 | 5,28E-07 |
| 1422962_a_at | NM_010724 | Psmb8 | proteasome (prosome, macropain) subunit, beta type 8 (large multifunctional peptidase 7) | 1,96 | 2,70E-06 |
| 1452840_at | NM_001145198 | 1500009L16Rik | RIKEN cDNA 1500009L16 gene | 1,97 | 2,40E-04 |
| 1450387_s_at | NM_001177602 | Ak4 | adenylate kinase 4 | 1,97 | 1,60E-04 |
| 1437176_at | NM_001033207 | Nlrc5 | NLR family, CARD domain containing 5 | 1,98 | 2,14E-05 |
| 1451721_a_at | NM_207105 | H2-Ab1 | histocompatibility 2, class II antigen A, beta 1 | 1,98 | 6,75E-07 |
| 1450696_at | NM_013585 | Psmb9 | proteasome (prosome, macropain) subunit, beta type 9 (large multifunctional peptidase 2) | 1,99 | 4,11E-06 |
| 1448022_at | --- | --- | --- | 1,99 | 3,87E-06 |
| 1438223_at | --- | --- | --- | 2,00 | 1,96E-04 |
| 1429608_at | NM_026945 | Adh6a | alcohol dehydrogenase 6A (class V) | 2,00 | 3,01E-03 |
| 1443687_x_at | NM_010388 | H2-DMb2 | histocompatibility 2, class II, locus Mb2 | 2,00 | 1,22E-05 |
| 1429104_at | NM_172397 | Limd2 | LIM domain containing 2 | 2,01 | 2,87E-06 |
| 1419399_at | NM_001163457 | Mttp | microsomal triglyceride transfer protein | 2,02 | 2,68E-04 |
| 1447766_x_at | NM_172397 | Limd2 | LIM domain containing 2 | 2,02 | 2,66E-03 |
| 1417837_at | NM_009434 | Phlda2 | pleckstrin homology-like domain, family A, member 2 | 2,03 | 6,74E-06 |
| 1455790_at | NM_177733 | E2f2 | E2F transcription factor 2 | 2,03 | 2,95E-06 |
| 1451963_at | --- | Igh-VJ558 /// LOC675759 | immunoglobulin heavy chain (J558 family) /// ig heavy chain V-III region J606-like | 2,04 | 1,99E-05 |
| 1421041_s_at | NM_008181 | Gm3776 /// Gsta1 /// Gsta2 | predicted gene 3776 /// glutathione S-transferase, alpha 1 (Ya) /// glutathione S-transferase, alpha 2 (Yc2) | 2,04 | 2,63E-04 |
| 1417714_x_at | NM_001083955 | Hba-a1 /// Hba-a2 | hemoglobin alpha, adult chain 1 /// hemoglobin alpha, adult chain 2 | 2,04 | 3,11E-06 |
| 1418293_at | NM_008332 | Ifit2 | interferon-induced protein with tetratricopeptide repeats 2 | 2,07 | 6,39E-04 |
| 1419684_at | NM_021443 | Ccl8 | chemokine (C-C motif) ligand 8 | 2,08 | 2,14E-03 |
| 1419100_at | NM_009252 | Serpina3n | serine (or cysteine) peptidase inhibitor, clade A, member 3N | 2,08 | 7,77E-05 |
| 1418240_at | NM_010260 | Gbp2 | guanylate binding protein 2 | 2,10 | 8,60E-05 |
| 1425738_at | --- | --- | --- | 2,10 | 2,02E-06 |
| 1437226_x_at | NM_010807 | Marcksl1 | MARCKS-like 1 | 2,12 | 1,95E-05 |
| 1418776_at | NM_029509 | Gbp8 | guanylate-binding protein 8 | 2,12 | 2,08E-04 |
| 1438858_x_at | NM_010378 | H2-Aa | histocompatibility 2, class II antigen A, alpha | 2,13 | 1,43E-04 |
| 1427837_at | --- | Igk-V32 | Immunoglobulin kappa chain variable 32 (V32) | 2,13 | 7,13E-06 |
| 1435906_x_at | NM_010260 | Gbp2 | guanylate binding protein 2 | 2,13 | 4,73E-07 |
| 1425385_a_at | --- | Igh-6 | Immunoglobulin heavy chain 6 (heavy chain of IgM) | 2,13 | 2,20E-04 |
| 1426168_a_at | --- | A130082M07Rik /// Tcra | RIKEN cDNA A130082M07 gene /// T-cell receptor alpha chain | 2,16 | 5,99E-03 |
| 1448756_at | NM_009114 | S100a9 | S100 calcium binding protein A9 (calgranulin B) | 2,17 | 2,84E-06 |
| 1421830_at | NM_001177602 | Ak4 | adenylate kinase 4 | 2,18 | 6,18E-04 |
| 1449495_at | NM_011259 | Reg3a | regenerating islet-derived 3 alpha | 2,18 | 4,80E-04 |
| 1437621_x_at | NM_016966 | Phgdh | 3-phosphoglycerate dehydrogenase | 2,21 | 1,22E-06 |
| 1417765_a_at | NM_001110505 | Amy1 | amylase 1, salivary | 2,22 | 2,90E-04 |
| 1422527_at | NM_010386 | H2-DMa | histocompatibility 2, class II, locus DMa | 2,22 | 3,95E-05 |
| 1452431_s_at | NM_010378 | H2-Aa | histocompatibility 2, class II antigen A, alpha | 2,24 | 2,41E-04 |
| 1450582_at | NM_010393 | H2-Q5 | histocompatibility 2, Q region locus 5 | 2,24 | 5,15E-03 |
| 1426657_s_at | NM_016966 | Gm8096 /// Phgdh | 3-phosphoglycerate dehydrogenase pseudogene /// 3-phosphoglycerate dehydrogenase | 2,25 | 8,39E-05 |
| 1424037_at | NM_146125 | Itpka | inositol 1,4,5-trisphosphate 3-kinase A | 2,25 | 6,35E-05 |
| 1424882_a_at | NM_027289 | Nt5dc2 | 5'-nucleotidase domain containing 2 | 2,25 | 6,38E-06 |
| 1424923_at | NM_009251 | Serpina3g | serine (or cysteine) peptidase inhibitor, clade A, member 3G | 2,26 | 1,67E-03 |
| 1417025_at | NM_010382 | H2-Eb1 | histocompatibility 2, class II antigen E beta | 2,27 | 4,19E-05 |
| 1449191_at | NM_138684 | Wfdc12 | WAP four-disulfide core domain 12 | 2,28 | 5,87E-05 |
| 1450826_a_at | NM_011315 | Saa3 | serum amyloid A 3 | 2,29 | 1,11E-03 |
| 1450128_at | NM_001082531 | Pla2g2a | phospholipase A2, group IIA (platelets, synovial fluid) | 2,29 | 3,50E-03 |
| 1439831_at | --- | --- | --- | 2,30 | 1,49E-04 |
| 1422137_at | NM_025777 | Duoxa2 | dual oxidase maturation factor 2 | 2,31 | 6,05E-05 |
| 1460197_a_at | NM_054098 | Steap4 | STEAP family member 4 | 2,31 | 3,83E-04 |
| 1449452_a_at | NM_025989 | Gp2 | glycoprotein 2 (zymogen granule membrane) | 2,32 | 1,37E-05 |
| 1449580_s_at | NM_010387 | H2-DMb1 /// H2-DMb2 | histocompatibility 2, class II, locus Mb1 /// histocompatibility 2, class II, locus Mb2 | 2,32 | 4,77E-05 |
| 1422891_at | NM_010381 | H2-Ea-ps /// LOC100504404 | histocompatibility 2, class II antigen E alpha, pseudogene /// h-2 class II histocompatibility antigen, E-K alpha chain-like | 2,33 | 1,15E-05 |
| 1427851_x_at | --- | AB069917 /// Ighv1-72 /// LOC631518 | cDNA sequence AB069917 /// immunoglobulin heavy variable V1-72 /// ig heavy chain V region VH558 B4-like | 2,35 | 2,14E-05 |
| 1419167_at | NM_009475 | Prap1 | proline-rich acidic protein 1 | 2,36 | 4,38E-04 |
| 1419294_at | NM_025956 | 1700011H14Rik | RIKEN cDNA 1700011H14 gene | 2,38 | 4,74E-04 |
| 1419209_at | NM_008176 | Cxcl1 | chemokine (C-X-C motif) ligand 1 | 2,39 | 4,74E-06 |
| 1417141_at | NM_018738 | Igtp | interferon gamma induced GTPase | 2,39 | 1,81E-05 |
| 1425078_x_at | NM_001037909 | C130026I21Rik /// LOC100041885 | RIKEN cDNA C130026I21 gene /// sp110 nuclear body protein-like | 2,41 | 1,16E-04 |
| 1451593_at | NM_001001892 | H2-K1 | Histocompatibility 2, K1, K region | 2,41 | 1,33E-04 |
| 1419762_at | NM_023137 | Ubd | ubiquitin D | 2,41 | 1,31E-04 |
| 1428952_at | NM_001081070 | Pdia2 | protein disulfide isomerase associated 2 | 2,43 | 3,30E-03 |
| 1449989_at | NM_008571 | Mcpt2 | mast cell protease 2 | 2,43 | 1,61E-08 |
| 1438555_x_at | NM_080457 | Muc4 | mucin 4 | 2,47 | 3,30E-04 |
| 1419498_at | NM_025655 | Tmigd1 | transmembrane and immunoglobulin domain containing 1 | 2,48 | 1,71E-06 |
| 1439622_at | NM_178045 | Rassf4 | Ras association (RalGDS/AF-6) domain family member 4 | 2,49 | 9,42E-04 |
| 1418638_at | NM_010387 | H2-DMb1 /// H2-DMb2 | histocompatibility 2, class II, locus Mb1 /// histocompatibility 2, class II, locus Mb2 | 2,52 | 1,81E-04 |
| 1451481_s_at | NM_145932 | Osta | organic solute transporter alpha | 2,54 | 2,30E-04 |
| 1443783_x_at | NM_010378 | H2-Aa | histocompatibility 2, class II antigen A, alpha | 2,54 | 2,56E-03 |
| 1417898_a_at | NM_010370 | Gzma | granzyme A | 2,55 | 2,12E-03 |
| 1417802_at | NM_001164210 | 1110032A04Rik | RIKEN cDNA 1110032A04 gene | 2,57 | 1,20E-03 |
| 1452077_at | NM_012008 | Ddx3y | DEAD (Asp-Glu-Ala-Asp) box polypeptide 3, Y-linked | 2,59 | 6,16E-04 |
| 1427482_a_at | NM_007592 | Car8 | carbonic anhydrase 8 | 2,60 | 2,22E-04 |
| 1451916_s_at | NM_001024134 | Trim15 | tripartite motif-containing 15 | 2,60 | 1,07E-03 |
| 1457666_s_at | NM_008327 | Ifi202b | interferon activated gene 202B | 2,61 | 3,33E-06 |
| 1426598_at | NM_009484 | Uty | ubiquitously transcribed tetratricopeptide repeat gene, Y chromosome | 2,62 | 4,87E-05 |
| 1435792_at | NM_033616 | Csprs /// Gm7592 | component of Sp100-rs /// predicted gene 7592 | 2,66 | 5,50E-08 |
| 1425900_at | NM_145419 | Hkdc1 | hexokinase domain containing 1 | 2,67 | 9,75E-06 |
| 1427751_a_at | NM_001174099 | Krt36 | keratin 36 | 2,68 | 4,39E-05 |
| 1418155_at | NM_001033621 | Myot | myotilin | 2,69 | 3,04E-05 |
| 1423467_at | NM_021718 | Ms4a4b | membrane-spanning 4-domains, subfamily A, member 4B | 2,70 | 1,92E-04 |
| 1436920_at | NM_001013753 | Pcdh17 | protocadherin 17 | 2,73 | 6,04E-03 |
| 1425871_a_at | --- | Igk-V28 /// Igkv12-46 | immunoglobulin kappa chain variable 28 (V28) /// immunoglobulin kappa chain variable 12-46 | 2,73 | 1,15E-05 |
| 1435460_at | NM_008926 | Prkg2 | protein kinase, cGMP-dependent, type II | 2,74 | 9,33E-04 |
| 1455996_x_at | NM_009475 | Prap1 | proline-rich acidic protein 1 | 2,74 | 8,65E-06 |
| 1447394_at | NM_001039222 | Clca6 /// Gm6289 | chloride channel calcium activated 6 /// predicted gene 6289 | 2,75 | 2,89E-04 |
| 1456377_x_at | NM_172397 | Limd2 | LIM domain containing 2 | 2,77 | 6,81E-06 |
| 1422352_at | NM_008570 | Mcpt1 | mast cell protease 1 | 2,83 | 1,49E-06 |
| 1452905_at | NR_003633 | Meg3 | maternally expressed 3 | 2,84 | 8,35E-05 |
| 1417803_at | NM_001164210 | 1110032A04Rik | RIKEN cDNA 1110032A04 gene | 2,85 | 2,29E-05 |
| 1448741_at | NM_009205 | Slc3a1 | solute carrier family 3, member 1 | 2,85 | 2,62E-05 |
| 1422860_at | NM_024435 | Nts | neurotensin | 2,92 | 7,79E-04 |
| 1452967_at | NM_028797 | Gtsf1 | gametocyte specific factor 1 | 2,94 | 3,15E-03 |
| 1425237_at | NM_130856 | Krtap16-8 | keratin associated protein 16-8 | 3,01 | 1,52E-03 |
| 1427747_a_at | NM_008491 | Lcn2 | lipocalin 2 | 3,01 | 6,49E-07 |
| 1456815_at | NM_008238 | Foxn1 | forkhead box N1 | 3,03 | 1,85E-04 |
| 1460423_x_at | --- | Gm5571 /// Igk-V1 | predicted gene 5571 /// immunoglobulin kappa chain variable 1 (V1) | 3,08 | 2,11E-04 |
| 1429994_s_at | NM_028191 | Cyp2c65 | cytochrome P450, family 2, subfamily c, polypeptide 65 | 3,08 | 9,86E-07 |
| 1426439_at | NM_012008 | Ddx3y | DEAD (Asp-Glu-Ala-Asp) box polypeptide 3, Y-linked | 3,08 | 2,47E-03 |
| 1417292_at | NM_008330 | Ifi47 | interferon gamma inducible protein 47 | 3,10 | 7,20E-05 |
| 1449939_s_at | NM_001190703 | Dlk1 | delta-like 1 homolog (Drosophila) | 3,13 | 3,87E-06 |
| 1437534_at | --- | --- | --- | 3,14 | 1,63E-04 |
| 1419042_at | NM_001146275 | Iigp1 | interferon inducible GTPase 1 | 3,19 | 1,18E-06 |
| 1435162_at | NM_008926 | Prkg2 | protein kinase, cGMP-dependent, type II | 3,22 | 1,73E-05 |
| 1454623_at | NM_001024698 | Cpa2 | carboxypeptidase A2, pancreatic | 3,24 | 1,64E-05 |
| 1456212_x_at | NM_007707 | Socs3 | suppressor of cytokine signaling 3 | 3,26 | 7,00E-05 |
| 1415884_at | NM_026419 | Cela3b | chymotrypsin-like elastase family, member 3B | 3,26 | 1,72E-04 |
| 1422892_s_at | NM_010381 | H2-Ea-ps /// LOC100504404 | histocompatibility 2, class II antigen E alpha, pseudogene /// h-2 class II histocompatibility antigen, E-K alpha chain-like | 3,27 | 2,16E-05 |
| 1424451_at | NM_146230 | Acaa1b | acetyl-Coenzyme A acyltransferase 1B | 3,31 | 6,53E-04 |
| 1419043_a_at | NM_001146275 | Iigp1 | interferon inducible GTPase 1 | 3,34 | 3,50E-04 |
| 1417556_at | NM_017399 | Fabp1 | fatty acid binding protein 1, liver | 3,41 | 5,67E-03 |
| 1449986_at | NM_054100 | 2310034C09Rik | RIKEN cDNA 2310034C09 gene | 3,45 | 1,63E-04 |
| 1455899_x_at | NM_007707 | Socs3 | suppressor of cytokine signaling 3 | 3,49 | 1,89E-05 |
| 1449032_at | NM_019991 | Prl2a1 | prolactin family 2, subfamily a, member 1 | 3,50 | 3,92E-05 |
| 1424903_at | NM_011419 | Kdm5d | lysine (K)-specific demethylase 5D | 3,51 | 4,28E-04 |
| 1423933_a_at | NM_029639 | 1600029D21Rik | RIKEN cDNA 1600029D21 gene | 3,61 | 2,39E-04 |
| 1416139_at | NM_009043 | Reg2 | regenerating islet-derived 2 | 3,80 | 3,64E-05 |
| 1418805_at | NM_011328 | Sct | secretin | 3,81 | 9,07E-07 |
| 1437060_at | NM_001030294 | Olfm4 | olfactomedin 4 | 3,84 | 1,63E-04 |
| 1418652_at | NM_008599 | Cxcl9 | chemokine (C-X-C motif) ligand 9 | 3,88 | 2,62E-04 |
| 1415777_at | NM_018874 | Pnliprp1 | pancreatic lipase related protein 1 | 3,98 | 1,35E-04 |
| 1450624_at | NM_016668 | Bhmt | betaine-homocysteine methyltransferase | 4,02 | 2,38E-03 |
| 1436879_x_at | NM_007423 | Afp | alpha fetoprotein | 4,03 | 1,36E-05 |
| 1419400_at | NM_001163457 | Mttp | microsomal triglyceride transfer protein | 4,03 | 3,28E-07 |
| 1448194_a_at | NR_001592 | H19 | H19 fetal liver mRNA | 4,13 | 2,55E-04 |
| 1437015_x_at | NM_011107 | Pla2g1b | phospholipase A2, group IB, pancreas | 4,14 | 8,62E-04 |
| 1419394_s_at | NM_013650 | S100a8 | S100 calcium binding protein A8 (calgranulin A) | 4,19 | 1,08E-05 |
| 1418069_at | NM_009695 | Apoc2 | apolipoprotein C-II | 4,22 | 1,86E-04 |
| 1416523_at | NM_011271 | Rnase1 | ribonuclease, RNase A family, 1 (pancreatic) | 4,24 | 5,52E-04 |
| 1419728_at | NM_009141 | Cxcl5 | chemokine (C-X-C motif) ligand 5 | 4,25 | 1,41E-04 |
| 1417210_at | NM_012011 | Eif2s3y | eukaryotic translation initiation factor 2, subunit 3, structural gene Y-linked | 4,25 | 6,45E-05 |
| 1454254_s_at | NM_029639 | 1600029D21Rik | RIKEN cDNA 1600029D21 gene | 4,26 | 2,39E-05 |
| 1449009_at | NM_001145164 | Tgtp1 /// Tgtp2 | T-cell specific GTPase 1 /// T-cell specific GTPase 2 | 4,31 | 1,46E-06 |
| 1419622_at | NM_009467 | Ugt2b5 | UDP glucuronosyltransferase 2 family, polypeptide B5 | 4,41 | 2,59E-05 |
| 1453070_at | NM_001013753 | Pcdh17 | protocadherin 17 | 4,42 | 7,24E-06 |
| 1417078_at | NM_025622 | Lgals2 | lectin, galactose-binding, soluble 2 | 4,43 | 2,36E-07 |
| 1420437_at | NM_008324 | Ido1 | indoleamine 2,3-dioxygenase 1 | 4,43 | 3,17E-04 |
| 1417079_s_at | NM_025622 | Lgals2 | lectin, galactose-binding, soluble 2 | 4,48 | 4,46E-05 |
| 1450682_at | NM_008375 | Fabp6 | fatty acid binding protein 6, ileal (gastrotropin) | 4,49 | 1,31E-04 |
| 1441836_x_at | NM_028797 | Gtsf1 | Gametocyte specific factor 1 | 4,54 | 6,90E-04 |
| 1417290_at | NM_029796 | Lrg1 | leucine-rich alpha-2-glycoprotein 1 | 4,60 | 1,45E-05 |
| 1416777_at | NM_001162523 | Ceacam12 | carcinoembryonic antigen-related cell adhesion molecule 12 | 4,64 | 7,04E-06 |
| 1422240_s_at | NM_011474 | Sprr2h | small proline-rich protein 2H | 4,66 | 3,97E-04 |
| 1451551_at | NM_008474 | Krt84 | keratin 84 | 4,73 | 8,13E-06 |
| 1435611_x_at | NM_026419 | Cela3b | chymotrypsin-like elastase family, member 3B | 4,76 | 1,20E-05 |
| 1451934_at | NM_010380 | H2-D1 | histocompatibility 2, D region locus 1 | 4,77 | 3,79E-04 |
| 1417257_at | NM_009885 | Cel | carboxyl ester lipase | 4,85 | 1,90E-06 |
| 1434747_at | NM_001033875 | Ctrc | chymotrypsin C (caldecrin) | 4,86 | 2,14E-06 |
| 1417682_a_at | NM_009430 | Prss2 | protease, serine, 2 | 4,87 | 2,38E-04 |
| 1448220_at | NM_025583 | Ctrb1 | chymotrypsinogen B1 | 4,89 | 2,96E-04 |
| 1426438_at | NM_012008 | Ddx3y | DEAD (Asp-Glu-Ala-Asp) box polypeptide 3, Y-linked | 4,94 | 2,50E-06 |
| 1433573_x_at | NM_009430 | Prss2 | protease, serine, 2 | 4,95 | 2,67E-04 |
| 1448764_a_at | NM_017399 | Fabp1 | fatty acid binding protein 1, liver | 4,97 | 1,08E-03 |
| 1433459_x_at | NM_009430 | Prss2 | protease, serine, 2 | 5,02 | 1,22E-05 |
| 1437867_at | --- | --- | --- | 5,05 | 6,25E-06 |
| 1439796_at | NM_001033199 | AI747448 | expressed sequence AI747448 | 5,08 | 1,91E-07 |
| 1431077_at | NM_001081082 | Alpi | alkaline phosphatase, intestinal | 5,08 | 4,43E-06 |
| 1451030_at | NM_029901 | Akr1c21 | aldo-keto reductase family 1, member C21 | 5,12 | 2,89E-04 |
| 1431763_a_at | NM_023182 | Ctrl | chymotrypsin-like | 5,21 | 1,46E-03 |
| 1435507_x_at | NM_009430 | Prss2 | protease, serine, 2 | 5,27 | 3,30E-04 |
| 1416649_at | NM_007443 | Ambp | alpha 1 microglobulin/bikunin | 5,38 | 1,69E-08 |
| 1415883_a_at | NM_026419 | Cela3b | chymotrypsin-like elastase family, member 3B | 5,43 | 8,91E-08 |
| 1416645_a_at | NM_007423 | Afp | alpha fetoprotein | 5,46 | 1,33E-05 |
| 1428062_at | NM_025350 | Cpa1 | carboxypeptidase A1 | 5,64 | 1,05E-04 |
| 1422435_at | NM_023333 | 2210010C04Rik | RIKEN cDNA 2210010C04 gene | 5,65 | 2,01E-03 |
| 1415905_at | NM_009042 | Reg1 | regenerating islet-derived 1 | 5,69 | 6,01E-07 |
| 1421868_a_at | NM_026925 | Pnlip | pancreatic lipase | 5,73 | 5,16E-04 |
| 1448290_at | NM_011036 | Reg3b | regenerating islet-derived 3 beta | 7,07 | 2,26E-04 |
| 1416297_s_at | NM_011036 | Reg3b | regenerating islet-derived 3 beta | 7,07 | 1,77E-05 |
| 1428102_at | NM_029706 | Cpb1 | carboxypeptidase B1 (tissue) | 7,24 | 6,42E-04 |
| 1437326_x_at | NM_026419 | Cela3b | chymotrypsin-like elastase family, member 3B | 7,33 | 9,41E-05 |
| 1448281_a_at | NM_007919 | Cela2a | chymotrypsin-like elastase family, member 2A | 7,40 | 4,15E-03 |
| 1433431_at | NM_026925 | Pnlip | pancreatic lipase | 7,42 | 1,38E-04 |
| 1448872_at | NM_011260 | Reg3g | regenerating islet-derived 3 gamma | 7,61 | 1,18E-04 |
| 1422434_a_at | NM_023333 | 2210010C04Rik | RIKEN cDNA 2210010C04 gene | 8,19 | 9,98E-05 |
| 1415954_at | NM_001003405 | Try4 /// Try5 | trypsin 4 /// trypsin 5 | 8,62 | 6,37E-05 |
| 1435012_x_at | NM_001126318 | Cela3b /// Gm13011 | chymotrypsin-like elastase family, member 3B /// predicted gene 13011 | 9,21 | 1,65E-04 |
| 1416055_at | NM_001042711 | Amy2a4 /// Amy2a5 | amylase 2a4 /// amylase 2a5 | 9,60 | 8,16E-05 |
