# Supplementary material for: HDAC1 and HDAC2 Restrain the Intestinal Inflammatory Response by Regulating Intestinal Epithelial Cell Differentiation
Source: PLoS One. 2013 Sep 5;8(9):e73785. doi: 10.1371/journal.pone.0073785 (PMC3764035; doi:10.1371/journal.pone.0073785)
Supplement: Table S3 — List of immune and/or defense response genes with significant 2-fold increased or decreased expression levels in HDAC1/2-depleted murine colons as determined by microarray analysis, and classified according to GO database. (DOCX) [file pone.0073785.s007.docx]

**Table S3**

List of immune and/or defense response genes with significant 2-fold increased or decreased expression levels in HDAC1/2-depleted murine colons as determined by microarray analysis, and classified according to GO database.

| immune response (GO:0006955); defense response (GO:0006952) | | | |
| --- | --- | --- | --- |
|  |  |  |  |
| Gene Symbol | Gene Title | Fold change (log2) | P-value |
| Adra2a | adrenergic receptor, alpha 2a | -3,11 | 8,27E-05 |
| Ace2 | angiotensin I converting enzyme (peptidyl-dipeptidase A) 2 | -3,11 | 8,27E-05 |
| Ang4 | angiogenin, ribonuclease A family, member 4 | -2,90 | 8,17E-05 |
| Ang4 | angiogenin, ribonuclease A family, member 4 | -2,86 | 7,67E-08 |
| Ppbp | pro-platelet basic protein | -2,69 | 1,97E-03 |
| Arg2 | arginase type II | -2,56 | 1,27E-04 |
| H2-Q2 | histocompatibility 2, Q region locus 2 | -2,49 | 3,04E-03 |
| Edn1 | endothelin 1 | -2,29 | 2,90E-04 |
| Arg2 | arginase type II | -2,09 | 7,57E-04 |
| Ace2 | angiotensin I converting enzyme (peptidyl-dipeptidase A) 2 | -2,06 | 1,61E-03 |
| Crisp3 | cysteine-rich secretory protein 3 | -2,05 | 2,36E-03 |
| Edn2 | endothelin 2 | -1,90 | 1,42E-05 |
| Prdm16 | PR domain containing 16 | -1,81 | 1,38E-03 |
| Nt5e | 5' nucleotidase, ecto | -1,72 | 2,20E-04 |
| Prdm16 | PR domain containing 16 | -1,62 | 3,24E-03 |
| Sike1 | suppressor of IKBKE 1 | -1,60 | 3,76E-05 |
| Krt1 | keratin 1 | -1,53 | 4,13E-02 |
| Sectm1a | secreted and transmembrane 1A | -1,49 | 3,82E-05 |
| Nr1d1 | nuclear receptor subfamily 1, group D, member 1 | -1,38 | 4,19E-03 |
| Cma1 | chymase 1, mast cell | -1,33 | 4,62E-04 |
| C4bp | complement component 4 binding protein | -1,31 | 1,24E-05 |
| Ccl6 | chemokine (C-C motif) ligand 6 | -1,31 | 6,18E-05 |
| Il15 | interleukin 15 | -1,28 | 8,40E-06 |
| Saa1 | serum amyloid A 1 | -1,25 | 1,10E-06 |
| Tgfb3 | transforming growth factor, beta 3 | -1,23 | 2,25E-03 |
| Chst4 | carbohydrate (chondroitin 6/keratan) sulfotransferase 4 | -1,22 | 2,65E-06 |
| Sike1 | suppressor of IKBKE 1 | -1,19 | 7,22E-05 |
| Prdm16 | PR domain containing 16 | -1,19 | 2,73E-03 |
| Cd55 | CD55 antigen | -1,18 | 7,94E-04 |
| Enpp1 | ectonucleotide pyrophosphatase/phosphodiesterase 1 | -1,14 | 2,86E-05 |
| Plekha1 | pleckstrin homology domain containing, family A (phosphoinositide binding specific) member 1 | -1,12 | 7,28E-05 |
| Igfbp2 | insulin-like growth factor binding protein 2 | -1,08 | 1,74E-04 |
| Dpp4 | dipeptidylpeptidase 4 | -1,08 | 2,44E-03 |
| Fcgrt | Fc receptor, IgG, alpha chain transporter | -1,07 | 1,70E-04 |
| Txnip | thioredoxin interacting protein | -1,05 | 2,11E-04 |
| Btn1a1 | butyrophilin, subfamily 1, member A1 | -1,03 | 3,96E-03 |
| Pla2g4a | phospholipase A2, group IVA (cytosolic, calcium-dependent) | -1,01 | 2,71E-04 |
| Rsad2 | radical S-adenosyl methionine domain containing 2 | 1,00 | 1,91E-04 |
| Zbp1 | Z-DNA binding protein 1 | 1,00 | 4,02E-05 |
| H2-D1 | histocompatibility 2, D region locus 1 | 1,01 | 1,18E-04 |
| Gbp6 | guanylate binding protein 6 | 1,01 | 2,93E-03 |
| Arhgdib | Rho, GDP dissociation inhibitor (GDI) beta | 1,01 | 3,59E-03 |
| Cd28 | CD28 antigen | 1,01 | 1,62E-03 |
| Gp49a /// Lilrb4 | glycoprotein 49 A /// leukocyte immunoglobulin-like receptor, subfamily B, member 4 | 1,02 | 8,81E-03 |
| Il17b | interleukin 17B | 1,02 | 2,24E-04 |
| Id2 | inhibitor of DNA binding 2 | 1,02 | 7,92E-04 |
| Gm9706 /// Isg15 | predicted gene 9706 /// ISG15 ubiquitin-like modifier | 1,03 | 6,06E-05 |
| Zfpm1 | zinc finger protein, multitype 1 | 1,03 | 2,16E-05 |
| Cyba | cytochrome b-245, alpha polypeptide | 1,03 | 2,19E-05 |
| C1s | complement component 1, s subcomponent | 1,03 | 2,60E-05 |
| Stat1 | signal transducer and activator of transcription 1 | 1,04 | 7,61E-06 |
| Igh-2 /// Igh-VJ558 /// LOC544903 | immunoglobulin heavy chain 2 (serum IgA) /// immunoglobulin heavy chain (J558 family) /// similar to immunoglobulin mu-chain | 1,04 | 8,97E-06 |
| Itga4 | integrin alpha 4 | 1,04 | 3,35E-03 |
| Igfbp4 | insulin-like growth factor binding protein 4 | 1,05 | 1,57E-05 |
| Pla2g2d | phospholipase A2, group IID | 1,05 | 6,89E-05 |
| Igh-VJ558 | Immunoglobulin heavy chain (J558 family) | 1,05 | 5,43E-04 |
| Lcp2 | lymphocyte cytosolic protein 2 | 1,05 | 1,47E-03 |
| Il2rg | interleukin 2 receptor, gamma chain | 1,06 | 3,98E-04 |
| Tmem176a | transmembrane protein 176A | 1,06 | 1,70E-03 |
| Adra2a | adrenergic receptor, alpha 2a | 1,07 | 9,37E-03 |
| H2-DMa | histocompatibility 2, class II, locus DMa | 1,07 | 4,35E-03 |
| Ncf4 | neutrophil cytosolic factor 4 | 1,07 | 1,51E-03 |
| Il16 | interleukin 16 | 1,07 | 1,39E-04 |
| Cybb | cytochrome b-245, beta polypeptide | 1,07 | 1,44E-02 |
| C1qc | complement component 1, q subcomponent, C chain | 1,07 | 2,60E-05 |
| Mndal | myeloid nuclear differentiation antigen like | 1,07 | 3,24E-03 |
| Rnase6 | ribonuclease, RNase A family, 6 | 1,08 | 3,15E-03 |
| Gbp6 | guanylate binding protein 6 | 1,08 | 3,98E-04 |
| Tnfsf10 | tumor necrosis factor (ligand) superfamily, member 10 | 1,08 | 4,16E-03 |
| C1qb | complement component 1, q subcomponent, beta polypeptide | 1,09 | 6,44E-03 |
| Faim3 | Fas apoptotic inhibitory molecule 3 | 1,09 | 1,99E-04 |
| Fcgr3 | Fc receptor, IgG, low affinity III | 1,10 | 2,76E-03 |
| Ltb | lymphotoxin B | 1,10 | 2,04E-04 |
| C1qb | complement component 1, q subcomponent, beta polypeptide | 1,10 | 9,10E-04 |
| H2-D1 | histocompatibility 2, D region locus 1 | 1,11 | 8,29E-04 |
| Pla2g2d | phospholipase A2, group IID | 1,11 | 4,34E-04 |
| Prdm1 | PR domain containing 1, with ZNF domain | 1,12 | 9,31E-04 |
| March1 | membrane-associated ring finger (C3HC4) 1 | 1,12 | 1,91E-03 |
| Csf1r | colony stimulating factor 1 receptor | 1,12 | 6,09E-06 |
| Rbp1 | retinol binding protein 1, cellular | 1,12 | 6,13E-04 |
| Lcp1 | lymphocyte cytosolic protein 1 | 1,12 | 2,18E-05 |
| Stat1 | signal transducer and activator of transcription 1 | 1,12 | 3,08E-05 |
| Ccl5 | chemokine (C-C motif) ligand 5 | 1,13 | 2,08E-03 |
| Cst7 | cystatin F (leukocystatin) | 1,13 | 1,33E-03 |
| Apoe | apolipoprotein E | 1,13 | 4,53E-04 |
| Serpina1b | serine (or cysteine) preptidase inhibitor, clade A, member 1B | 1,13 | 3,41E-03 |
| Cfd | complement factor D (adipsin) | 1,14 | 8,87E-06 |
| Gm10334 /// Gm5771 /// Prss1 /// Prss3 | predicted gene 10334 /// predicted gene 5771 /// protease, serine, 1 (trypsin 1) /// protease, serine, 3 | 1,15 | 4,61E-03 |
| Tnfsf10 | tumor necrosis factor (ligand) superfamily, member 10 | 1,15 | 5,95E-05 |
| Cd38 | CD38 antigen | 1,15 | 4,29E-04 |
| Ciita | class II transactivator | 1,15 | 1,70E-03 |
| H2-D1 | histocompatibility 2, D region locus 1 | 1,16 | 1,35E-05 |
| Klhl6 | kelch-like 6 (Drosophila) | 1,16 | 7,31E-05 |
| Ccr1 | chemokine (C-C motif) receptor 1 | 1,16 | 1,91E-04 |
| Nlrp10 | NLR family, pyrin domain containing 10 | 1,17 | 1,45E-03 |
| Tnfrsf13b | tumor necrosis factor receptor superfamily, member 13b | 1,18 | 1,75E-04 |
| Cd48 | CD48 antigen | 1,18 | 1,02E-03 |
| Cotl1 | coactosin-like 1 (Dictyostelium) | 1,19 | 8,78E-05 |
| C1qb | complement component 1, q subcomponent, beta polypeptide | 1,19 | 1,66E-04 |
| Ly86 | lymphocyte antigen 86 | 1,19 | 6,87E-05 |
| Tff2 | trefoil factor 2 (spasmolytic protein 1) | 1,19 | 2,90E-03 |
| Nfkbiz | nuclear factor of kappa light polypeptide gene enhancer in B-cells inhibitor, zeta | 1,19 | 1,35E-04 |
| Hsp90aa1 | heat shock protein 90, alpha (cytosolic), class A member 1 | 1,20 | 2,47E-04 |
| Muc4 | mucin 4 | 1,20 | 2,03E-04 |
| Ikzf1 | IKAROS family zinc finger 1 | 1,21 | 2,71E-03 |
| Was | Wiskott-Aldrich syndrome homolog (human) | 1,22 | 1,99E-03 |
| Hsp90b1 | heat shock protein 90, beta (Grp94), member 1 | 1,22 | 2,10E-04 |
| Serpina1a /// Serpina1b | serine (or cysteine) peptidase inhibitor, clade A, member 1A /// serine (or cysteine) preptidase inhibitor, clade A, member 1B | 1,23 | 1,57E-03 |
| Cxcr4 | chemokine (C-X-C motif) receptor 4 | 1,23 | 7,11E-03 |
| H2-Q6 /// LOC68395 | histocompatibility 2, Q region locus 6 /// histocompatibility 2, Q region locus 6-like | 1,23 | 2,79E-04 |
| Igj | immunoglobulin joining chain | 1,24 | 9,72E-05 |
| Cotl1 | coactosin-like 1 (Dictyostelium) | 1,24 | 5,37E-06 |
| Hcls1 | hematopoietic cell specific Lyn substrate 1 | 1,24 | 9,49E-04 |
| Apol7c /// Gm8221 | apolipoprotein L 7c /// apolipoprotein L, 3-like | 1,24 | 9,12E-04 |
| Clec4n | C-type lectin domain family 4, member n | 1,24 | 1,14E-03 |
| IghmAC38.205.12 /// LOC100505009 /// LOC634206 | Ig mu chain V region AC38 205.12 /// similar to immunoglobulin mu-chain-like /// ig heavy chain V region 108A-like | 1,24 | 3,64E-03 |
| Gzmb | granzyme B | 1,24 | 2,10E-02 |
| Lpxn | leupaxin | 1,25 | 5,13E-04 |
| Tyrobp | TYRO protein tyrosine kinase binding protein | 1,25 | 1,75E-05 |
| C3ar1 | complement component 3a receptor 1 | 1,25 | 9,81E-04 |
| Ggt1 | gamma-glutamyltransferase 1 | 1,26 | 1,17E-03 |
| Ripk3 | receptor-interacting serine-threonine kinase 3 | 1,28 | 3,64E-04 |
| Fcer1g | Fc receptor, IgE, high affinity I, gamma polypeptide | 1,28 | 1,53E-05 |
| Gm10693 /// Gm14548 /// Lilra6 /// Lilrb3 /// Pira1 /// Pira11 /// Pira2 /// Pira4 /// Pira6 /// Pira7 | predicted pseudogene 10693 /// predicted gene 14548 /// leukocyte immunoglobulin-like receptor, subfamily A (with TM domain), member 6 /// leukocyte immunoglobulin-like receptor, subfamily B (with TM and ITIM domains), member 3 /// paired-Ig-like receptor A1 /// paired-Ig-like receptor A11 /// paired-Ig-like receptor A2 /// paired-Ig-like receptor A4 /// paired-Ig-like receptor A6 /// paired-Ig-like receptor A7 | 1,29 | 4,65E-03 |
| Hpx | hemopexin | 1,29 | 7,15E-03 |
| H2-Q10 | histocompatibility 2, Q region locus 10 | 1,29 | 2,98E-04 |
| Clec7a | C-type lectin domain family 7, member a | 1,30 | 3,53E-04 |
| Ifi205 /// Mnda | interferon activated gene 205 /// myeloid cell nuclear differentiation antigen | 1,31 | 1,59E-03 |
| Usp18 | ubiquitin specific peptidase 18 | 1,32 | 1,17E-03 |
| H2-Ob | histocompatibility 2, O region beta locus | 1,32 | 1,14E-02 |
| Icos | inducible T-cell co-stimulator | 1,32 | 4,04E-05 |
| Cxcl2 | chemokine (C-X-C motif) ligand 2 | 1,32 | 4,84E-04 |
| Dusp3 | dual specificity phosphatase 3 (vaccinia virus phosphatase VH1-related) | 1,32 | 2,58E-04 |
| Cd86 | CD86 antigen | 1,32 | 1,30E-03 |
| C3 | complement component 3 | 1,32 | 5,45E-05 |
| Lck | lymphocyte protein tyrosine kinase | 1,33 | 1,01E-04 |
| C1qa | complement component 1, q subcomponent, alpha polypeptide | 1,33 | 1,29E-05 |
| Ptprc | protein tyrosine phosphatase, receptor type, C | 1,34 | 3,97E-05 |
| Cotl1 | coactosin-like 1 (Dictyostelium) | 1,35 | 1,24E-03 |
| C4b | complement component 4B (Childo blood group) | 1,35 | 1,79E-06 |
| Stat1 | signal transducer and activator of transcription 1 | 1,36 | 7,87E-03 |
| Zbp1 | Z-DNA binding protein 1 | 1,36 | 1,44E-03 |
| Gch1 | GTP cyclohydrolase 1 | 1,36 | 9,94E-06 |
| Itgb2 | integrin beta 2 | 1,37 | 3,86E-04 |
| Cotl1 | coactosin-like 1 (Dictyostelium) | 1,37 | 5,10E-06 |
| Aif1 | allograft inflammatory factor 1 | 1,38 | 5,72E-03 |
| Pira2 | paired-Ig-like receptor A2 | 1,39 | 4,88E-04 |
| Tfrc | transferrin receptor | 1,39 | 2,26E-06 |
| H2-Ab1 | histocompatibility 2, class II antigen A, beta 1 | 1,40 | 3,76E-07 |
| Lcp1 | lymphocyte cytosolic protein 1 | 1,40 | 7,47E-06 |
| Cybb | cytochrome b-245, beta polypeptide | 1,41 | 8,79E-05 |
| Il18bp | interleukin 18 binding protein | 1,42 | 5,92E-05 |
| Ada | adenosine deaminase | 1,43 | 8,10E-06 |
| Ifitm1 | interferon induced transmembrane protein 1 | 1,44 | 1,02E-04 |
| Vcam1 | vascular cell adhesion molecule 1 | 1,44 | 1,47E-04 |
| Klrd1 | killer cell lectin-like receptor, subfamily D, member 1 | 1,44 | 1,83E-03 |
| Pla2g1b | phospholipase A2, group IB, pancreas | 1,44 | 1,26E-04 |
| Fcgr2b | Fc receptor, IgG, low affinity IIb | 1,46 | 9,93E-04 |
| Prdx2 | peroxiredoxin 2 | 1,46 | 1,52E-04 |
| Fcgr4 | Fc receptor, IgG, low affinity IV | 1,47 | 1,79E-05 |
| Coro1a | coronin, actin binding protein 1A | 1,47 | 2,10E-03 |
| H2-DMb2 | histocompatibility 2, class II, locus Mb2 | 1,47 | 5,45E-03 |
| Ptpn22 | protein tyrosine phosphatase, non-receptor type 22 (lymphoid) | 1,47 | 1,80E-04 |
| Il2rg | interleukin 2 receptor, gamma chain | 1,48 | 3,62E-04 |
| Ms4a1 | membrane-spanning 4-domains, subfamily A, member 1 | 1,48 | 3,80E-05 |
| Ctsc | Cathepsin C | 1,50 | 2,27E-03 |
| Ms4a1 | membrane-spanning 4-domains, subfamily A, member 1 | 1,50 | 1,69E-03 |
| Coro1a | coronin, actin binding protein 1A | 1,51 | 3,28E-04 |
| Ereg | epiregulin | 1,51 | 1,43E-04 |
| Clec4n | C-type lectin domain family 4, member n | 1,51 | 1,58E-04 |
| Psmb8 | proteasome (prosome, macropain) subunit, beta type 8 (large multifunctional peptidase 7) | 1,51 | 1,08E-02 |
| Ikzf3 | IKAROS family zinc finger 3 | 1,51 | 1,10E-03 |
| Nfkbiz | nuclear factor of kappa light polypeptide gene enhancer in B-cells inhibitor, zeta | 1,52 | 7,36E-05 |
| Cd28 | CD28 antigen | 1,53 | 1,21E-04 |
| Hsph1 | heat shock 105kDa/110kDa protein 1 | 1,54 | 2,55E-05 |
| H2-Q7 | histocompatibility 2, Q region locus 7 | 1,55 | 9,37E-04 |
| Ifi204 /// Ifi205 /// Mnda /// Mndal | interferon activated gene 204 /// interferon activated gene 205 /// myeloid cell nuclear differentiation antigen /// myeloid nuclear differentiation antigen like | 1,55 | 1,43E-04 |
| H2-Q8 | histocompatibility 2, Q region locus 8 | 1,56 | 4,11E-03 |
| Gbp3 | guanylate binding protein 3 | 1,57 | 7,30E-04 |
| Ccr5 | chemokine (C-C motif) receptor 5 | 1,57 | 4,02E-03 |
| Ctsc | cathepsin C | 1,57 | 1,35E-04 |
| Cxcl10 | chemokine (C-X-C motif) ligand 10 | 1,58 | 3,66E-03 |
| Cck | cholecystokinin | 1,59 | 9,13E-05 |
| Cd3g | CD3 antigen, gamma polypeptide | 1,62 | 1,43E-03 |
| Gpr65 | G-protein coupled receptor 65 | 1,63 | 4,35E-03 |
| H2-Aa | histocompatibility 2, class II antigen A, alpha | 1,64 | 2,51E-04 |
| Clcf1 | cardiotrophin-like cytokine factor 1 | 1,64 | 5,01E-04 |
| Stat1 | signal transducer and activator of transcription 1 | 1,64 | 8,34E-07 |
| Cd79b | CD79B antigen | 1,65 | 3,87E-06 |
| Hsph1 | heat shock 105kDa/110kDa protein 1 | 1,66 | 1,30E-05 |
| Icam1 | intercellular adhesion molecule 1 | 1,67 | 2,24E-06 |
| Cotl1 | coactosin-like 1 (Dictyostelium) | 1,67 | 1,15E-05 |
| Trpv1 | transient receptor potential cation channel, subfamily V, member 1 | 1,69 | 1,50E-05 |
| Pou2af1 | POU domain, class 2, associating factor 1 | 1,72 | 4,65E-06 |
| Igh-3 /// Ighg | immunoglobulin heavy chain 3 (serum IgG2b) /// Immunoglobulin heavy chain (gamma polypeptide) | 1,75 | 2,97E-04 |
| H2-Ab1 | histocompatibility 2, class II antigen A, beta 1 | 1,76 | 1,47E-07 |
| Spp1 | secreted phosphoprotein 1 | 1,78 | 4,57E-05 |
| Il1rl1 | interleukin 1 receptor-like 1 | 1,81 | 3,57E-05 |
| Socs3 | suppressor of cytokine signaling 3 | 1,81 | 5,36E-06 |
| Mmp7 | matrix metallopeptidase 7 | 1,82 | 1,40E-04 |
| Cd74 | CD74 antigen (invariant polypeptide of major histocompatibility complex, class II antigen-associated) | 1,83 | 2,30E-05 |
| Tac1 | tachykinin 1 | 1,84 | 1,92E-06 |
| Ccl25 | chemokine (C-C motif) ligand 25 | 1,84 | 9,59E-05 |
| Ighg | Immunoglobulin heavy chain (gamma polypeptide) | 1,85 | 5,41E-06 |
| Cxcl13 | chemokine (C-X-C motif) ligand 13 | 1,85 | 8,41E-05 |
| Gbp10 /// Mpa2l | guanylate-binding protein 10 /// macrophage activation 2 like | 1,86 | 1,23E-05 |
| H2-DMb2 | histocompatibility 2, class II, locus Mb2 | 1,87 | 9,98E-05 |
| Cxcl3 | chemokine (C-X-C motif) ligand 3 | 1,89 | 1,61E-03 |
| Gch1 | GTP cyclohydrolase 1 | 1,90 | 2,03E-07 |
| Cxcl9 | chemokine (C-X-C motif) ligand 9 | 1,90 | 7,02E-03 |
| Itgal | integrin alpha L | 1,92 | 8,47E-04 |
| Mpa2l | macrophage activation 2 like | 1,93 | 1,81E-03 |
| Cxcl13 | chemokine (C-X-C motif) ligand 13 | 1,95 | 4,20E-04 |
| Psmb8 | proteasome (prosome, macropain) subunit, beta type 8 (large multifunctional peptidase 7) | 1,96 | 2,70E-06 |
| Nlrc5 | NLR family, CARD domain containing 5 | 1,98 | 2,14E-05 |
| H2-Ab1 | histocompatibility 2, class II antigen A, beta 1 | 1,98 | 6,75E-07 |
| Psmb9 | proteasome (prosome, macropain) subunit, beta type 9 (large multifunctional peptidase 2) | 1,99 | 4,11E-06 |
| H2-DMb2 | histocompatibility 2, class II, locus Mb2 | 2,00 | 1,22E-05 |
| Igh-VJ558 /// LOC675759 | immunoglobulin heavy chain (J558 family) /// ig heavy chain V-III region J606-like | 2,04 | 1,99E-05 |
| Ifit2 | interferon-induced protein with tetratricopeptide repeats 2 | 2,07 | 6,39E-04 |
| Ccl8 | chemokine (C-C motif) ligand 8 | 2,08 | 2,14E-03 |
| Serpina3n | serine (or cysteine) peptidase inhibitor, clade A, member 3N | 2,08 | 7,77E-05 |
| Gbp2 | guanylate binding protein 2 | 2,10 | 8,60E-05 |
| Gbp8 | guanylate-binding protein 8 | 2,12 | 2,08E-04 |
| H2-Aa | histocompatibility 2, class II antigen A, alpha | 2,13 | 1,43E-04 |
| Gbp2 | guanylate binding protein 2 | 2,13 | 4,73E-07 |
| Igh-6 | Immunoglobulin heavy chain 6 (heavy chain of IgM) | 2,13 | 2,20E-04 |
| S100a9 | S100 calcium binding protein A9 (calgranulin B) | 2,17 | 2,84E-06 |
| Reg3a | regenerating islet-derived 3 alpha | 2,18 | 4,80E-04 |
| H2-DMa | histocompatibility 2, class II, locus DMa | 2,22 | 3,95E-05 |
| H2-Aa | histocompatibility 2, class II antigen A, alpha | 2,24 | 2,41E-04 |
| H2-Q5 | histocompatibility 2, Q region locus 5 | 2,24 | 5,15E-03 |
| Serpina3g | serine (or cysteine) peptidase inhibitor, clade A, member 3G | 2,26 | 1,67E-03 |
| H2-Eb1 | histocompatibility 2, class II antigen E beta | 2,27 | 4,19E-05 |
| Wfdc12 | WAP four-disulfide core domain 12 | 2,28 | 5,87E-05 |
| Saa3 | serum amyloid A 3 | 2,29 | 1,11E-03 |
| Pla2g2a | phospholipase A2, group IIA (platelets, synovial fluid) | 2,29 | 3,50E-03 |
| Gp2 | glycoprotein 2 (zymogen granule membrane) | 2,32 | 1,37E-05 |
| H2-DMb1 /// H2-DMb2 | histocompatibility 2, class II, locus Mb1 /// histocompatibility 2, class II, locus Mb2 | 2,32 | 4,77E-05 |
| H2-Ea-ps /// LOC100504404 | histocompatibility 2, class II antigen E alpha, pseudogene /// h-2 class II histocompatibility antigen, E-K alpha chain-like | 2,33 | 1,15E-05 |
| Cxcl1 | chemokine (C-X-C motif) ligand 1 | 2,39 | 4,74E-06 |
| H2-K1 | Histocompatibility 2, K1, K region | 2,41 | 1,33E-04 |
| Ubd | ubiquitin D | 2,41 | 1,31E-04 |
| Muc4 | mucin 4 | 2,47 | 3,30E-04 |
| H2-DMb1 /// H2-DMb2 | histocompatibility 2, class II, locus Mb1 /// histocompatibility 2, class II, locus Mb2 | 2,52 | 1,81E-04 |
| H2-Aa | histocompatibility 2, class II antigen A, alpha | 2,54 | 2,56E-03 |
| Gzma | granzyme A | 2,55 | 2,12E-03 |
| Lcn2 | lipocalin 2 | 3,01 | 6,49E-07 |
| Foxn1 | forkhead box N1 | 3,03 | 1,85E-04 |
| Ifi47 | interferon gamma inducible protein 47 | 3,10 | 7,20E-05 |
| Socs3 | suppressor of cytokine signaling 3 | 3,26 | 7,00E-05 |
| H2-Ea-ps /// LOC100504404 | histocompatibility 2, class II antigen E alpha, pseudogene /// h-2 class II histocompatibility antigen, E-K alpha chain-like | 3,27 | 2,16E-05 |
| Socs3 | suppressor of cytokine signaling 3 | 3,49 | 1,89E-05 |
| Cxcl9 | chemokine (C-X-C motif) ligand 9 | 3,88 | 2,62E-04 |
| Pla2g1b | phospholipase A2, group IB, pancreas | 4,14 | 8,62E-04 |
| S100a8 | S100 calcium binding protein A8 (calgranulin A) | 4,19 | 1,08E-05 |
| Cxcl5 | chemokine (C-X-C motif) ligand 5 | 4,25 | 1,41E-04 |
| Tgtp1 /// Tgtp2 | T-cell specific GTPase 1 /// T-cell specific GTPase 2 | 4,31 | 1,46E-06 |
| Ido1 | indoleamine 2,3-dioxygenase 1 | 4,43 | 3,17E-04 |
| H2-D1 | histocompatibility 2, D region locus 1 | 4,77 | 3,79E-04 |
| Prss2 | protease, serine, 2 | 5,02 | 1,22E-05 |
| Prss2 | protease, serine, 2 | 5,27 | 3,30E-04 |
| Ambp | alpha 1 microglobulin/bikunin | 5,38 | 1,69E-08 |
| Reg3b | regenerating islet-derived 3 beta | 7,07 | 2,26E-04 |
| Reg3b | regenerating islet-derived 3 beta | 7,07 | 1,77E-05 |
| Reg3g | regenerating islet-derived 3 gamma | 7,61 | 1,18E-04 |
