# Supplementary material for: HDAC1 and HDAC2 Restrain the Intestinal Inflammatory Response by Regulating Intestinal Epithelial Cell Differentiation
Source: PLoS One. 2013 Sep 5;8(9):e73785. doi: 10.1371/journal.pone.0073785 (PMC3764035; doi:10.1371/journal.pone.0073785)
Supplement: Table S4 — List of digestion and/or proteolysis genes with significant 2-fold increased or decreased expression levels in HDAC1/2-depleted murine colons as determined by microarray analysis, and classified according to GO database. (DOCX) [file pone.0073785.s008.docx]

**Table S4**

List of digestion and/or proteolysis genes with significant 2-fold increased or decreased expression levels in HDAC1/2-depleted murine colons as determined by microarray analysis, and classified according to GO database.

| proteolysis (GO:0006508); digestion (GO:0007586) | | | |
| --- | --- | --- | --- |
|  |  |  |  |
| Gene Symbol | Gene Title | Fold change (log2) | P-value |
| Adra2a | adrenergic receptor, alpha 2a | -3,11 | 8,27E-05 |
| Ace2 | angiotensin I converting enzyme (peptidyl-dipeptidase A) 2 | -3,11 | 8,27E-05 |
| Slc15a1 | solute carrier family 15 (oligopeptide transporter), member 1 | -2,56 | 7,67E-04 |
| Tmprss11a | transmembrane protease, serine 11a | -2,48 | 8,99E-04 |
| Pm20d1 | peptidase M20 domain containing 1 | -2,09 | 8,76E-05 |
| Tmprss13 | transmembrane protease, serine 13 | -2,09 | 6,59E-05 |
| Ace2 | angiotensin I converting enzyme (peptidyl-dipeptidase A) 2 | -2,06 | 1,61E-03 |
| Fbxo32 | F-box protein 32 | -1,74 | 6,60E-05 |
| Socs2 | suppressor of cytokine signaling 2 | -1,65 | 1,66E-05 |
| Ppargc1a | peroxisome proliferative activated receptor, gamma, coactivator 1 alpha | -1,60 | 1,40E-04 |
| Pm20d1 | peptidase M20 domain containing 1 | -1,55 | 6,64E-04 |
| Ppargc1a | peroxisome proliferative activated receptor, gamma, coactivator 1 alpha | -1,54 | 2,49E-06 |
| Ppargc1a | peroxisome proliferative activated receptor, gamma, coactivator 1 alpha | -1,51 | 3,55E-05 |
| Pm20d2 | peptidase M20 domain containing 2 | -1,44 | 1,77E-03 |
| Klk1b22 /// Klk1b9 | kallikrein 1-related peptidase b22 /// kallikrein 1-related peptidase b9 | -1,42 | 9,60E-04 |
| Ppargc1a | peroxisome proliferative activated receptor, gamma, coactivator 1 alpha | -1,41 | 1,04E-03 |
| Socs2 | suppressor of cytokine signaling 2 | -1,40 | 3,10E-05 |
| Fbxo32 | F-box protein 32 | -1,40 | 2,27E-04 |
| Klk1b5 | kallikrein 1-related peptidase b5 | -1,40 | 4,16E-04 |
| Ppargc1a | peroxisome proliferative activated receptor, gamma, coactivator 1 alpha | -1,37 | 3,08E-05 |
| Cyp39a1 | cytochrome P450, family 39, subfamily a, polypeptide 1 | -1,33 | 3,94E-04 |
| Socs2 | suppressor of cytokine signaling 2 | -1,33 | 9,92E-04 |
| Cma1 | chymase 1, mast cell | -1,33 | 4,62E-04 |
| C4bp | complement component 4 binding protein | -1,31 | 1,24E-05 |
| Fbxl3 | F-box and leucine-rich repeat protein 3 | -1,28 | 2,07E-04 |
| Sh3rf2 | SH3 domain containing ring finger 2 | -1,24 | 5,24E-05 |
| Adamts15 | a disintegrin-like and metallopeptidase (reprolysin type) with thrombospondin type 1 motif, 15 | -1,24 | 2,00E-05 |
| Asb13 | ankyrin repeat and SOCS box-containing 13 | -1,23 | 8,90E-05 |
| Cd55 | CD55 antigen | -1,18 | 7,94E-04 |
| Klk1b4 | kallikrein 1-related pepidase b4 | -1,17 | 1,25E-03 |
| Mep1a | meprin 1 alpha | -1,14 | 1,71E-03 |
| Ctse | cathepsin E | -1,10 | 1,69E-05 |
| Agbl3 | ATP/GTP binding protein-like 3 | -1,09 | 4,75E-05 |
| Asb13 | ankyrin repeat and SOCS box-containing 13 | -1,09 | 3,50E-03 |
| Dpp4 | dipeptidylpeptidase 4 | -1,08 | 2,44E-03 |
| Aqp1 | aquaporin 1 | -1,08 | 2,97E-06 |
| Klk1 | kallikrein 1 | -1,06 | 1,35E-06 |
| March2 | membrane-associated ring finger (C3HC4) 2 | -1,05 | 8,89E-04 |
| Fbxl3 | F-box and leucine-rich repeat protein 3 | -1,04 | 4,59E-03 |
| Mep1a | meprin 1 alpha | -1,04 | 8,40E-06 |
| Pgcp | plasma glutamate carboxypeptidase | -1,03 | 4,68E-04 |
| Klk1 | kallikrein 1 | -1,02 | 6,96E-07 |
| Gm9706 /// Isg15 | predicted gene 9706 /// ISG15 ubiquitin-like modifier | 1,03 | 6,06E-05 |
| C1s | complement component 1, s subcomponent | 1,03 | 2,60E-05 |
| Adra2a | adrenergic receptor, alpha 2a | 1,07 | 9,37E-03 |
| C1qc | complement component 1, q subcomponent, C chain | 1,07 | 2,60E-05 |
| Rbp4 | retinol binding protein 4, plasma | 1,08 | 7,90E-05 |
| Cpn1 | carboxypeptidase N, polypeptide 1 | 1,08 | 3,28E-03 |
| C1qb | complement component 1, q subcomponent, beta polypeptide | 1,09 | 6,44E-03 |
| Brca1 | breast cancer 1 | 1,09 | 5,56E-05 |
| Lap3 | leucine aminopeptidase 3 | 1,09 | 1,61E-04 |
| C1qb | complement component 1, q subcomponent, beta polypeptide | 1,10 | 9,10E-04 |
| Mmp3 | matrix metallopeptidase 3 | 1,11 | 8,00E-05 |
| March1 | membrane-associated ring finger (C3HC4) 1 | 1,12 | 1,91E-03 |
| Mmp12 | matrix metallopeptidase 12 | 1,12 | 3,39E-03 |
| Cfd | complement factor D (adipsin) | 1,14 | 8,87E-06 |
| Gm10334 /// Gm5771 /// Prss1 /// Prss3 | predicted gene 10334 /// predicted gene 5771 /// protease, serine, 1 (trypsin 1) /// protease, serine, 3 | 1,15 | 4,61E-03 |
| Uhrf1 | ubiquitin-like, containing PHD and RING finger domains, 1 | 1,17 | 6,99E-05 |
| March9 | membrane-associated ring finger (C3HC4) 9 | 1,18 | 1,28E-03 |
| C1qb | complement component 1, q subcomponent, beta polypeptide | 1,19 | 1,66E-04 |
| Tff2 | trefoil factor 2 (spasmolytic protein 1) | 1,19 | 2,90E-03 |
| Hsp90b1 | heat shock protein 90, beta (Grp94), member 1 | 1,22 | 2,10E-04 |
| Gzmb | granzyme B | 1,24 | 2,10E-02 |
| Usp18 | ubiquitin specific peptidase 18 | 1,32 | 1,17E-03 |
| March9 | membrane-associated ring finger (C3HC4) 9 | 1,32 | 3,31E-03 |
| C3 | complement component 3 | 1,32 | 5,45E-05 |
| C1qa | complement component 1, q subcomponent, alpha polypeptide | 1,33 | 1,29E-05 |
| C4b | complement component 4B (Childo blood group) | 1,35 | 1,79E-06 |
| Srgn | serglycin | 1,36 | 4,40E-06 |
| Cuzd1 | CUB and zona pellucida-like domains 1 | 1,39 | 1,26E-03 |
| Usp11 | ubiquitin specific peptidase 11 | 1,42 | 1,75E-03 |
| Pla2g1b | phospholipase A2, group IB, pancreas | 1,44 | 1,26E-04 |
| Ctsc | Cathepsin C | 1,50 | 2,27E-03 |
| Psmb8 | proteasome (prosome, macropain) subunit, beta type 8 (large multifunctional peptidase 7) | 1,51 | 1,08E-02 |
| Clps | colipase, pancreatic | 1,53 | 3,26E-06 |
| Pappa | pregnancy-associated plasma protein A | 1,57 | 1,19E-03 |
| Ctsc | cathepsin C | 1,57 | 1,35E-04 |
| Mmp13 | matrix metallopeptidase 13 | 1,68 | 1,01E-04 |
| Trpv1 | transient receptor potential cation channel, subfamily V, member 1 | 1,69 | 1,50E-05 |
| Clec4a3 | C-type lectin domain family 4, member a3 | 1,74 | 1,74E-03 |
| Igh-3 /// Ighg | immunoglobulin heavy chain 3 (serum IgG2b) /// Immunoglobulin heavy chain (gamma polypeptide) | 1,75 | 2,97E-04 |
| Socs3 | suppressor of cytokine signaling 3 | 1,81 | 5,36E-06 |
| Mmp7 | matrix metallopeptidase 7 | 1,82 | 1,40E-04 |
| Tac1 | tachykinin 1 | 1,84 | 1,92E-06 |
| Ighg | Immunoglobulin heavy chain (gamma polypeptide) | 1,85 | 5,41E-06 |
| Clps | colipase, pancreatic | 1,92 | 7,92E-07 |
| Psmb8 | proteasome (prosome, macropain) subunit, beta type 8 (large multifunctional peptidase 7) | 1,96 | 2,70E-06 |
| Psmb9 | proteasome (prosome, macropain) subunit, beta type 9 (large multifunctional peptidase 2) | 1,99 | 4,11E-06 |
| Serpina3n | serine (or cysteine) peptidase inhibitor, clade A, member 3N | 2,08 | 7,77E-05 |
| Ubd | ubiquitin D | 2,41 | 1,31E-04 |
| Mcpt2 | mast cell protease 2 | 2,43 | 1,61E-08 |
| Gzma | granzyme A | 2,55 | 2,12E-03 |
| Mcpt1 | mast cell protease 1 | 2,83 | 1,49E-06 |
| Cpa2 | carboxypeptidase A2, pancreatic | 3,24 | 1,64E-05 |
| Socs3 | suppressor of cytokine signaling 3 | 3,26 | 7,00E-05 |
| Cela3b | chymotrypsin-like elastase family, member 3B | 3,26 | 1,72E-04 |
| Fabp1 | fatty acid binding protein 1, liver | 3,41 | 5,67E-03 |
| Socs3 | suppressor of cytokine signaling 3 | 3,49 | 1,89E-05 |
| Sct | secretin | 3,81 | 9,07E-07 |
| Pla2g1b | phospholipase A2, group IB, pancreas | 4,14 | 8,62E-04 |
| Cela3b | chymotrypsin-like elastase family, member 3B | 4,76 | 1,20E-05 |
| Cel | carboxyl ester lipase | 4,85 | 1,90E-06 |
| Ctrc | chymotrypsin C (caldecrin) | 4,86 | 2,14E-06 |
| Prss2 | protease, serine, 2 | 4,87 | 2,38E-04 |
| Ctrb1 | chymotrypsinogen B1 | 4,89 | 2,96E-04 |
| Prss2 | protease, serine, 2 | 4,95 | 2,67E-04 |
| Fabp1 | fatty acid binding protein 1, liver | 4,97 | 1,08E-03 |
| Prss2 | protease, serine, 2 | 5,02 | 1,22E-05 |
| Akr1c21 | aldo-keto reductase family 1, member C21 | 5,12 | 2,89E-04 |
| Ctrl | chymotrypsin-like | 5,21 | 1,46E-03 |
| Prss2 | protease, serine, 2 | 5,27 | 3,30E-04 |
| Cela3b | chymotrypsin-like elastase family, member 3B | 5,43 | 8,91E-08 |
| Cpa1 | carboxypeptidase A1 | 5,64 | 1,05E-04 |
| 2210010C04Rik | RIKEN cDNA 2210010C04 gene | 5,65 | 2,01E-03 |
| Pnlip | pancreatic lipase | 5,73 | 5,16E-04 |
| Cpb1 | carboxypeptidase B1 (tissue) | 7,24 | 6,42E-04 |
| Cela3b | chymotrypsin-like elastase family, member 3B | 7,33 | 9,41E-05 |
| Cela2a | chymotrypsin-like elastase family, member 2A | 7,40 | 4,15E-03 |
| Pnlip | pancreatic lipase | 7,42 | 1,38E-04 |
| 2210010C04Rik | RIKEN cDNA 2210010C04 gene | 8,19 | 9,98E-05 |
| Try4 /// Try5 | trypsin 4 /// trypsin 5 | 8,62 | 6,37E-05 |
| Cela3b /// Gm13011 | chymotrypsin-like elastase family, member 3B /// predicted gene 13011 | 9,21 | 1,65E-04 |
| Amy2a4 /// Amy2a5 | amylase 2a4 /// amylase 2a5 | 9,60 | 8,16E-05 |
