# Supplementary material for: HDAC1 and HDAC2 Restrain the Intestinal Inflammatory Response by Regulating Intestinal Epithelial Cell Differentiation
Source: PLoS One. 2013 Sep 5;8(9):e73785. doi: 10.1371/journal.pone.0073785 (PMC3764035; doi:10.1371/journal.pone.0073785)
Supplement: Table S5 — List of epithelial or epidermal development, and differentiation genes with significant 2-fold increased or decreased expression levels in HDAC1/2-depleted murine colons as determined by microarray analysis, and classified according to GO database. (DOCX) [file pone.0073785.s009.docx]

**Table S5**

List of epithelial or epidermal development, and differentiation genes with significant 2-fold increased or decreased expression levels in HDAC1/2-depleted murine colons as determined by microarray analysis, and classified according to GO database.

| epithelium development (GO:0060429); epithelial cell differentiation (GO:0030855);  epidermal cell differentiation (GO:0009913); epidermis development (GO:0008544) | | | |
| --- | --- | --- | --- |
|  |  |  |  |
| Gene Symbol | Gene Title | Fold change (log2) | P-value |
| Krt4 | keratin 4 | -3,33 | 2,18E-05 |
| Krt6b | keratin 6B | -3,19 | 6,74E-05 |
| Krt10 | keratin 10 | -3,15 | 7,14E-04 |
| Tgm3 | transglutaminase 3, E polypeptide | -2,93 | 3,39E-06 |
| Krtdap | keratinocyte differentiation associated protein | -2,90 | 2,60E-03 |
| Tgm3 | transglutaminase 3, E polypeptide | -2,66 | 8,75E-06 |
| H2-Q2 | histocompatibility 2, Q region locus 2 | -2,49 | 3,04E-03 |
| Krt6a | keratin 6A | -2,48 | 3,26E-03 |
| Scel | sciellin | -2,41 | 2,41E-04 |
| Gjb5 | gap junction protein, beta 5 | -2,39 | 1,02E-03 |
| Flg | filaggrin | -2,38 | 1,19E-03 |
| Foxq1 | forkhead box Q1 | -2,31 | 1,07E-05 |
| Sprr3 | small proline-rich protein 3 | -2,24 | 2,79E-03 |
| Krt4 | keratin 4 | -2,01 | 1,66E-04 |
| Prlr | prolactin receptor | -1,92 | 1,49E-03 |
| Lama3 | laminin, alpha 3 | -1,78 | 4,58E-04 |
| Krt1 | keratin 1 | -1,53 | 4,13E-02 |
| Calm4 | calmodulin 4 | -1,49 | 1,20E-03 |
| Krt14 | keratin 14 | -1,40 | 1,13E-04 |
| Gjb3 | gap junction protein, beta 3 | -1,36 | 9,32E-04 |
| Hdac1 | histone deacetylase 1 | -1,30 | 2,09E-05 |
| Prlr | prolactin receptor | -1,26 | 3,57E-05 |
| Acer1 | alkaline ceramidase 1 | -1,26 | 5,06E-04 |
| Prlr | prolactin receptor | -1,18 | 3,17E-03 |
| Ctse | cathepsin E | -1,10 | 1,69E-05 |
| Txnip | thioredoxin interacting protein | -1,05 | 2,11E-04 |
| Acer1 | alkaline ceramidase 1 | -1,05 | 6,29E-05 |
| Cyba | cytochrome b-245, alpha polypeptide | 1,03 | 2,19E-05 |
| Runx3 | runt related transcription factor 3 | 1,04 | 6,61E-05 |
| H2-DMa | histocompatibility 2, class II, locus DMa | 1,07 | 4,35E-03 |
| Ncf4 | neutrophil cytosolic factor 4 | 1,07 | 1,51E-03 |
| Cybb | cytochrome b-245, beta polypeptide | 1,07 | 1,44E-02 |
| Kif23 | kinesin family member 23 | 1,09 | 1,83E-03 |
| Fcgr3 | Fc receptor, IgG, low affinity III | 1,10 | 2,76E-03 |
| Ltb | lymphotoxin B | 1,10 | 2,04E-04 |
| Krt85 | keratin 85 | 1,10 | 8,54E-05 |
| Agpat2 | 1-acylglycerol-3-phosphate O-acyltransferase 2 (lysophosphatidic acid acyltransferase, beta) | 1,14 | 4,91E-04 |
| Was | Wiskott-Aldrich syndrome homolog (human) | 1,22 | 1,99E-03 |
| Fcer1g | Fc receptor, IgE, high affinity I, gamma polypeptide | 1,28 | 1,53E-05 |
| H2-Q10 | histocompatibility 2, Q region locus 10 | 1,29 | 2,98E-04 |
| H2-Ob | histocompatibility 2, O region beta locus | 1,32 | 1,14E-02 |
| H2-Ab1 | histocompatibility 2, class II antigen A, beta 1 | 1,40 | 3,76E-07 |
| Cybb | cytochrome b-245, beta polypeptide | 1,41 | 8,79E-05 |
| Fcgr2b | Fc receptor, IgG, low affinity IIb | 1,46 | 9,93E-04 |
| H2-DMb2 | histocompatibility 2, class II, locus Mb2 | 1,47 | 5,45E-03 |
| Ereg | epiregulin | 1,51 | 1,43E-04 |
| Psmb8 | proteasome (prosome, macropain) subunit, beta type 8 (large multifunctional peptidase 7) | 1,51 | 1,08E-02 |
| Akr1c18 | aldo-keto reductase family 1, member C18 | 1,62 | 2,77E-05 |
| H2-Aa | histocompatibility 2, class II antigen A, alpha | 1,64 | 2,51E-04 |
| Sprr1a | small proline-rich protein 1A | 1,64 | 5,21E-05 |
| Sfn | stratifin | 1,68 | 2,39E-06 |
| Trpv1 | transient receptor potential cation channel, subfamily V, member 1 | 1,69 | 1,50E-05 |
| Fst | follistatin | 1,72 | 2,00E-04 |
| H2-Ab1 | histocompatibility 2, class II antigen A, beta 1 | 1,76 | 1,47E-07 |
| Cd74 | CD74 antigen (invariant polypeptide of major histocompatibility complex, class II antigen-associated) | 1,83 | 2,30E-05 |
| H2-DMb2 | histocompatibility 2, class II, locus Mb2 | 1,87 | 9,98E-05 |
| Psmb8 | proteasome (prosome, macropain) subunit, beta type 8 (large multifunctional peptidase 7) | 1,96 | 2,70E-06 |
| H2-Ab1 | histocompatibility 2, class II antigen A, beta 1 | 1,98 | 6,75E-07 |
| Psmb9 | proteasome (prosome, macropain) subunit, beta type 9 (large multifunctional peptidase 2) | 1,99 | 4,11E-06 |
| H2-DMb2 | histocompatibility 2, class II, locus Mb2 | 2,00 | 1,22E-05 |
| H2-Aa | histocompatibility 2, class II antigen A, alpha | 2,13 | 1,43E-04 |
| H2-DMa | histocompatibility 2, class II, locus DMa | 2,22 | 3,95E-05 |
| H2-Aa | histocompatibility 2, class II antigen A, alpha | 2,24 | 2,41E-04 |
| H2-Eb1 | histocompatibility 2, class II antigen E beta | 2,27 | 4,19E-05 |
| H2-DMb1 /// H2-DMb2 | histocompatibility 2, class II, locus Mb1 /// histocompatibility 2, class II, locus Mb2 | 2,32 | 4,77E-05 |
| H2-Ea-ps /// LOC100504404 | histocompatibility 2, class II antigen E alpha, pseudogene /// h-2 class II histocompatibility antigen, E-K alpha chain-like | 2,33 | 1,15E-05 |
| H2-DMb1 /// H2-DMb2 | histocompatibility 2, class II, locus Mb1 /// histocompatibility 2, class II, locus Mb2 | 2,52 | 1,81E-04 |
| H2-Aa | histocompatibility 2, class II antigen A, alpha | 2,54 | 2,56E-03 |
| Krt36 | keratin 36 | 2,68 | 4,39E-05 |
| Foxn1 | forkhead box N1 | 3,03 | 1,85E-04 |
| Cyp2c65 | cytochrome P450, family 2, subfamily c, polypeptide 65 | 3,08 | 9,86E-07 |
| H2-Ea-ps /// LOC100504404 | histocompatibility 2, class II antigen E alpha, pseudogene /// h-2 class II histocompatibility antigen, E-K alpha chain-like | 3,27 | 2,16E-05 |
| Sprr2h | small proline-rich protein 2H | 4,66 | 3,97E-04 |
| Krt84 | keratin 84 | 4,73 | 8,13E-06 |
